# Comprehensive exploration of JQ1 and GSK2801 targets in breast cancer using network pharmacology and molecular modeling approaches

**DOI:** 10.1016/j.csbj.2023.06.003

**Published:** 2023-06-03

**Authors:** Nanda Kumar Yellapu, Dong Pei, Emily Nissen, Jeffrey A. Thompson, Devin C. Koestler

**Affiliations:** Department of Biostatistics & Data Science, University of Kansas, Medical Center, Kansas City, KS, USA

**Keywords:** Network pharmacology, Breast cancer, Protein-Protein Interaction network maps, Drug targets

## Abstract

JQ1 and GSK2801 are bromo domain inhibitors (BDI) known to exhibit enhanced anti-cancer activity when combined with other agents. However, the underlying molecular mechanisms behind such enhanced activity remain unclear. We used network-pharmacology approaches to understand the shared molecular mechanisms behind the enhanced activity of JQ1 and GSK2801 when used together to treat breast cancer (BC). The gene targets of JQ1 and GSK2801 were intersected with known BC-targets and their putative targets against BC were derived. The key genes were explored through gene-ontology-enrichment, Protein-Protein-Interaction (PPI) networking, survival analysis, and molecular modeling simulations. The genes, CTSB, MAPK14, MET, PSEN2 and STAT3, were found to be common targets for both drugs. In total, 49 biological processes, five molecular functions and 61 metabolic pathways were similarly enriched for JQ1 and GSK2801 BC targets among which several terms are related to cancer: IL-17, TNF and JAK-STAT signaling pathways. Survival analyses revealed that all five putative synergistic targets are significantly associated with survival in BC (log-rank *p* < 0.05). Molecular modeling studies showed stable binding of JQ1 and GSK2801 against their targets. In conclusion, this study explored and illuminated the possible molecular mechanisms behind the enhanced activity of JQ1 and GSK2801 against BC and suggests synergistic action through their similar BC-targets and gene-ontologies.

## Highlights

• CTSB, MAPK14, MET, PSEN2 and STAT3 genes are the common BC targets for JQ1&GSK2801.
• JQ1&GSK2801 form stable intermolecular connections and binding with BC targets.
• JQ1 & GSK2801 shows synergistic activity probably through STAT3 gene.

## Graphical Abstract

**Figure:**
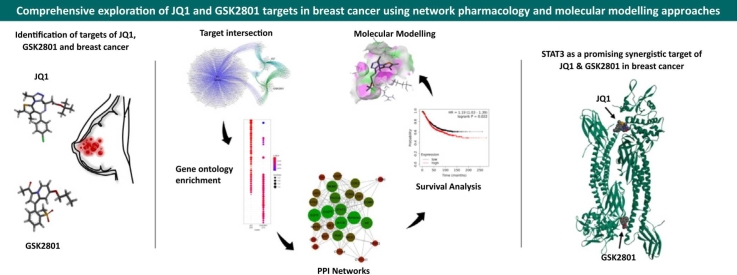

## 1 Introduction

Breast Cancer (BC) remains a substantial global health challenge and currently represents the most commonly diagnosed cancer worldwide after skin cancer. Despite enormous progress in understanding its molecular pathogenesis and progression, BC stands as the second leading cause of cancer deaths in the United States with a current annual death rate of 40,000 women [39], [36], [71]. The survival rates are far less promising in under-developed regions. Among the 685,000 deaths worldwide reported in 2020, two-thirds were from under-developed regions [34]. Despite significant progress in treating and managing BC, existing therapies are not 100% successful [23]. Further, relapse and progression remain a large concern [41], [27]. Taken together with the public health and personal burden associated with BC, there remains a pressing need for future research aimed at improving early detection, along with development of novel effective treatment strategies. The past several decades has witnessed an enormous increase in knowledge regarding the molecular mechanisms underlying BC pathogenesis and progression and such insights have been leveraged in the development of novel therapeutics.

**Fig. 1:**
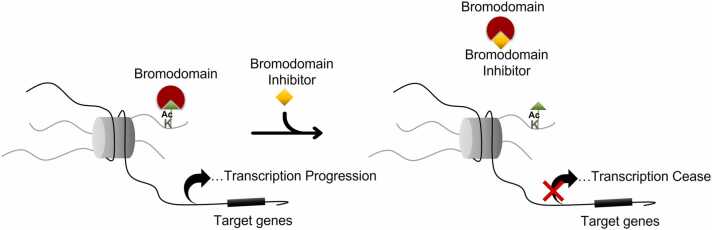
Mechanism of action of Bromodomains and Bromodomain inhibitors. Bromodomains recognize the acetylated lysine residues on the histone tails and induce the transcriptional machinery of target genes to progress. Bromodomain inhibitors prevent the association of bromodomain and acetyl groups ceasing the transcription process.

Research in recent years has highlighted Bromodomains (BD) as potential therapeutic targets for several types of cancers, inflammatory diseases, and autoimmune disorders [65], [19], [28], [57], [73]. BDs bind to the acetylated lysine residues of histone proteins and regulate gene expression by recruiting several molecular partners (Fig. 1) [18], [46]. The majority of BD family proteins are associated with the transcriptional regulation of cancer related genes and dissociation of BDs with their target genes leads to anti-cancer activity. Hence, proteins with BDs represent potential drug targets, leading to the development of Bromodomain inhibitors (BDI) [7], [58], [11], [74] as therapeutic agents. Over the past decade, there has been an increasing demand to develop small molecule BDIs. Currently, several clinical trials of BDIs, representing the novel family of molecules for cancer-targeted therapy, are in progress[2]. Despite these promising developments, single agent inhibitors are not completely successful in repressing the expression of the target oncogenes. Therefore, research is turning to the combinatorial use of BDIs.

There are a number of studies that support the use of BDIs in combination with other therapeutics. Langdon et al., observed that dual bromodomain/neddylation blockade inhibits the in vivo growth of pancreatic adenocarcinoma (PDAC) cell line xenografts by JQ1/MLN4294 [30]. Shu et al., investigated the heterogeneity of cellular responses and resistance to JQ1 in triple negative breast cancer (TNBC) and identified promising combination therapies with bromodomain and extraterminal (BET) domain BDIs that could be used for more effective treatment of TNBC, resistant to chemotherapy [62]. Several studies have reported on the synergistic efficacy of Histone deacetylase (HDAC) inhibitors when combined with BDIs against Glioblastoma (GBM) cells [43], [1], [76]. Siegel et al., combined a BDI, CPI203, with bortezomib, and reported an enhanced efficacy against drug resistant multiple myeloma [63]. Wilson et al., reported the synergistic activity of JQ1 with Olaparib and reported the suppression of growth of BRCA-proficient cancer in vivo in a xenograft ovarian cancer mouse model [72]. Liu et al., investigated the synergistic effect of Vincristine, an FDA-approved drug with JQ1 and reported the suppression of neuroblastoma progression in mice [38]. Studies investigating the synergistic effect of the BDIs against different cancers are progressively increasing.

**Fig. 2:**
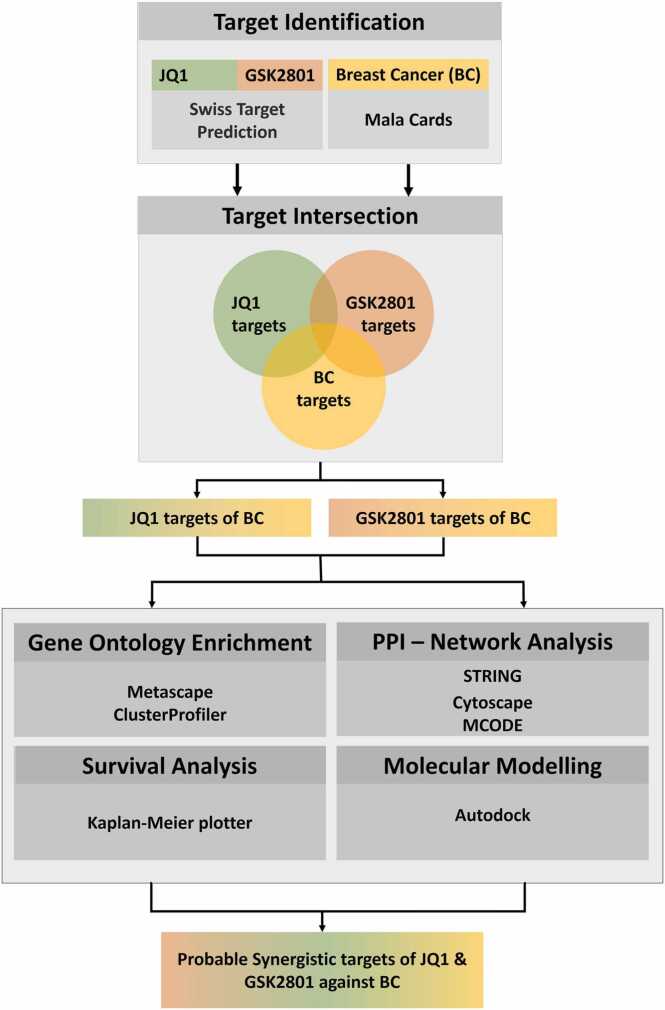
Outline of the methods and tools followed in the current study.

In our recent study, we evaluated the effect of two popular BDIs, JQ1 and GSK2801 against three types of BC cell lines using multiple dose combinations [75]. We observed stronger effects when the two agents were combined as compared to when they were given as single agent treatments. Previously, Bevill et al., reported that JQ1 synergizes with GSK2801 enhancing anti-cancer activity against TNBC [7], [8], [9]. Observations from our previous study [75] along with the reports from Bevill et al., support their ability to induce the synergistic activity when they are used in combination. However, the molecular mechanism behind the enhanced activity and the factors that drive synergistic behavior among the combined drugs is still unclear. BDIs with anti-cancer activity may show drug resistance in specific patients and be sensitive in other patients. This is due to variable targets for the drugs that defines the sensitivity. BDIs may exhibit anti-cancer activity through several different mechanisms associated with variable targets. Designing precise and effective combination therapies requires information about the molecular mechanism of BDIs (i.e., their targets of action) in order to achieve desired results and also combat drug resistance. To address these aforementioned aspects, we have employed a series of innovative methodologies in contrast to our previous investigation. In this study, instead of focusing solely on TNBC, we have encompassed the entire set of targets associated with diverse subtypes of BC sourced from publicly available data repositories. This comprehensive approach facilitates the elucidation of the broader landscape responsible for the synergistic effects of drugs in BC as a whole. Subsequently, we performed an overlapping analysis between the global BC targets and the overall targets of JQ1 and GSK2801, thereby narrowing down the plausible number of synergistic targets. Using these data sets, in the current study, we aimed to identify the molecular mechanisms behind the synergistic action of JQ1 and GSK2801 that is responsible for the enhanced activity observed in their combinatorial use against BC. Specifically, we applied network pharmacology approaches to the putative gene targets of JQ1 and GSK2801, intersected with BC target genes. The global picture of their targets against BC was constructed as network model and subjected to clustering process. Application of molecular modeling methods to the identified targets was undertaken to determine the binding mode orientations and affinities of JQ1 and GSK2801 helps to derive probable evidence for their molecular mechanism of action [48]. This approach is now widely accepted as a tool to demonstrate the impact of drugs in biological systems [17], [15], [67]. Application of such approaches in the current study helped to elucidate the molecular machinery behind the synergistic effect of JQ1 and GSK2801 against BC. The entire process and methods are outlined as Fig. 2.

## 2 Methods

### 2.1 JQ1 and GSK2801 target prediction

Structural information for JQ1 (PubChem ID: 46907787) and GSK2801 (PubChem ID: 73010930) was derived from NCBI-PubChem database [29]. The structures of JQ1 and GSK2801 were submitted to SwissTargetPrediction database (http://www.swisstargetprediction.ch/) in their simplified molecular-input line-entry system (SMILES) format, which maps the targets of bioactive compounds to unravel the molecular mechanisms underlying their bioactivity. SwissTargetPrediction predicts the targets of bioactive compounds based on the combination of 2D and 3D similarity structural features with known ligands [20].

### 2.2 Breast cancer targets

The MalaCards database (https://www.malacards.org/) was used to derive the list of BC targets. MalaCards is a robust database hosting the universe of genes associated with several human diseases[55]. The search term “breast cancer” was used to obtain BC targets and the results were standardized as official human gene symbols as per HUGO Gene Nomenclature Committee (HGNC).

### 2.3 Target intersection and network map construction

The targets of JQ1 and GSK2801 were intersected with BC targets using the FunRich analysis tool (http://www.funrich.org/). The number of intersected targets were visualized using Venn diagrams and the individual list of targets was used to construct an intersection network map using the Cytoscape software [61]. A graphical compound target-network was established to visualize the overlapping targets of JQ1, GSK2801 and BC. The common BC targets for JQ1 and GSK2801 drugs were located as sub-networks and defined as possible synergistic targets.

### 2.4 Gene enrichment analysis

The potential targets of JQ1 and GSK2801 against BC were provided as input to the Metascape (https://metascape.org/) database to identify gene ontology (GO) terms such as, biological process, molecular functions, and metabolic pathways that were significantly enriched with such targets. The enrichment results were ranked according to their significance and the results were visualized using the ClusterProfiler R-package [77]. Bar plots were derived for the top 25 categories of biological process, molecular functions, and metabolic pathways based on their smallest adjusted p-values derived from Bonferroni method.

### 2.5 Protein-protein interaction network analysis

The protein-protein interaction (PPI) network maps were constructed for the potential BC targets of JQ1 and GSK2801 independently using the STRING database [66] plugin in Cytoscape. PPI maps of JQ1 and GSK2801 targets were visualized and analyzed separately. In such PPI maps, proteins are represented as nodes and their interactions, as edges. Each PPI map was further subjected to topological clustering analysis using the Molecular Complex Detection (MCODE) plugin [3]. The idea behind this algorithm is that the densely connected regions in the PPI are likely to represent molecular complexes. The PPI networks were filtered with degree cutoff= 2, node score cutoff= 0.2 and K-core= 2 with maximum depth= 100 to derive the significant modules. The significant clusters were further subjected to CytoHubba [12] to rank their nodes by various network features in order to identify key nodes. The degree and betweenness centrality were calculated to define the role of a node in the network and to identify the putative targets of JQ1 and GSK2801 against BC.

### 2.6 Survival analysis

Putative synergistic targets from the PPI network analysis served as candidate genes to carry out survival analyses to study their relationship with the patient outcomes in BC. Kaplan-Meier survival plots for overall survival were generated for each target separately using Kaplan-Meier plotter (https://kmplot.com/) [22] along with RNAseq data and clinical data from National Center for Biotechnology Information - Gene Expression Omnibus (NCBI-GEO) and The Cancer Genome Atlas (TCGA) databases [10]. Log-rank tests were performed to test for associations between gene expression and survival.

### 2.7 Molecular modeling

The binding mode orientations and affinities of JQ1 and GSK2801 against their targets were studied by molecular modeling studies using the Autodock Vina software[47]. The three-dimensional structures of JQ1 and GSK2801 were optimized, and energy minimized using the MM2 force field. The crystallographic structures of the targets were retrieved from the Protein Data Bank (PDB) [6]. The protein structures were prepared by removing heteroatoms and water molecules, adding polar hydrogens, and finally energy minimizing the structures to an RMS gradient of 0.1 Å. The active site information was explored from PDBSum[32] for each structure and defined the docking grid to dock the ligands. The docking poses were ranked based on the docking scores and the pose with lowest energy was saved and the protein-ligand interactions were studied.

## 3 Results

### 3.1 BC targets for JQ1 and GSK2801

**Fig. 3:**
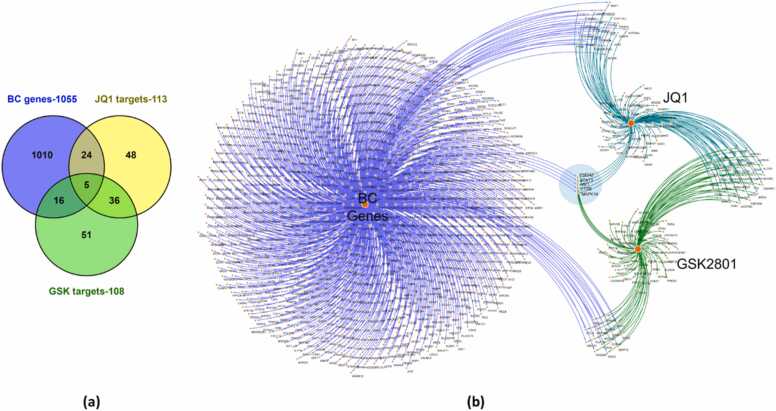
Target intersection analysis. (a). Venn diagram representing the intersection of number of target genes of JQ1 and GSK2801 against BC targets. (b). Compound-target interaction network showing the overlapping targets of JQ1 and GSK2801 against BC targets. BC targets are connected with violet, JQ1 targets with teal, and GSK2801 targets with green color edges. The intersection shows five common targets (CTSB, MAPK14, MET, PSEN2 and STAT3) of JQ1 and GSK2801 against BC genes. The gene list is provided in the Supplementary material files S1, S2, S3 and S4.

The chemical scaffolds of JQ1 and GSK2801 were utilized by the SwissTargetPrediction [20] database, and their biological targets of JQ1 and GSK2801were predicted. After removing duplicates, 113 targets were identified for JQ1 and 108 for GSK2801. Meanwhile, 1055 target genes associated with BC were obtained from MalaCards database. The intersection of JQ1 and GSK2801 putative targets against BC targets revealed that among the 113 targets of JQ1, 29 overlapped with the 1055 BC targets, whereas among the 108 targets of GSK2801, 21 overlapped with BC targets (Fig. 3**.a)**. The list of JQ1 targets is provided as Supplementary material file S1, GSK2801 targets as Supplementary material file S2, BC genes as Supplementary material file S3**.** Among the 29 and 21 overlapping targets for JQ1 and GSK2801, respectively, five genes: CTSB, MAPK14, MET, PSEN2 and STAT3, were shared between JQ1 and GSK2801 (Fig. 3**.a,** Supplementary material file S4). A compound-target network of JQ1/GSK2801 against BC was constructed using Cytoscape where the compounds and their candidate targets are represented as nodes and each target is linked with the compounds with edges (Fig. 3**.b**). The measure of node importance is based on its degree of interaction with each other nodes in the network. The interactions in Fig. 3**.b** show that both JQ1 and GSK2801 may act on multiple distinct BC targets, but also share several of the same targets. The results from the target intersection analysis offers some insight into the synergistic relationship between JQ1 and GSK2801 as both share five common BC targets in the network, indicating possible similar mechanisms of action.

### 3.2 Gene enrichment analysis of target genes

**Fig. 4:**
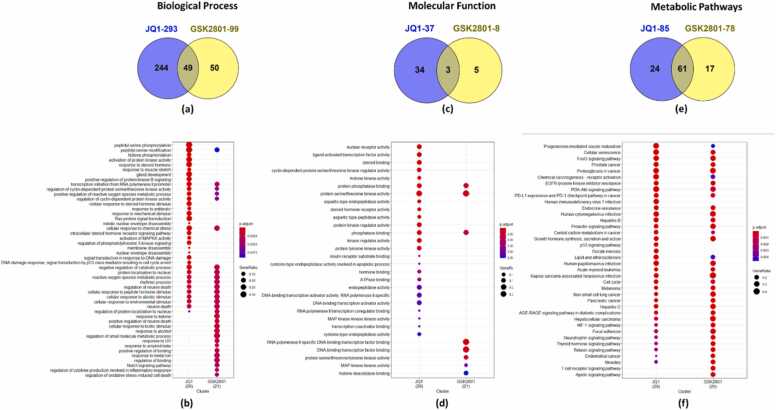
Cluster comparisons with respect to the GO enrichment analysis of JQ1 and GSK2801 BC targets. (a) Venn diagram showing the intersection of number of biological processes enriched with JQ1 and GSK2801 BC targets. (b) Bubble chart showing the comparison of JQ1 and GSK2801 clusters of biological processes. (c) Venn diagram showing the intersection of number of molecular functions enriched with JQ1 and GSK2801 BC targets. (d) Bubble chart showing the comparison of JQ1 and GSK2801 clusters of molecular functions. (e) Venn diagram showing the intersection of number of metabolic pathways enriched with JQ1 and GSK2801 BC targets. (f) Bubble chart showing the comparison of JQ1 and GSK2801 clusters of metabolic pathways. The top terms are represented in the bubble charts along with their gene ratios as per significant p-adj values.

The JQ1 and GSK2801 targets against BC were subjected to enrichment analysis and the top GO terms for biological processes, molecular function, and metabolic pathways are represented as gradient bar charts, rank-ordered based on their adjusted p-values (Supplementary material file S7, Table S1). The GO enrichment results of JQ1 targets revealed that its targets are primarily associated with terms like: histone phosphorylation, protein kinase activity, MAPK-Kinase activity, membrane activities, transcription regulation, serine/threonine kinase activities, FoxO signaling, PI3K-Akt signaling, p53 signaling and several other cancer related biological functions and pathways. The BC targets of GSK2801 were observed to associate with GO terms: notch signaling, transcription regulation, MAPK-signaling, p53 signaling, FoxO signaling, AGE-RAGE signaling, T-cell receptor signaling and HIF-1 signaling pathways etc. These pathways have important role in cancer pathogenesis. Importantly, there are GO terms which overlap between JQ1 and GSK2801 BC targets. Hence, a cluster comparison of the GO enrichment results was carried out, revealing 49 biological processes, 3 molecular functions, and 61 metabolic pathways that are commonly enriched with JQ1 and GSK2801 targets. Cluster comparisons are visualized in Fig. 4. The shared terms among the clusters of JQ1 and GSK2801 suggest that they target similar functions which could be responsible for their synergistic action.

### 3.3 Protein-protein interaction networks of JQ1 and GSK2801 targets against BC

**Fig. 5:**
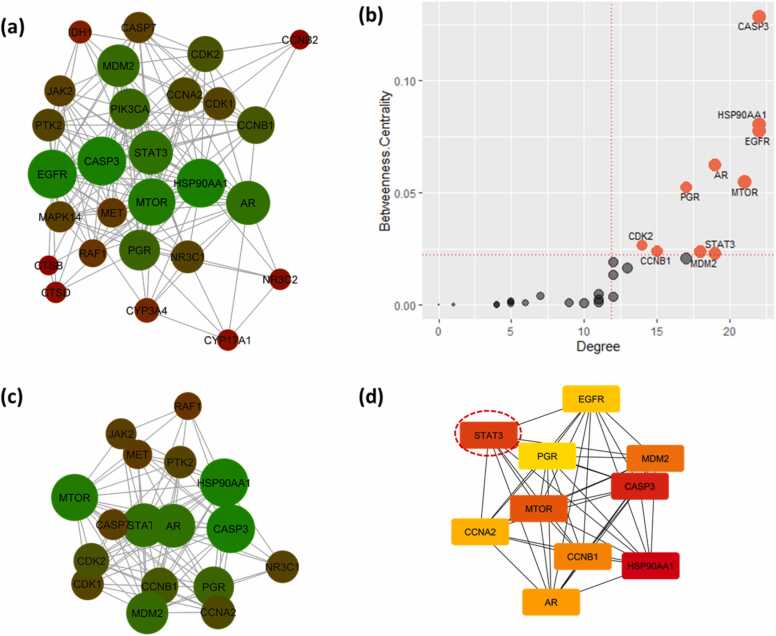
Protein-protein interaction network analysis of JQ1 BC targets. (a). The PPI network map of JQ1 targets against BC. The degree of nodes is represented as red to green and smaller to bigger indicating the increasing order of degree of interaction. Each node represents a protein and the edges protein-protein interactions. (b). Node betweenness centrality and degree distribution of the JQ1-PPI network. The average degree is 11.861 and average betweenness centrality is 0.022. Ten targets were produced betweenness centrality and degree values above the average and it is suspected that these targets encode proteins with key roles in BC. (c). The single biggest cluster identified from the MCODE cluster analysis representing the densely connected molecular complex of the PPI network. (d). The top 10 nodes extracted from the CytoHubba analysis of PPI network representing the core density of the network. The ranking was given from Red to Yellow (1−10). The synergistic target identified from the target intersection is shown in red dotted circle.

**Fig. 6:**
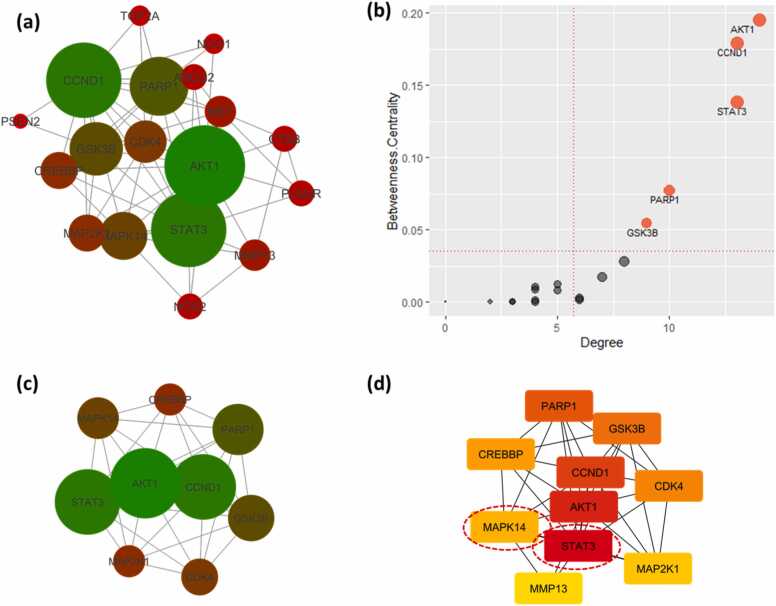
Protein-protein interaction network analysis of GSK2801 BC targets. (a). The PPI network map of GSK2801 targets against BC. The degree of nodes is represented as red to green and smaller to bigger indicating the increasing order of degree of interaction. Each node represents a protein and the edges protein-protein interactions. (b). Node betweenness centrality and degree distribution of the GSK2801-PPI network. The average degree is 5.714 and average betweenness centrality is 0.035. Five targets were produced betweenness centrality and degree values above the average and it is suspected that these targets encode proteins with key roles in BC. (c). The single biggest cluster identified from the MCODE cluster analysis representing the densely connected molecular complex of the PPI network. (d). The top 10 nodes extracted from the CytoHubba analysis of PPI network representing the core density of the network. The ranking was given from Red to Yellow (1−10). The synergistic targets identified from the target intersection are shown in dashed red circles.

Individual PPI network maps were constructed for the BC targets of JQ1 (Fig. 5**.a**) and GSK2801 (Fig. 6**.a**). The degree of a node in the network indicates the number of connections with other nodes of the network and the betweenness centrality explains the importance of a node in connecting other nodes in the network. The larger the degree and betweenness centrality, the greater the interaction of that target with other targets. Any modulation of such targets would impact the next level nodes connected downwards to them. Such targets represent the essential nodes of the network. The JQ1 PPI network maps showed an average degree of 11.861 and an average betweenness centrality of 0.022. There are 10 gene targets, CASP3, HSP90AA1, EGFR, AR, MTOR, PGR, CDK2, CCNB1, STAT3 and MDM2, that were identified above these averages (Fig. 5**.b**). The PPI network map of GSK2801 BC targets showed an average degree of 5.714 and an average betweenness centrality of 0.035. There are five gene targets: AKT1, CCND1, STAT3, PARP1 and GSK3B, that were identified above the average degree and betweenness centrality calculated for the network (Fig. 6**.b**). It is also noteworthy that among the five proposed synergistic targets from the target intersection, STAT3 was observed among the most important targets in both JQ1 and GSK2801 PPI networks.

The PPI networks were further analyzed to extract the significant clusters. MCODE analysis revealed a single major cluster from each of JQ1 and GSK2801 PPI networks (Fig. 5**.c** and Fig. 6**.c**). These clusters are the densely connected regions of the network representing possible molecular interactions. They are identified utilizing the connectivity scores of the network. In addition to MCODE clustering, the networks were subjected to CytoHubba ranking to derive the top ten essential nodes of the network (Fig. 5**.d** and Fig. 6**.d**). The top ten nodes were ranked based on their core densities and the topological contiguity. Higher rank represents the more concentrated nodes of the network. Interestingly, we observed that the top ten essential nodes extracted from the PPI network maps were also observed in the MCODE clusters, supporting their key role in the network. Another observation from the clustering analysis is the identification of STAT3 in the MCODE and CytoHubba clusters of both JQ1 and GSK2801. This further highlights STAT3 as a potential target of synergistic activity.

### 3.4 Survival analysis of putative synergistic targets

**Fig. 7:**
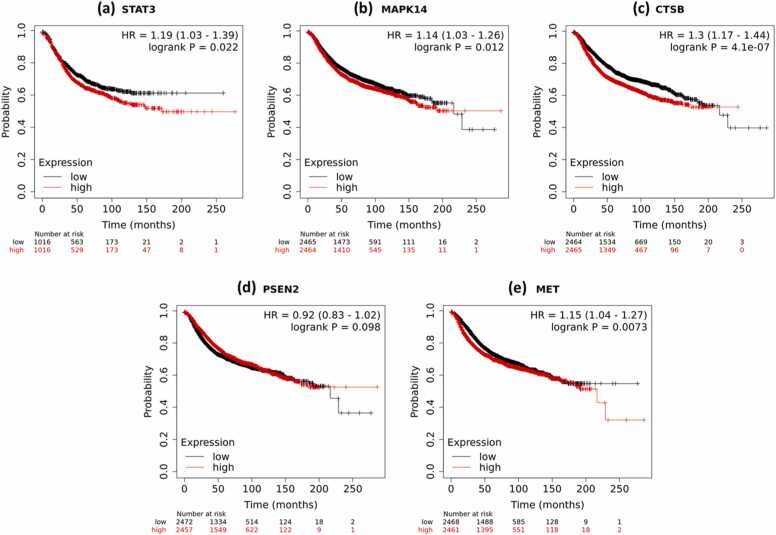
Survival analysis of five putative synergistic targets. Kaplan-Meier plots of overall survival of the identified putative synergistic targets. High/low expression is defined based on the median expression values of each gene.

Survival analysis was performed for the five putative synergistic targets identified from the target intersection i.e. CTSB, MAPK14, MET, PSEN2 and STAT3, in order to assess whether the expression of these genes is associated with overall survival among BC patients. The expression data for these five genes were used from the NCBI-GEO and TCGA databases and it was observed that all were significantly associated with overall survival (Fig. 7) at p < 0.05 except PSEN2, which was marginally significantly associated with survival (p = 0.098). Interestingly, STAT3 which was among the five putative synergistic targets had the highest betweenness centrality and degree in the PPI network maps of both JQ1 and GSK2801 BC targets, was also observed as potential prognostic risk factor for overall survival in the context of BC, suggesting its potential important role in the response of BC to JQ1 and GSK2801, synergistically.

### 3.5 Molecular docking studies

**Fig. 8:**
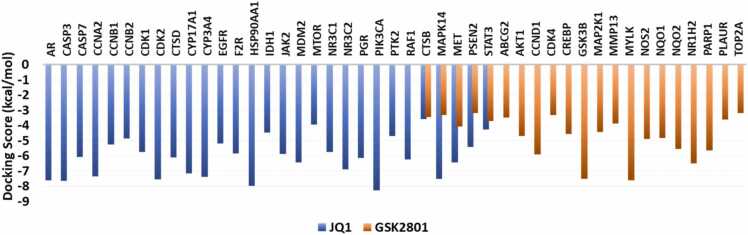
Molecular Docking scores of JQ1 and GSK2801 against their BC targets. The binding affinity of JQ1 and GSK2801 is represented as docking scores for each of their BC targets. There are five targets that are commonly targeted by both the drugs.

Molecular docking studies support further validation of effect in JQ1 and GSK2801 against BC through the identified targets. The affinity and binding modes of the JQ1 and GSK2801 were explored against their BC-specific targets. The docking scores are represented as bar graphs (Fig. 8). Stable binding energies were observed in each of the docking complexes suggesting the strong binding of the compounds against their BC targets. The binding orientations and the intermolecular connections of JQ1 and GSK2801 are provided as Supplementary material file S7**,** Table S2 and S3. The affinities and binding orientation of JQ1 and GSK2801 suggest their inhibitory action against these BC target proteins and their potential therapeutic role in BC pathogenesis.

## 4 Discussion

JQ1 and GSK2801 are well known BDIs found to be active against several types of cancers, however the molecular mechanisms underlying their combinatorial use is not well characterized. We previously observed enhanced anti-cancer activity of these two drugs in three types of cancer cell lines, MDA-MB-231, HCC-1806 and SUM-159, when they were combined rather than as single agents [75]. Hence, our current study is aimed at elucidating the possible molecular mechanisms behind their enhanced anti-cancer activity in BC using network pharmacology approaches. Network pharmacology combined with molecular modeling methods represent an intersection of multidisciplinary research areas including pharmacology, molecular biology, and bioinformatics. Network pharmacology methods helps to identify the targets and pathways illuminating the drug-target interactions in the biological systems [33], [42], [78], [24]. Such network pharmacology approaches have been efficiently utilized to discover and explore the therapeutic role and molecular mechanisms of several drugs at once, across various disease conditions [48], [15], [33], [42], [78]. In the current study, we used this approach to discover the possible molecular mechanisms behind the synergistic role of two BDIs, JQ1 and GSK2801, in BC. Our primary investigations based on the studies from Bevil et al., [8] provided several important clues and supporting evidence on the synergistic role of these two drugs against BC [75]. These results were the inspiration for the current study in which we aimed to identify and characterize potential molecular targets underlying the synergistic action of these two drugs.

We identified putative BC-specific targets of JQ1 and GSK2801 using the SwissTargetPrediction and MalaCards databases. The intersection of JQ1 and GSK2801 putative BC targets gave some clues to the possible synergistic action of these two drugs by revealing CTSB, MAPK14, MET, PSEN2 and STAT3 genes as common BC targets. These five genes were observed to be associated with several cancer related metabolic pathways and biological processes and were identified as hotspot nodes in the PPI network maps, each with high degree of connectivity. For this reason, these five putative targets were further investigated for their potential prognostic role in BC. With the exception of PSEN2, the expression of these five genes were significantly associated with overall survival in the context of BC. CTSB has been reported as a potential prognostic marker for BC where it is involved in cancer progression and invasion [50], [64], [60]. MAPK14 gene pathways are associated with several cellular processes involved in breast carcinogenesis and have been highlighted as a potential target for treating BC [13]. Further, several studies utilizing network pharmacology approaches have reported MAPK14 as a hub gene in PPI network maps of BC [26], [70]. MET is a proto-oncogene, receptor tyrosine kinase and its altered expression leads to proliferation, migration, invasion, metastatic spread, and neo-angiogenesis of cancer cells [44], [21]. There are fewer reports available on PSEN2 association with BC, but some studies have reported an association with Alzheimer's disease [14], [56], [31]. Several patients with R62H and R71W variants in PSEN2 gene were diagnosed with BC suggesting its role in BC pathogenesis [68], [54]. But the expression of PSEN2 was observed to not significantly impacting the survival probabilities of BC patients. This suggests PSEN2 may be given priority for further investigation of its role in the molecular pathogenesis in BC. In the current study, PSEN2 is observed as a major potential target in BC for JQ1 and GSK2801 giving a possible lead. The final synergistic target identified in the current study is STAT3, a well-known prognostic marker in BC. There are numerous reports on STAT3 in BC as an early-stage tumor diagnostic marker and it is known to induce BC malignancy [40], [25], [52], [59]. The over expression of STAT3 is responsible for proliferation, progression, metastasis and also resistance to chemotherapy [40], [4]. Small molecular inhibitors targeting STAT3 overexpression have been suggested as a promising treatment for BC [53]. It is interesting that in our current study, STAT3 has been observed as potential target for both JQ1 and GSK2801, supporting the validity of our results.

The GO enrichment analysis conducted on the putative BC targets of JQ1 and GSK2801 revealed several major BC associated pathways such as: PI3-Akt signaling pathway [51], MAPK signaling pathway [49], serine/threonine kinase pathway [45], FoxO signaling pathway [16], [37], Notch signaling [5], p53 signaling [69] and HIF-1 signaling [79]. Importantly, several biological processes and metabolic pathways are shared by both drugs indicating their synergistic action as observed from the target intersection analysis (Fig. 4).

Among the five proposed synergistic target genes, STAT3 was observed with betweenness centrality and degree values above the average in the PPI networks of JQ1 and GSK2801 targets (Fig. 5**.b** and Fig. 6**.b**). This indicates its potential important role in the network for both the drugs [35]. This observation acts as supporting evidence for considering STAT3 as a plausible synergistic target for JQ1 and GSK2801. This was further strengthened from the clustering analysis of PPI networks where STAT3 was observed in the significant clusters extracted from MCODE clustering and placed among the top ten important nodes of CytoHubba ranking. All these investigations suggest that JQ1 and GSK2801 may act synergistically against BC through STAT3.

The survival analysis shows that over expression of STAT3 and other proposed synergistic targets is associated with reduced survival probability of BC patients. Molecular modeling studies further support the inhibitory action of JQ1 and GK2801 against all the identified targets along with the synergistic targets. These molecular docking studies were aligned with our previous studies where JQ1 and GSK2801 showed their stable interactions and binding with PTPRC, MUC19, KCNB1, TAGLN, and KISS1 proteins whose gene expression was found to be downregulated in the three TNBC cell lines [75]. The molecular docking studies were performed using Schrodinger’s Maestro software in the previous study, still the binding efficiencies were found to be stable for JQ1 and GSK2801 in either of the studies. Further, the downregulation of the expression of these genes was validated when the cell lines were treated with JQ1 and GSK2801. These studies provide possible evidence about the therapeutic role of JQ1 and GSK2801 both as independent and as synergistic agents where the synergism has been predicted to involve specific molecular targets such as CTSB, MAPK14, MET, PSEN2 and STAT3. Among these five proposed synergistic targets, STAT3 has more convincing evidence in each step of investigations for being a reliable target responsible for the synergistic action of the JQ1 and GSK2801. Overall, the current study helps to predict the possible molecular mechanism behind the synergistic action of JQ1 and GSK2801 and suggests their combinatorial use as promising therapeutic strategy after suitable in vitro and in vivo validation of these results. However, to translate these findings into potential clinical applications, it is crucial to validate the predicted synergistic targets of JQ1 and GSK2801 through gene expression studies in relevant breast cancer cell line models. Specifically, investigations into how the drugs modulate the expression of these gene, individually and in combination, will provide valuable insights into the molecular mechanisms underlying the synergistic effects of these drugs. It will also enhance the understanding of underlying molecular mechanisms that drive the synergistic activity of JQ1 and GSK2801 in BC. This work provides a solid foundation for future investigations, which ideally include in vivo studies and preclinical studies aimed at evaluating the therapeutic potential of these drugs with the goal of refining treatment strategies for BC patients.

## 5 Conclusion

This study utilized network pharmacology and molecular modeling approaches with the aim of illuminating the potential molecular and pharmacological mechanisms of JQ1 and GSK2801 against BC. Our investigation identified CTSB, MAPK14, MET, PSEN2, and STAT3 genes as potential synergistic targets of JQ1 and GSK2801 in BC. These targets serve as primary leads for the anti-cancer activity of JQ1 and GSK2801 against BC. Arrival at these five potential targets was based on an intersection and clustering analysis of PPI networks derived from the target genes from reliable resources are responsible for the synergistic role of JQ1 and GSK2801. STAT3, one of five synergistic targets in the current study was observed as a common element with the highest betweenness centrality and degree in the PPI network clusters of JQ1 and GSK2801 targets. This provides convincing evidence for STAT3 to be an important element driving the molecular mechanisms of synergistic action of JQ1 and GSK2801. The next step to advance this research is to validate the genes involved in the molecular mechanism behind the synergistic activity through gene expression studies in breast cancer cell line models. The proposed validation study will not only strengthen the reliability of our computational predictions but also provide a reliable basis for further experimental investigations. Ultimately, our work contributes to the development of effective combinatorial targeted therapies for BC, with the potential to improve patient outcomes and transform BC treatment strategies.

## Funding

Research reported was primarily supported by the National Cancer Institute (10.13039/100000054NCI) Cancer Center Support Grant P30 CA168524 and the Kansas Institute for Precision Medicine COBRE (Supported by the National Institute of General Medical Science award P20 GM130423) and the Kansas IDEA Network of Biomedical Research Excellence (P20 GM103418).

## CRediT authorship contribution statement

**Nanda Kumar Yellapu, Devin C Koestler, Jeffrey A Thompson:** Conception and design of study. **Nanda Kumar Yellapu:** acquisition of data. **Nanda Kumar Yellapu**, **Dong Pei, Emily Nissen:** analysis and/or interpretation of data. **Nanda Kumar Yellapu:** Drafting the manuscript. **Nanda Kumar Yellapu, Dong Pei, Emily Nissen, Devin C Koestler, Jeffrey A Thompson**: revising the manuscript critically for important intellectual content.

Approval of the version of the manuscript to be published (the names of all authors must be listed):

Nanda Kumar Yellapu, Dong Pei, Emily Nissen, Devin C Koestler, Jeffrey A Thompson.
