# Supplementary material for: Comprehensive exploration of JQ1 and GSK2801 targets in breast cancer using network pharmacology and molecular modeling approaches
Source: Comput Struct Biotechnol J. 2023 Jun 3;21:3224–33. doi: 10.1016/j.csbj.2023.06.003 (PMC10781883; doi:10.1016/j.csbj.2023.06.003)
Supplement: Supplementary file 7 — Supplementary material [file mmc7.docx]

**Table S1**. Gene ontology enrichment analysis of JQ1 and GSK2801 BC targets. The top GO terms are represented as bar graphs rank-order according to their adjusted p-values. The X-axis indicates the number of genes and Y-axis GO terms.

|  | **JQ1** | **GSK2801** |
| --- | --- | --- |
| **Biological Process** | 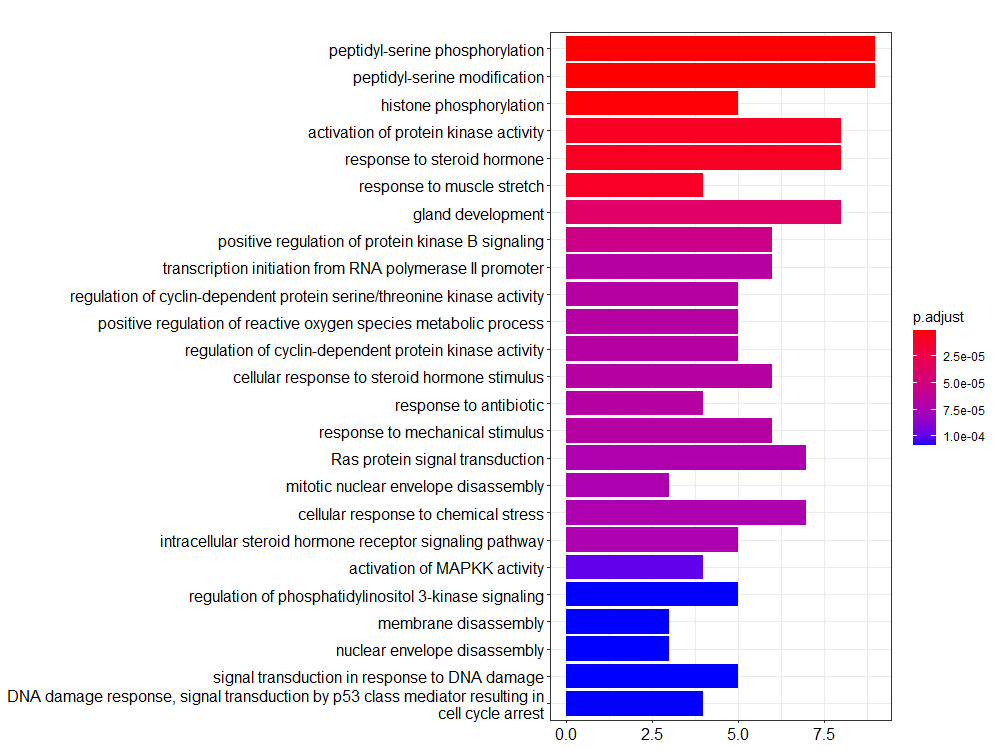 | 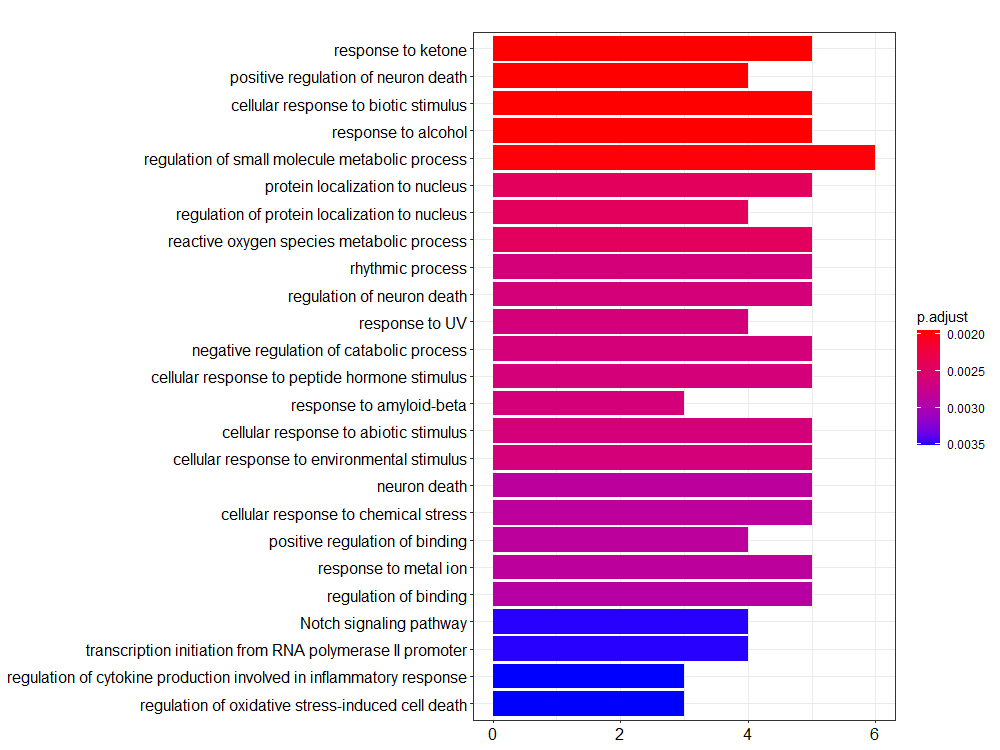 |
| **Molecular Function** | 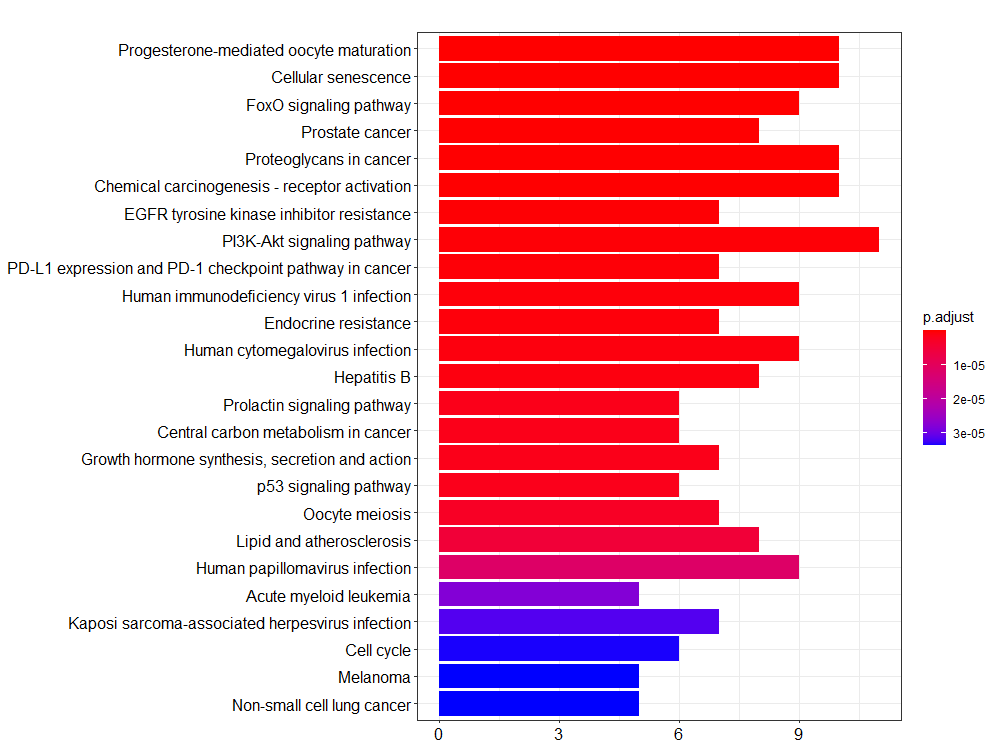 | 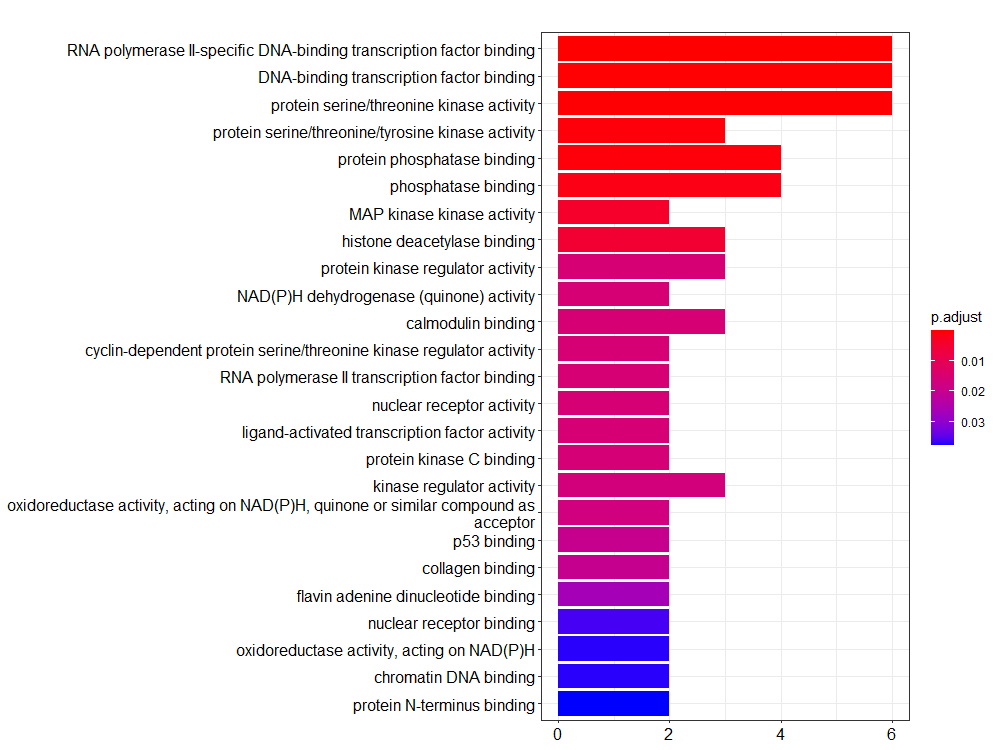 |
| **Metabolic Pathways** | 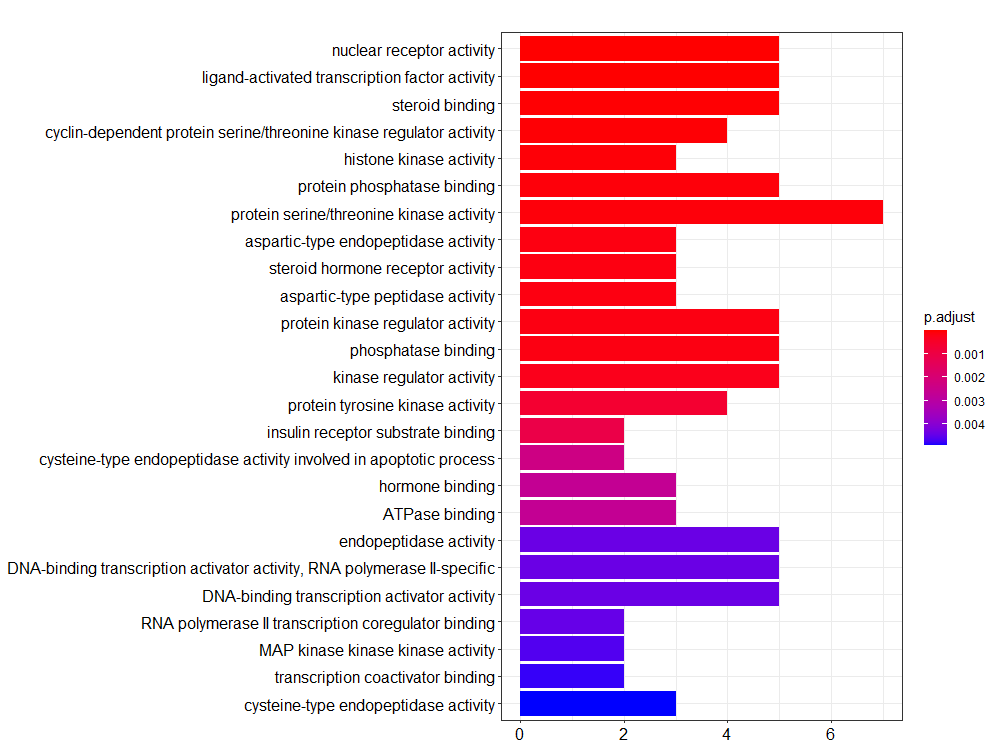 | 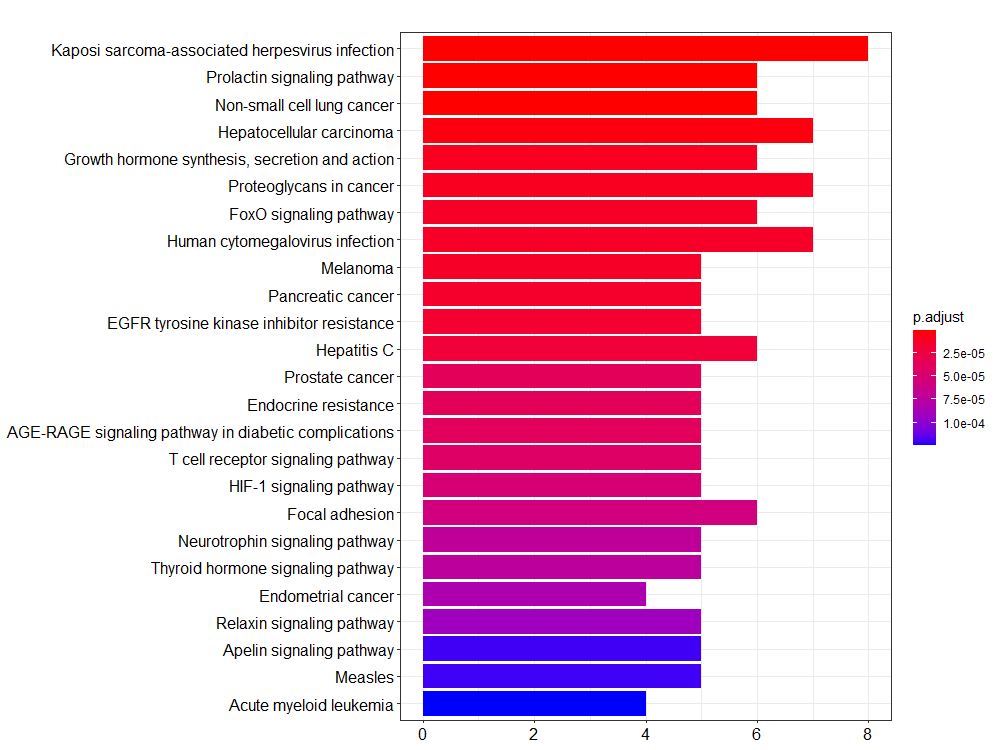 |

**Table S2**. Molecular docking of JQ1 against its BC targets. The ligand-protein docking complexes are shown in this table. JQ1 is represented as stick models and the target proteins as cartoon models. JQ1 is observed to bind in the binding site of target proteins.

| **Protein** | **Docking Score (kcal/mol)** | **Protein-Ligand complex** | **Binding site interactions** |
| --- | --- | --- | --- |
| **AR** | -7.61 | 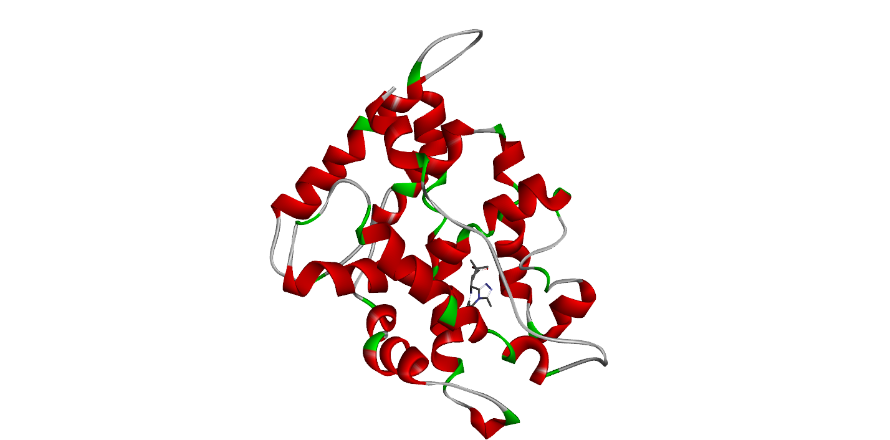 | 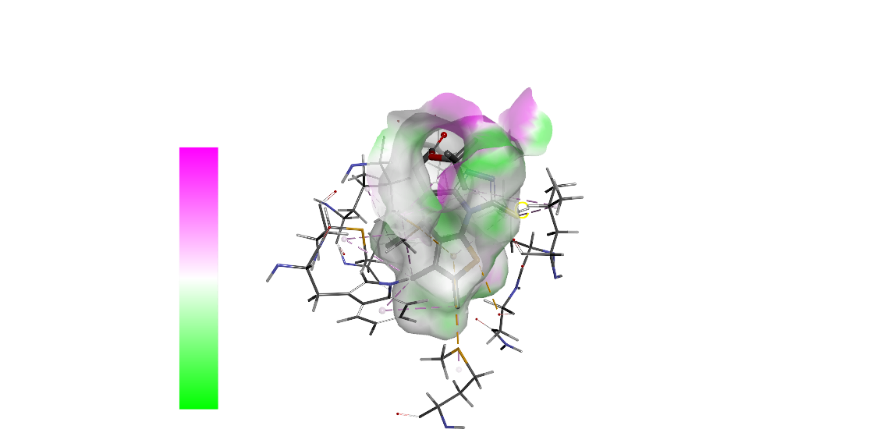 |
| **CASP3** | -7.65 | 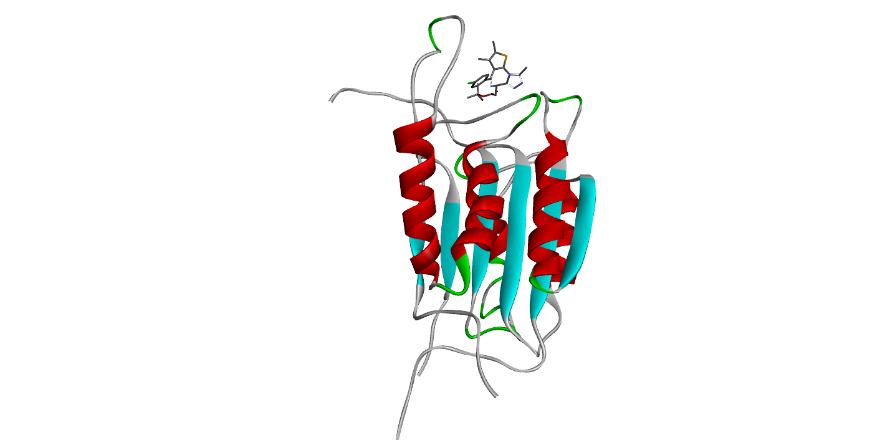 | 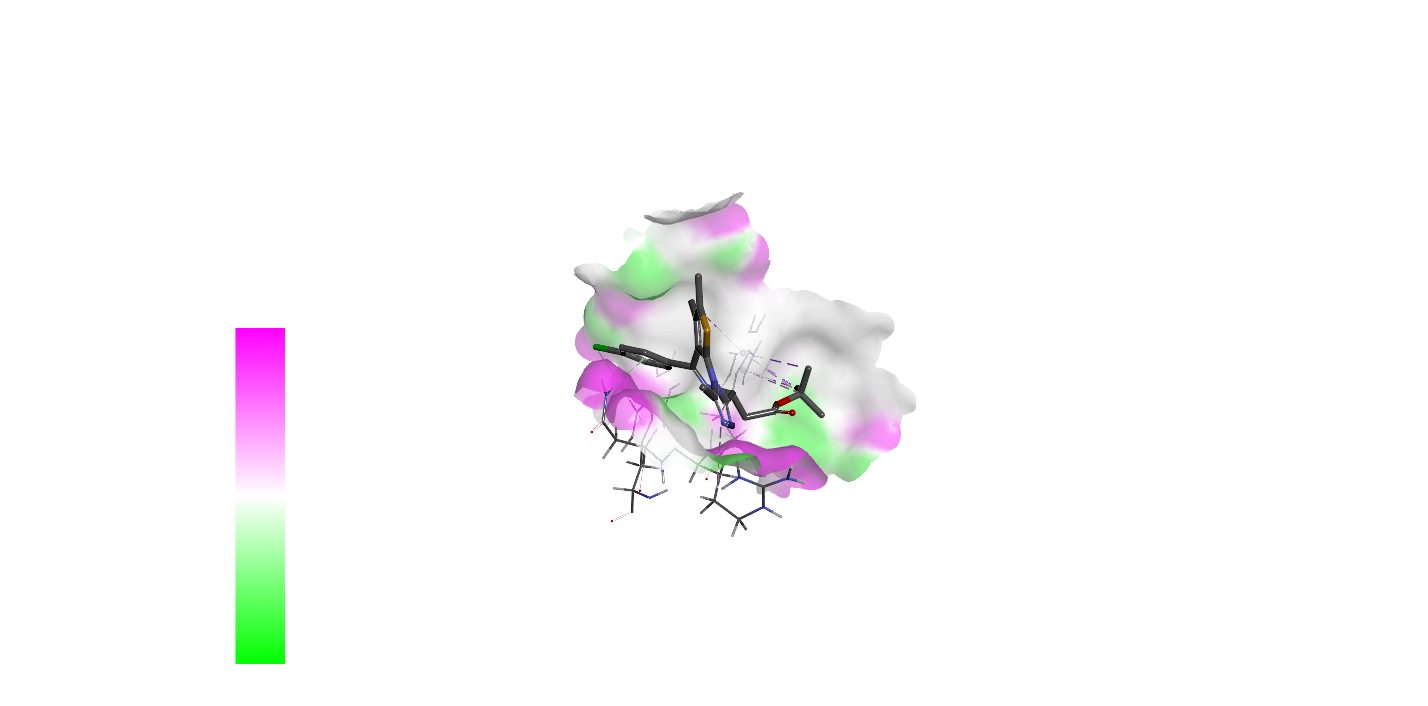 |
| **CASP7** | -6.08 | 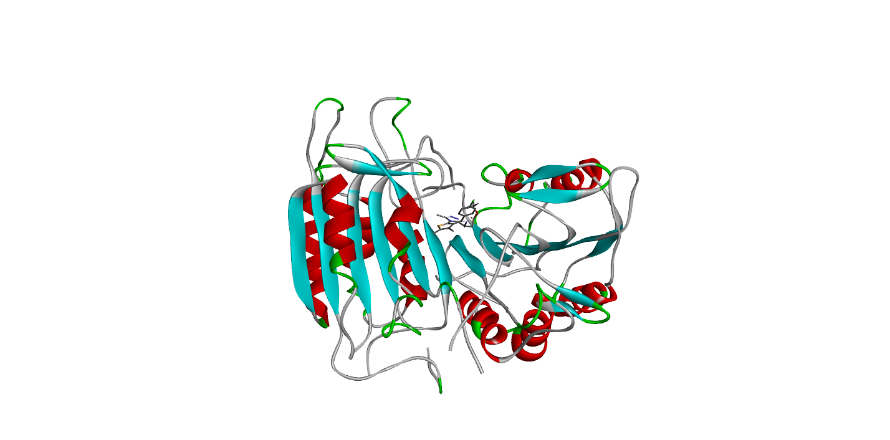 | 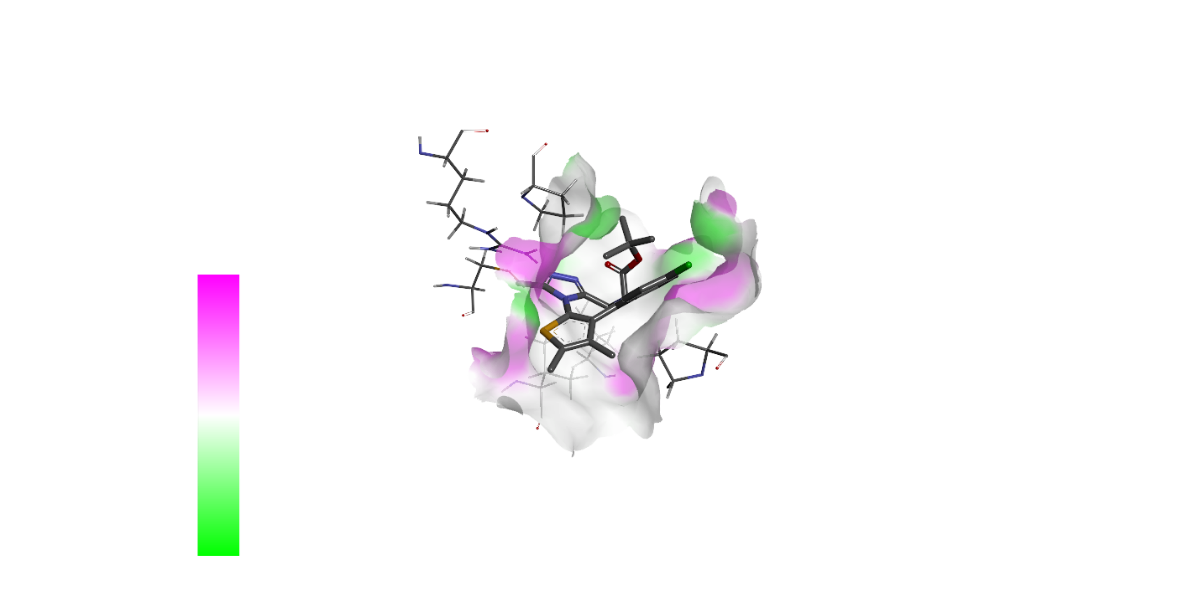 |
| **CCNA2** | -7.37 | 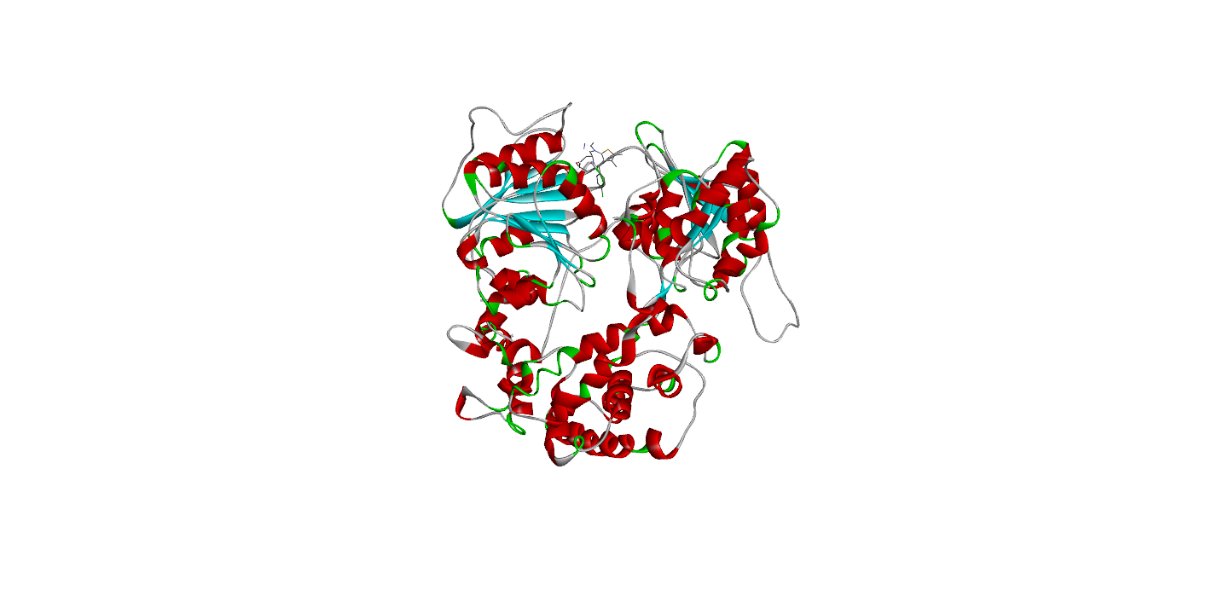 | 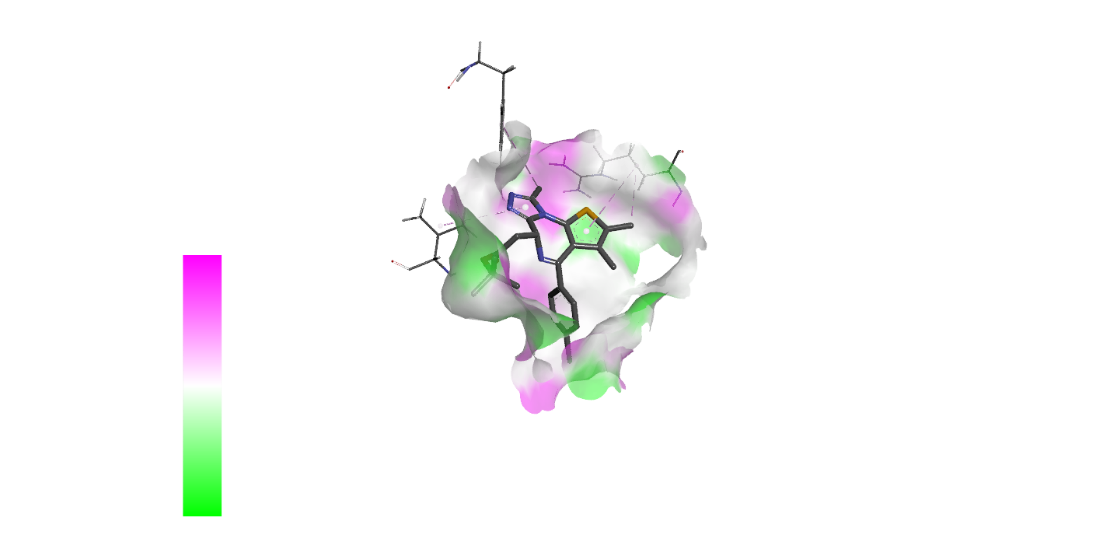 |
| **CCNB1** | -5.25 | 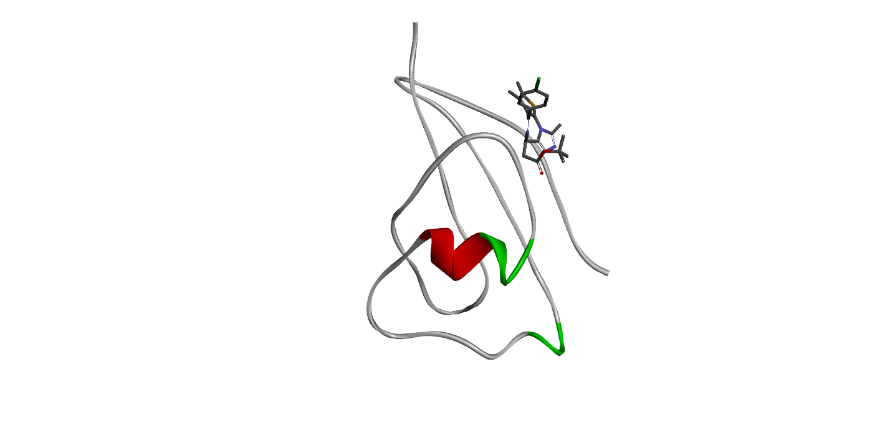 | 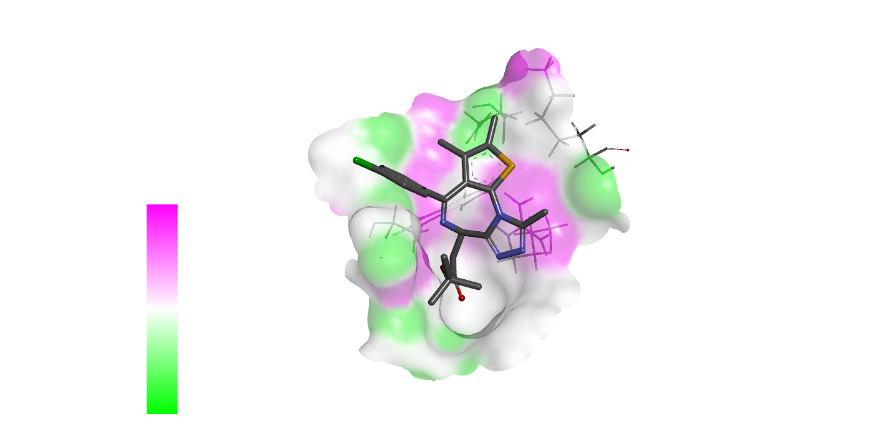 |
| **CCNB2** | -4.85 | 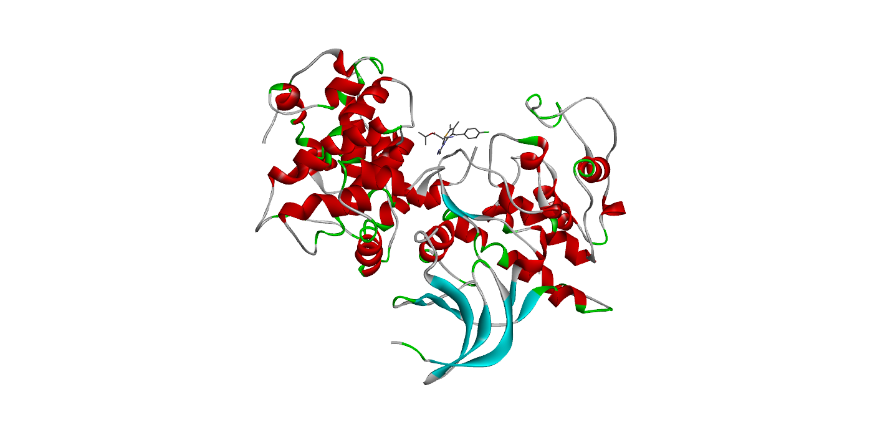 | 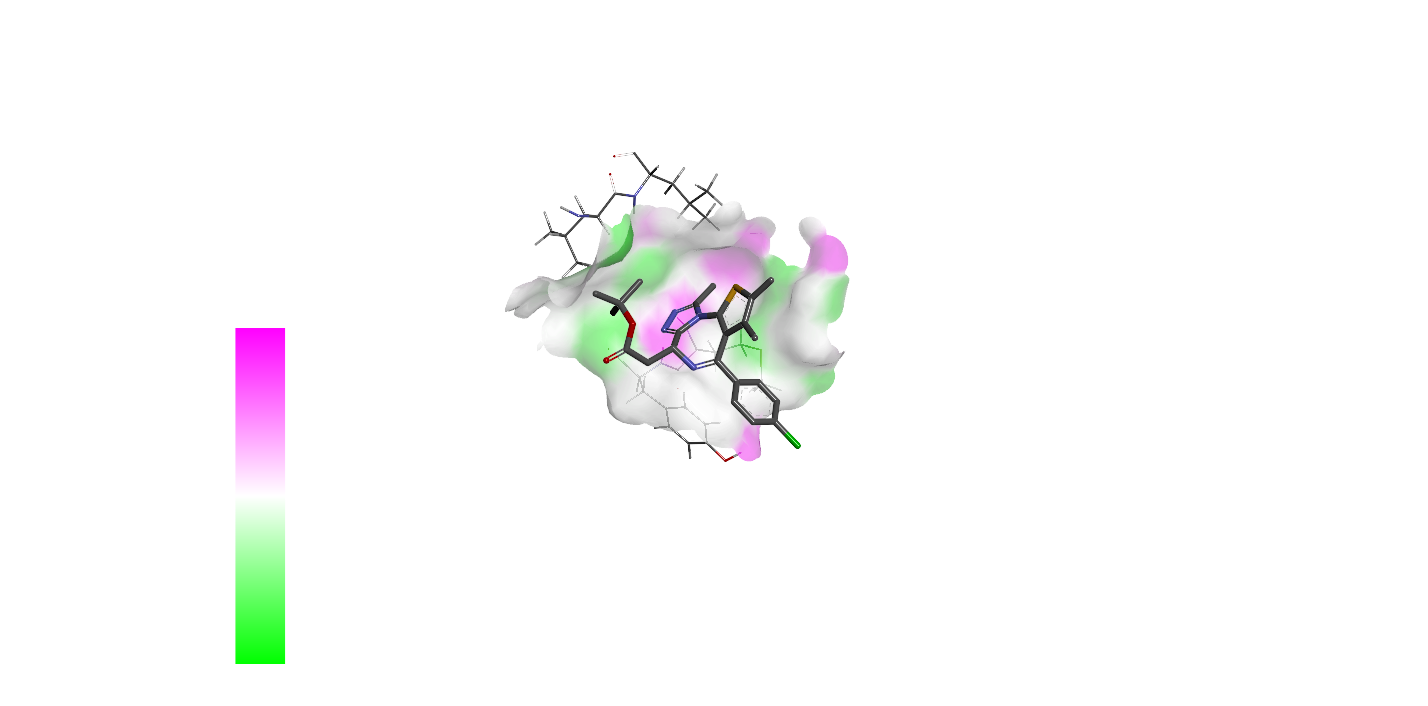 |
| **CDK1** | -5.75 | 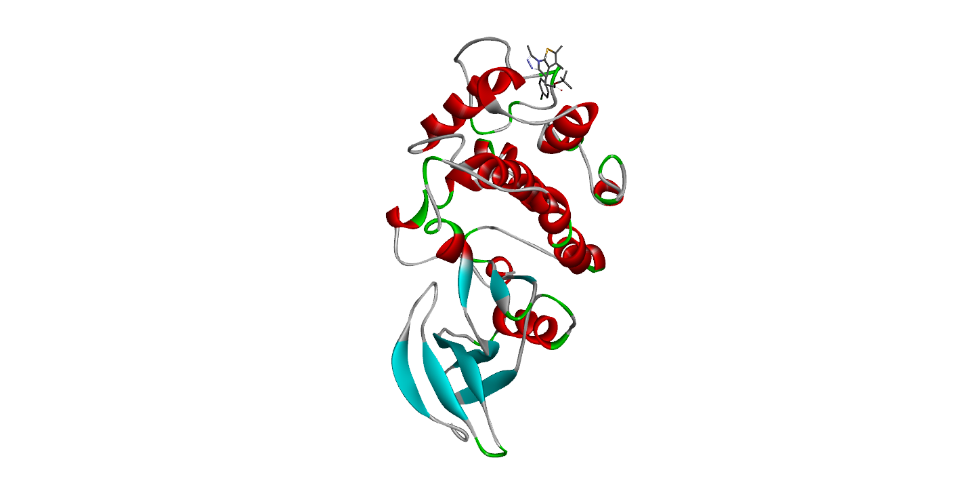 | 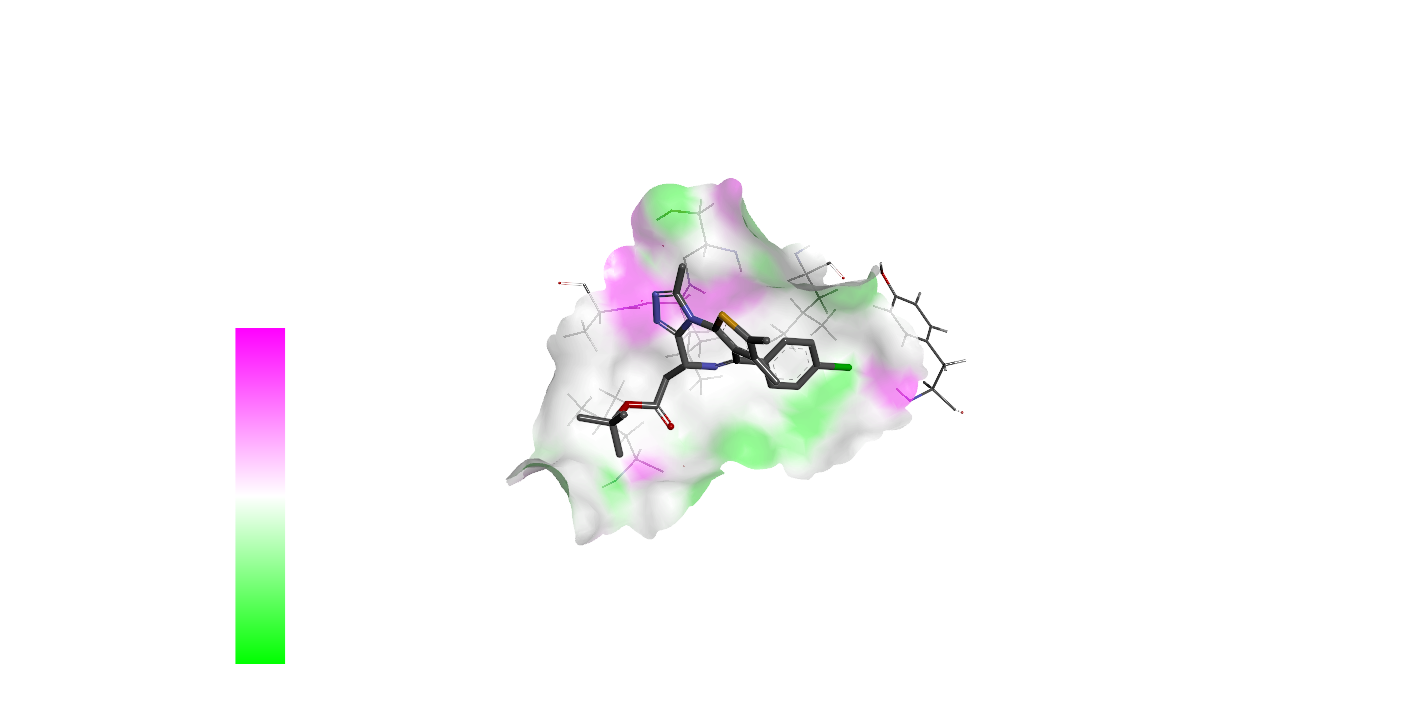 |
| **CDK2** | -7.54 | 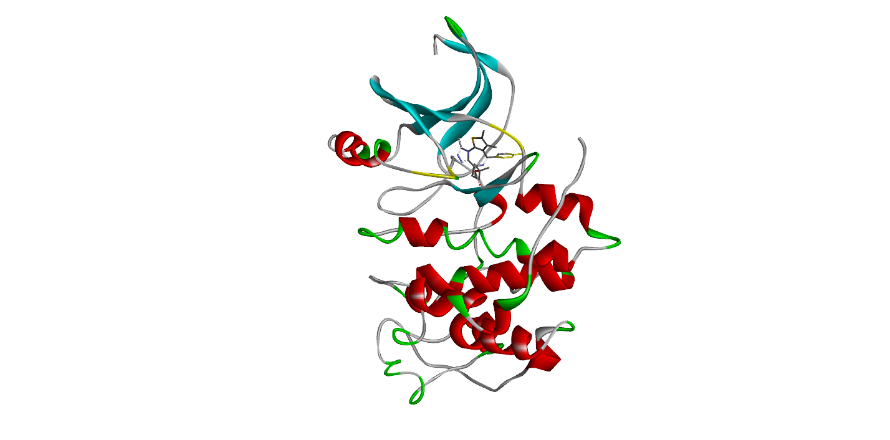 | 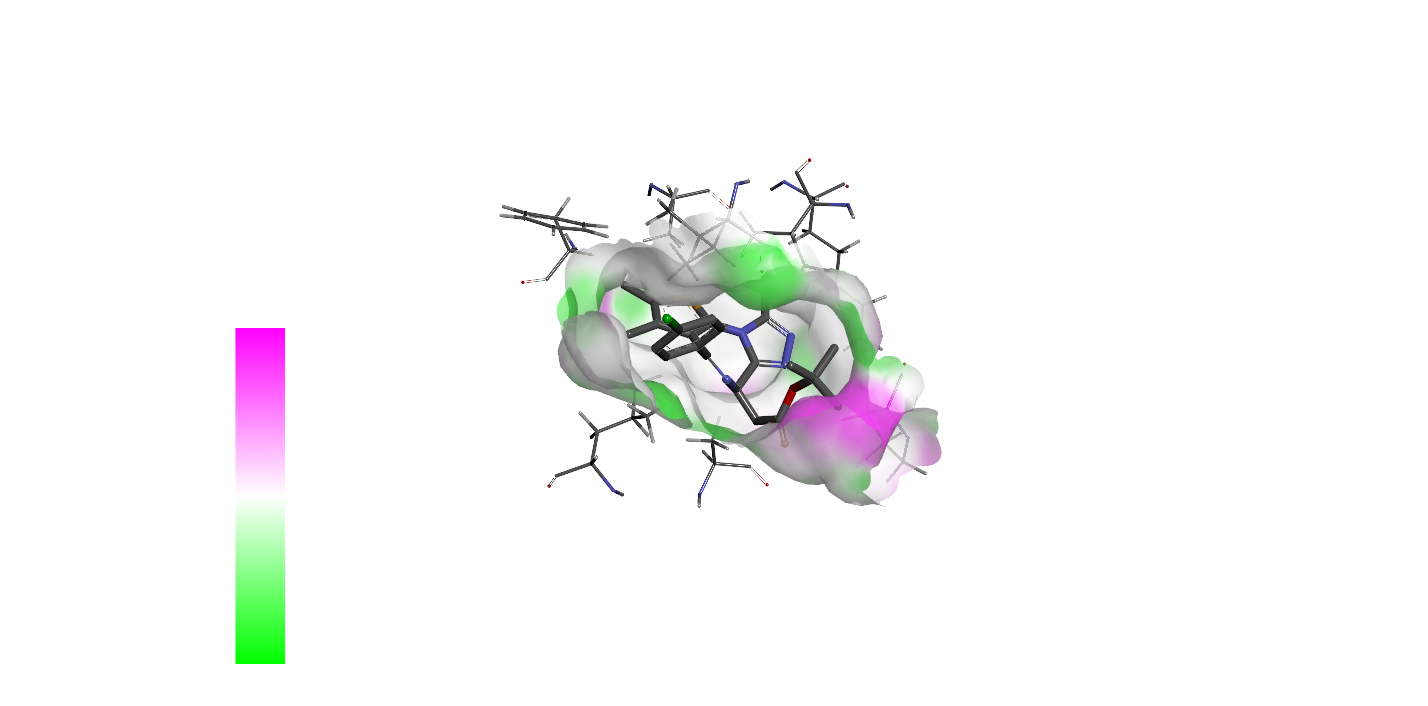 |
| **CTSD** | -6.11 | 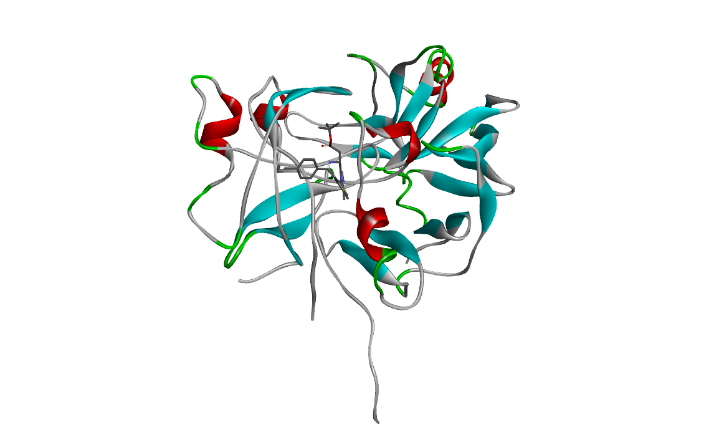 | 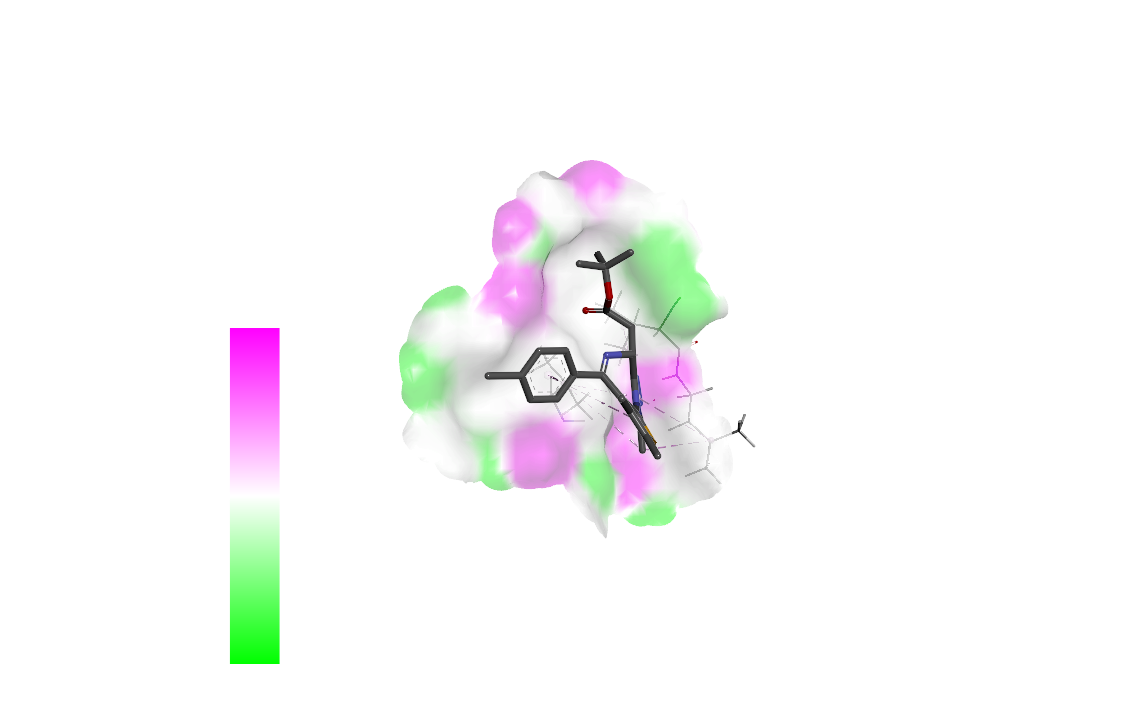 |
| **CYP17A1** | -7.17 | 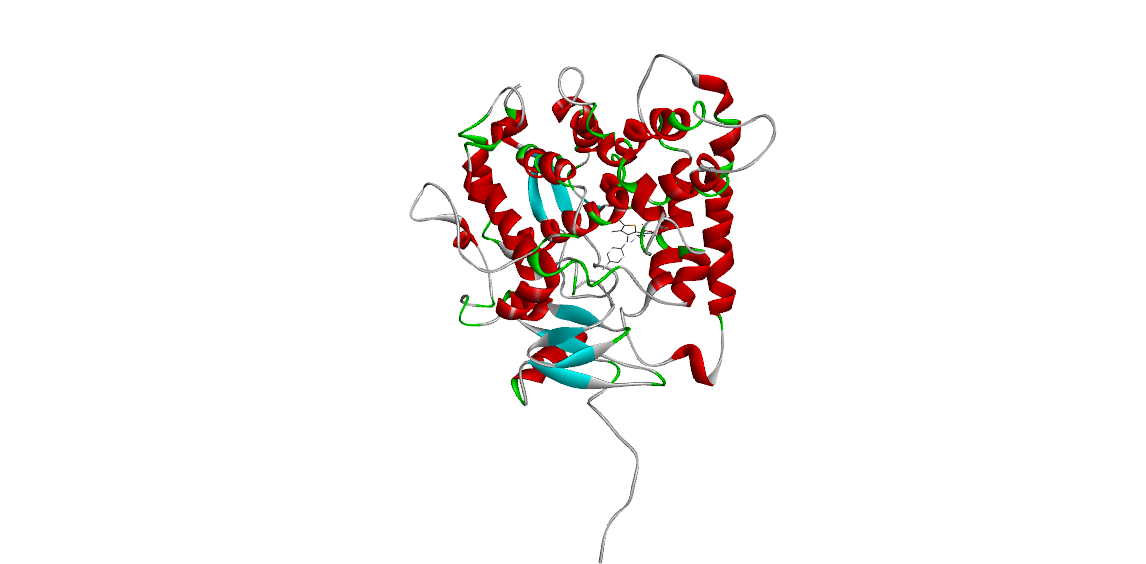 | 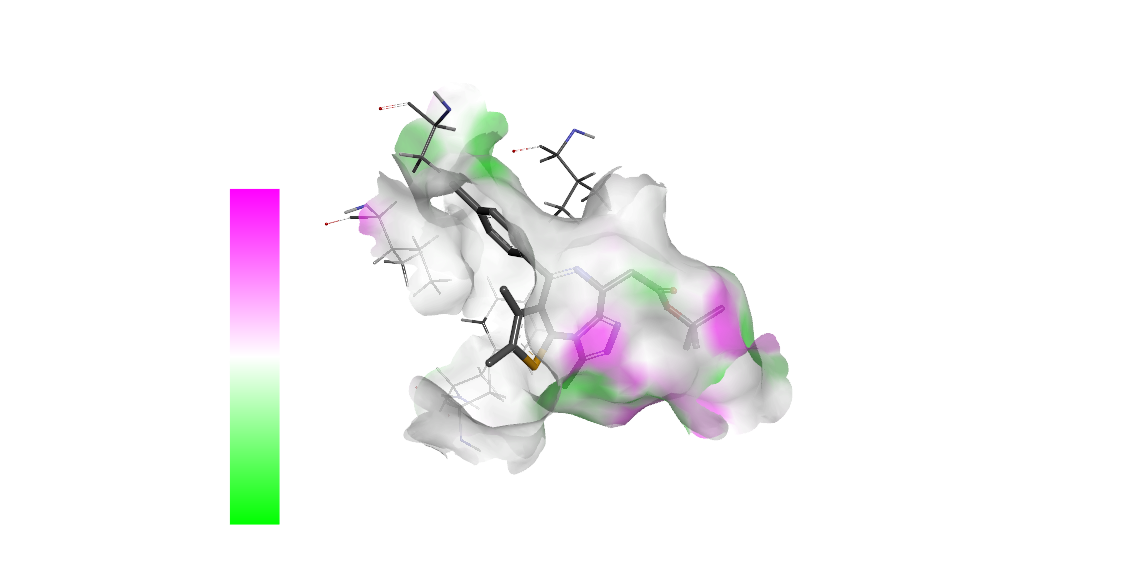 |
| **CYP3A4** | -7.38 | 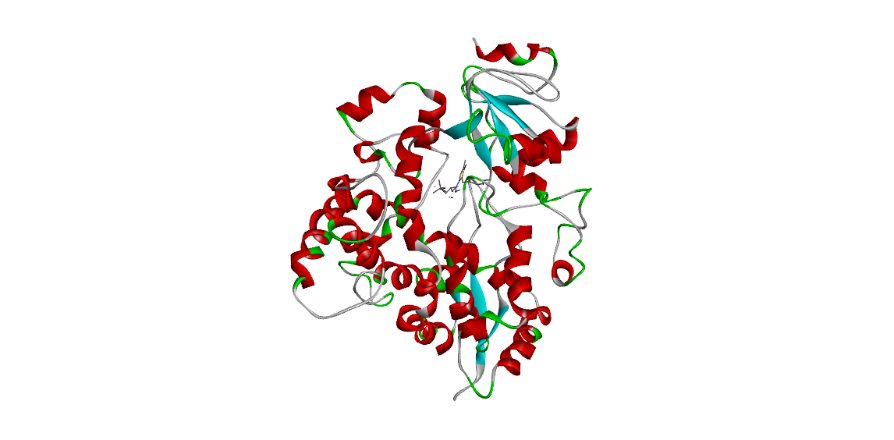 | 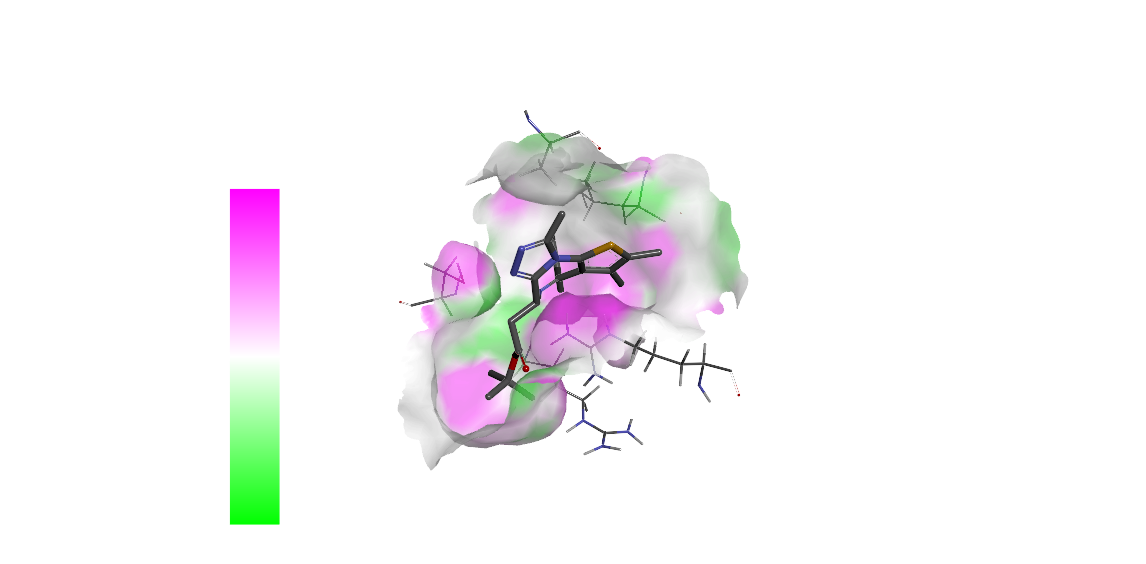 |
| **EGFR** | -5.18 | 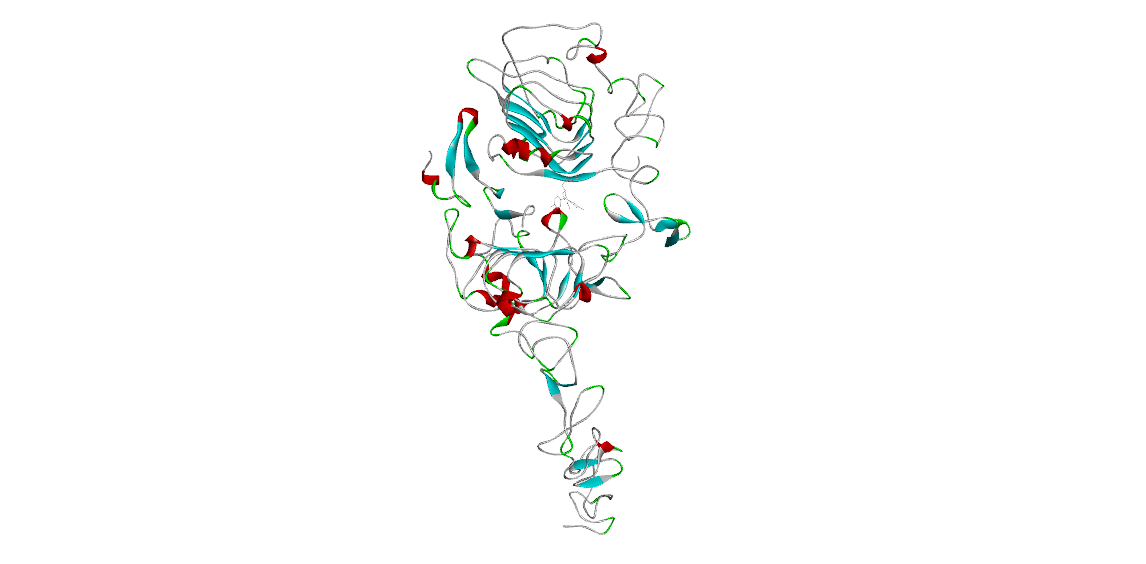 | 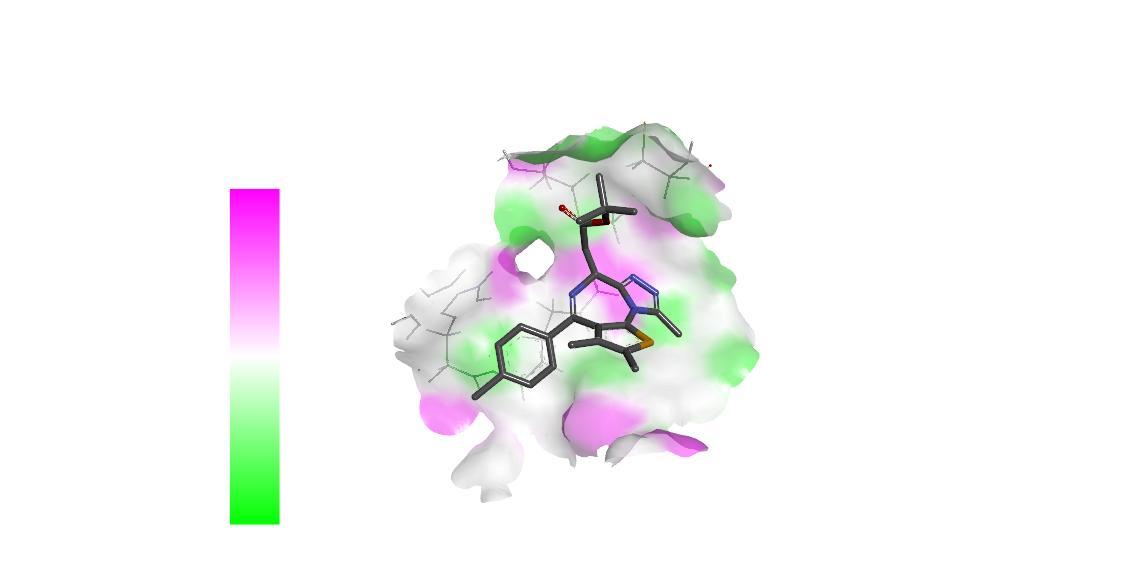 |
| **F2R** | -5.85 | 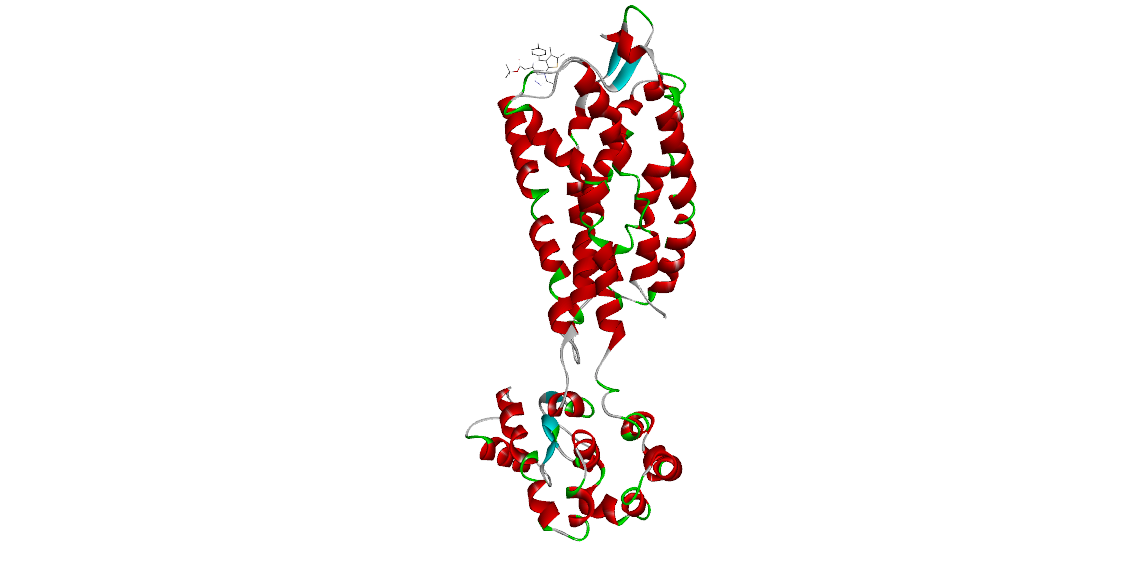 | 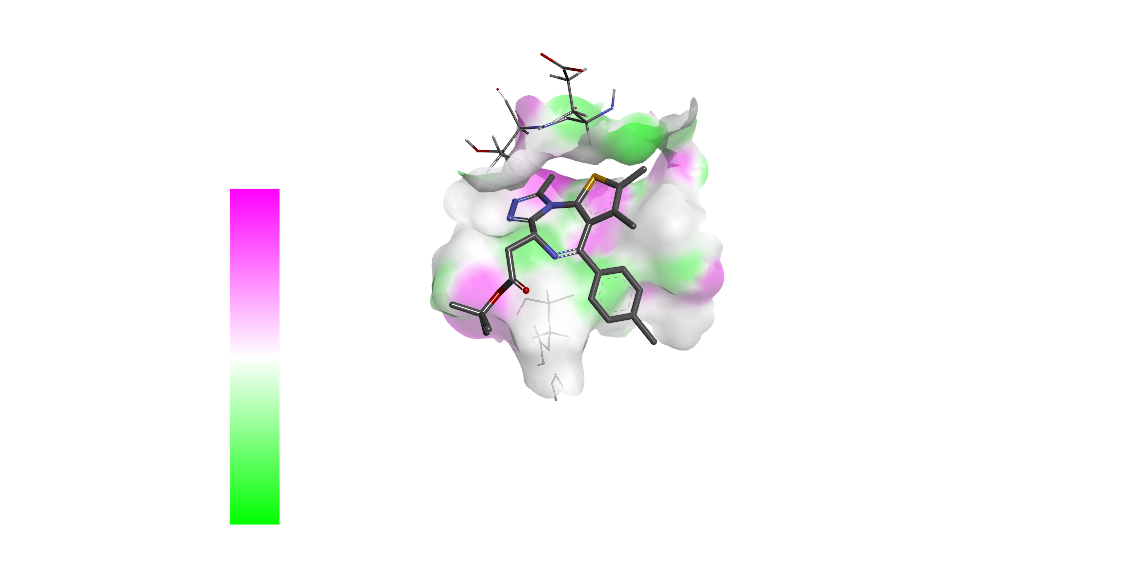 |
| **HSP90AA1** | -7.98 | 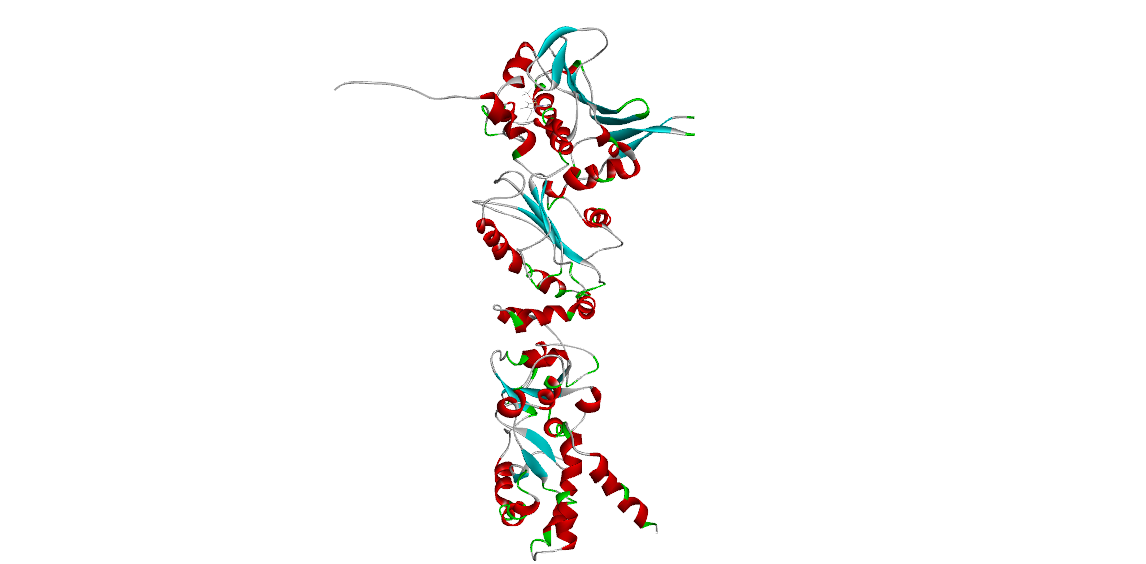 | 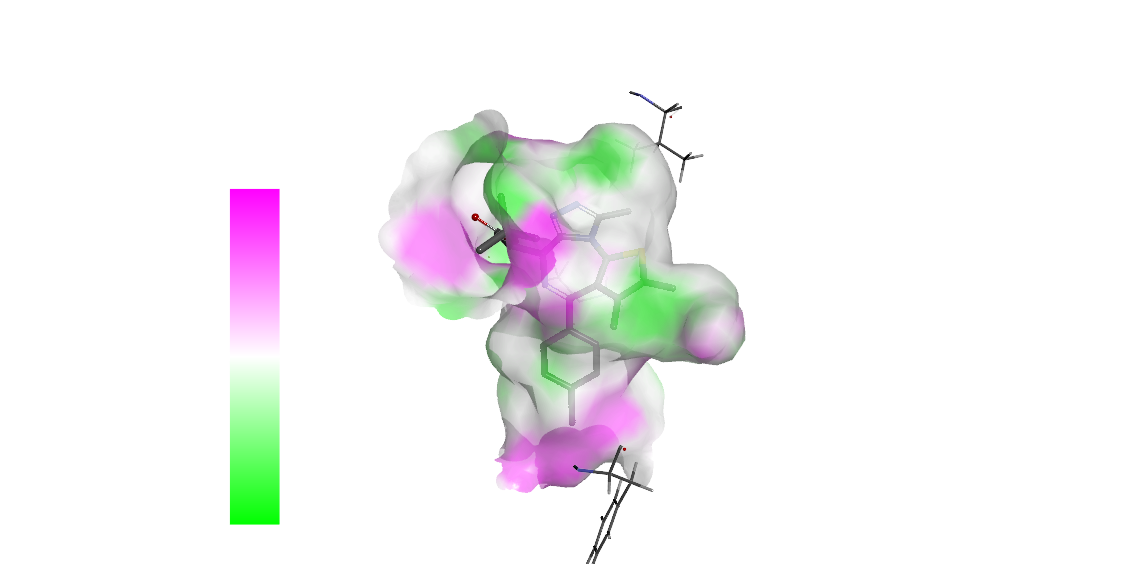 |
| **IDH1** | -4.46 | 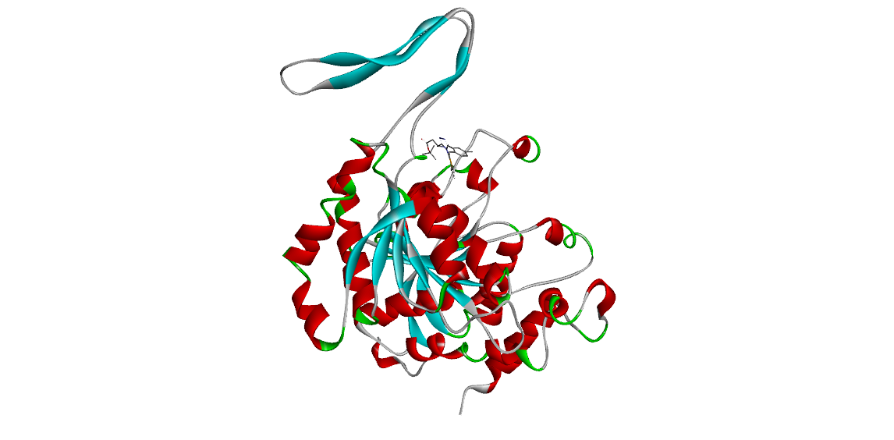 | 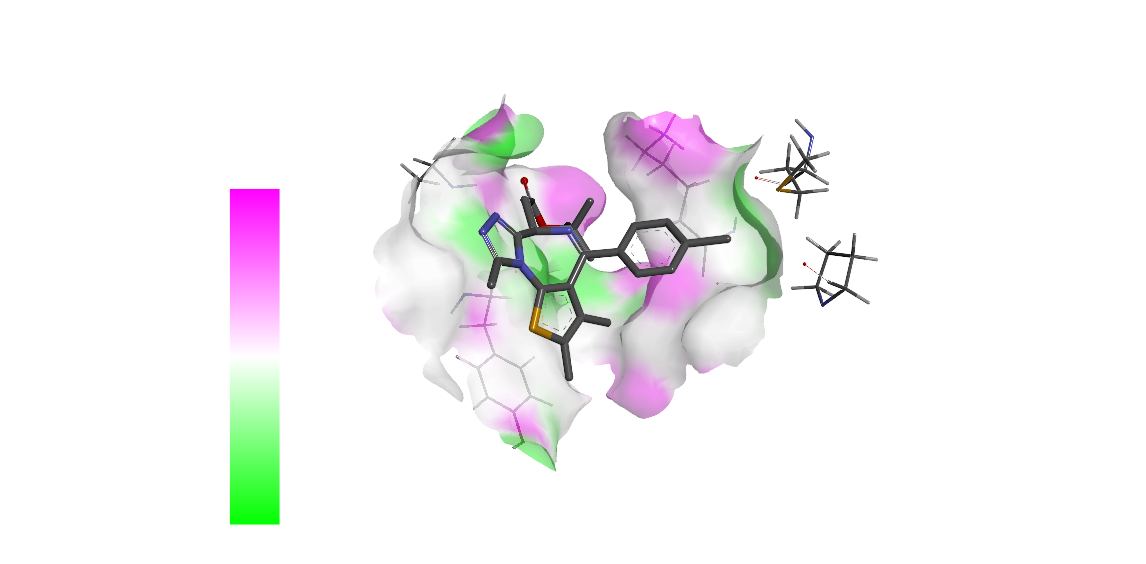 |
| **JAK2** | -5.88 | 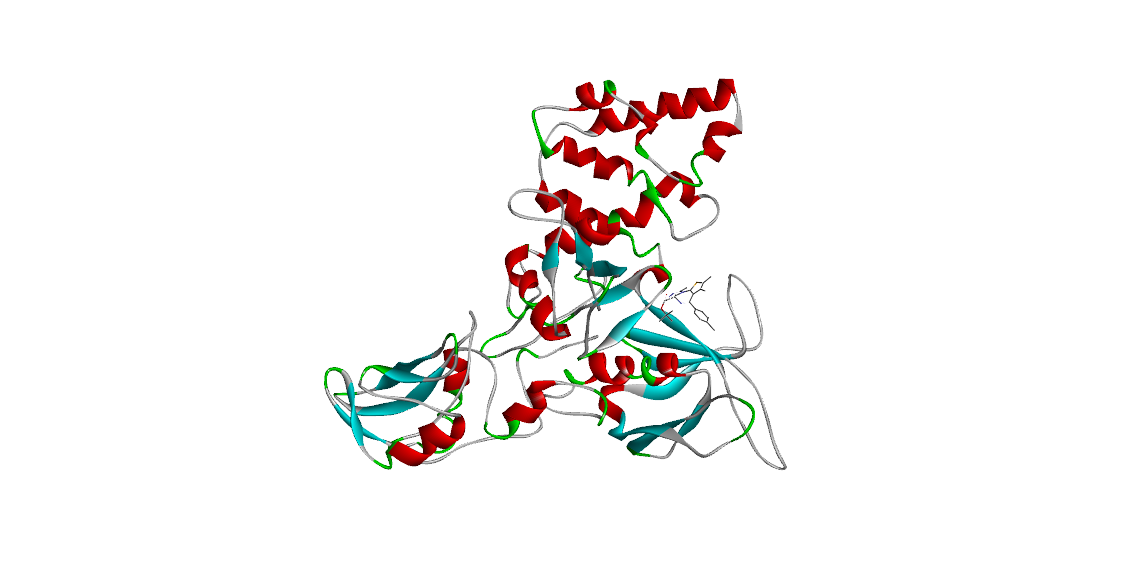 | 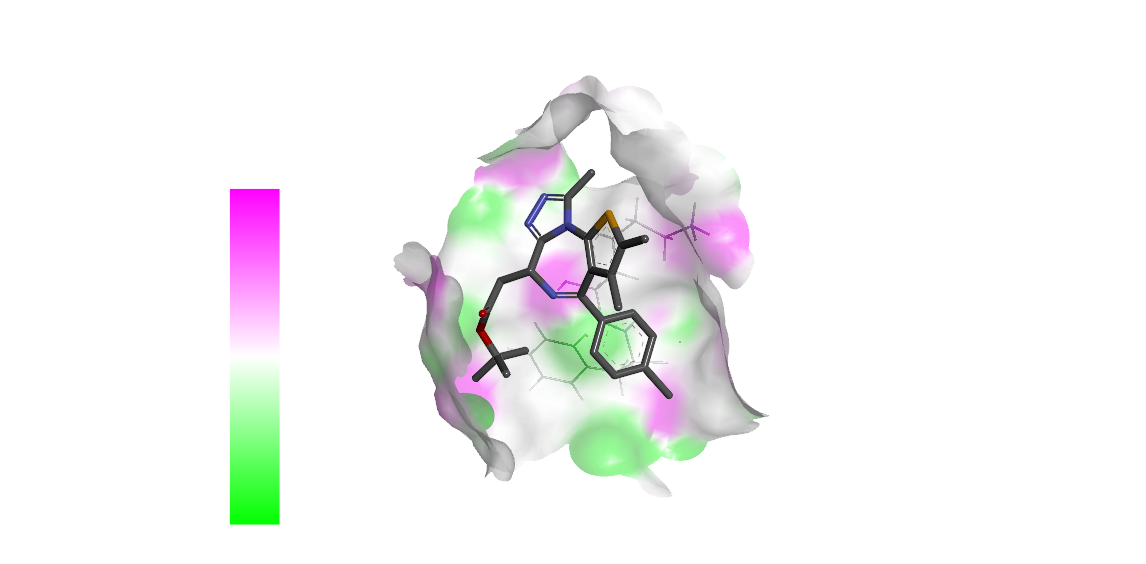 |
| **MDM2** | -6.43 | 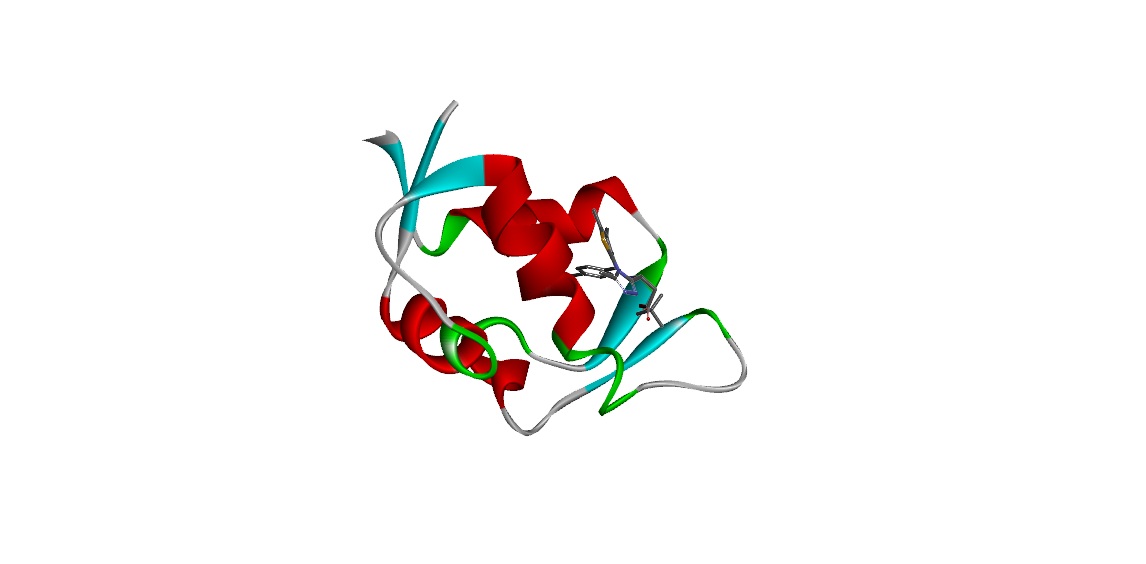 | 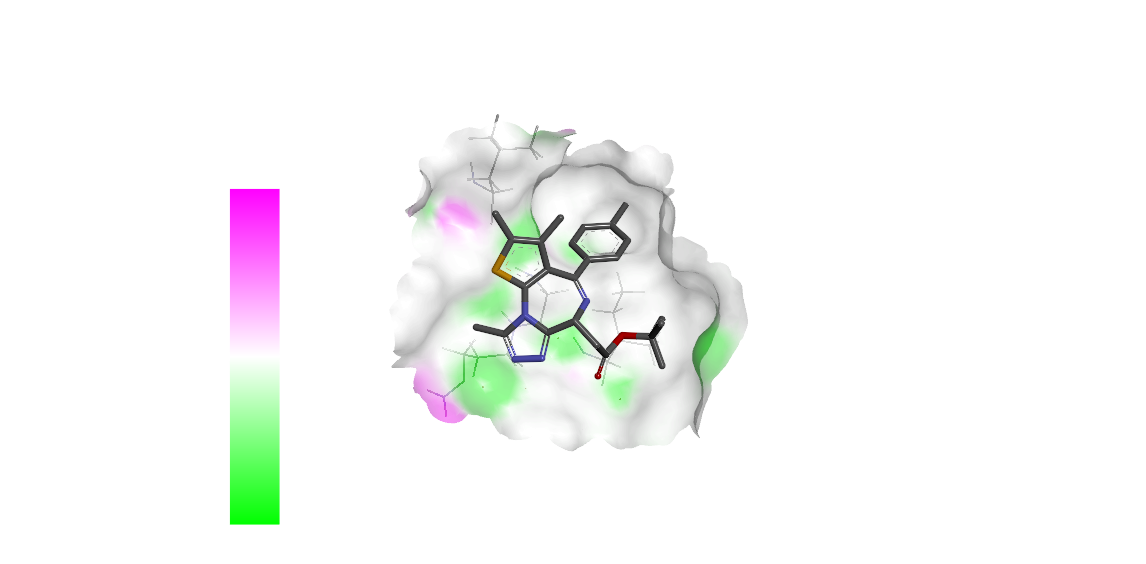 |
| **MTOR** | -3.96 | 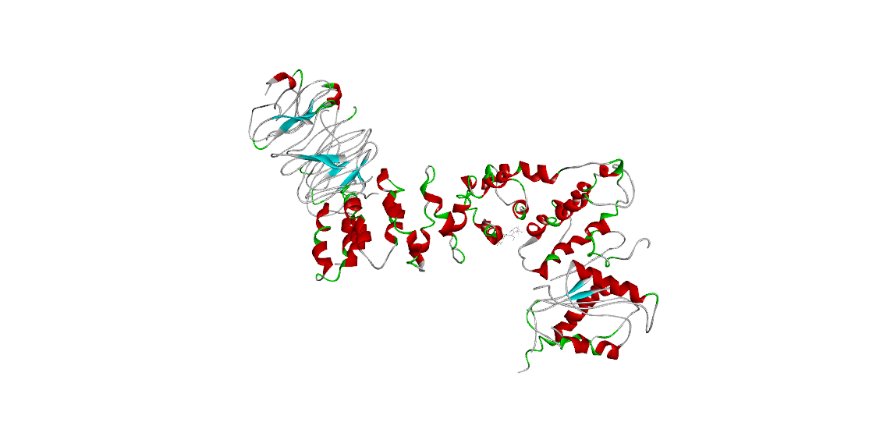 | 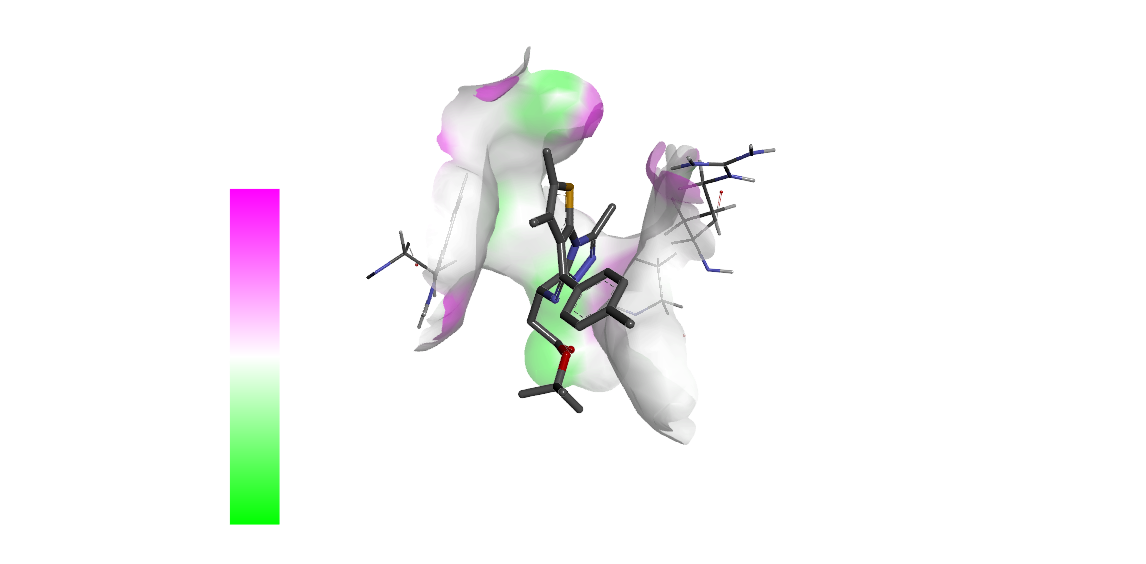 |
| **NR3C1** | -5.75 | 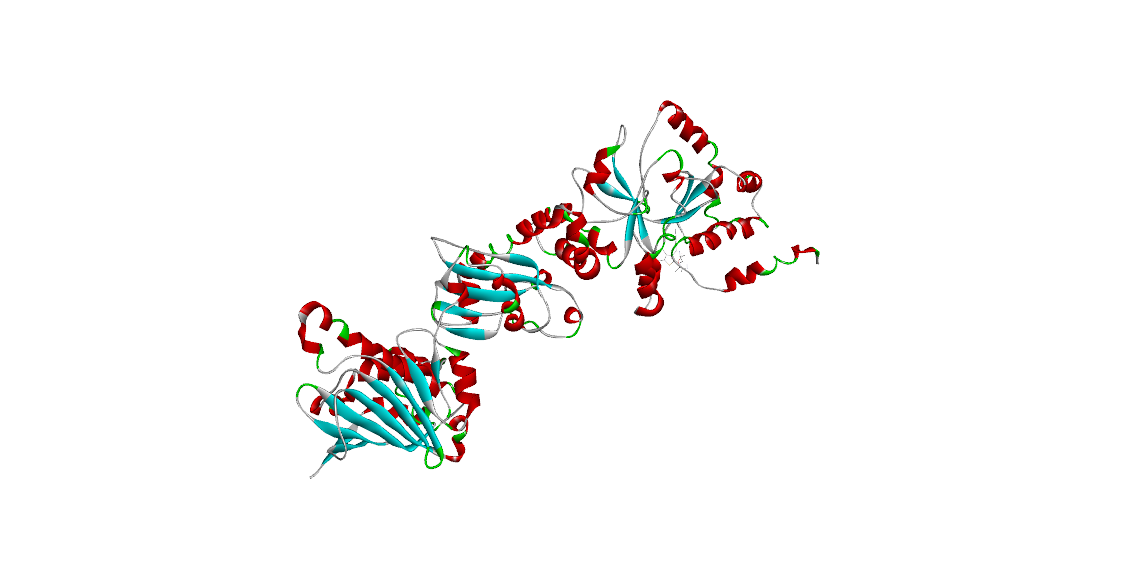 | 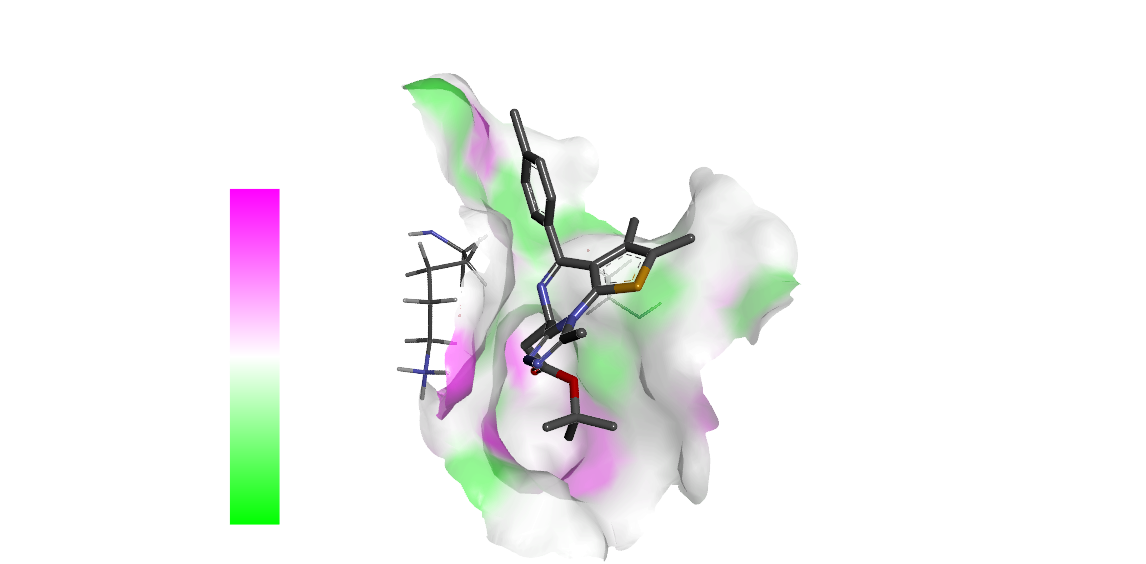 |
| **NR3C2** | -6.91 | 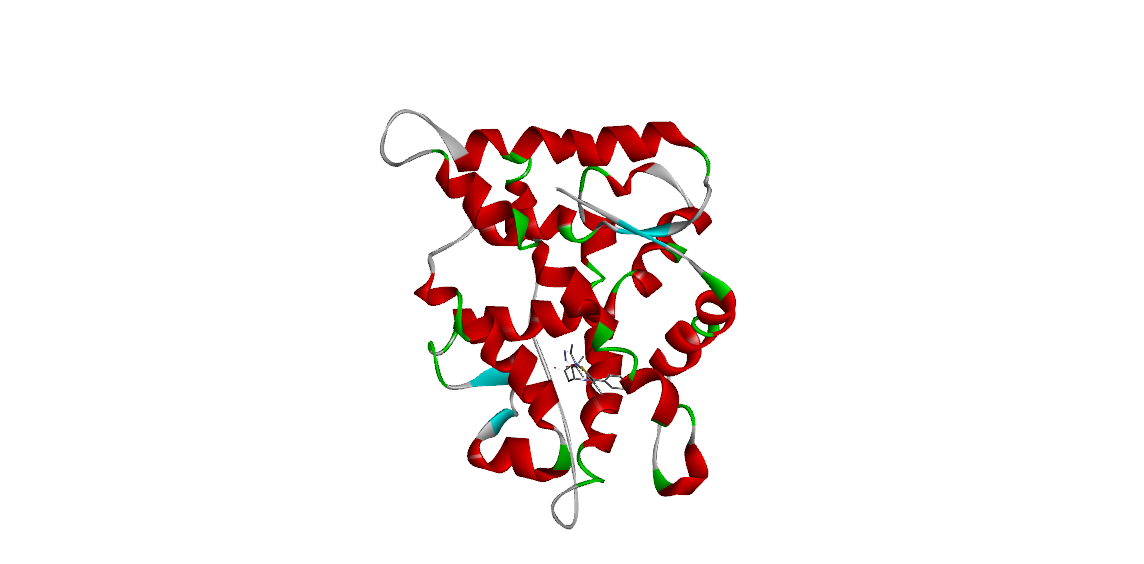 | 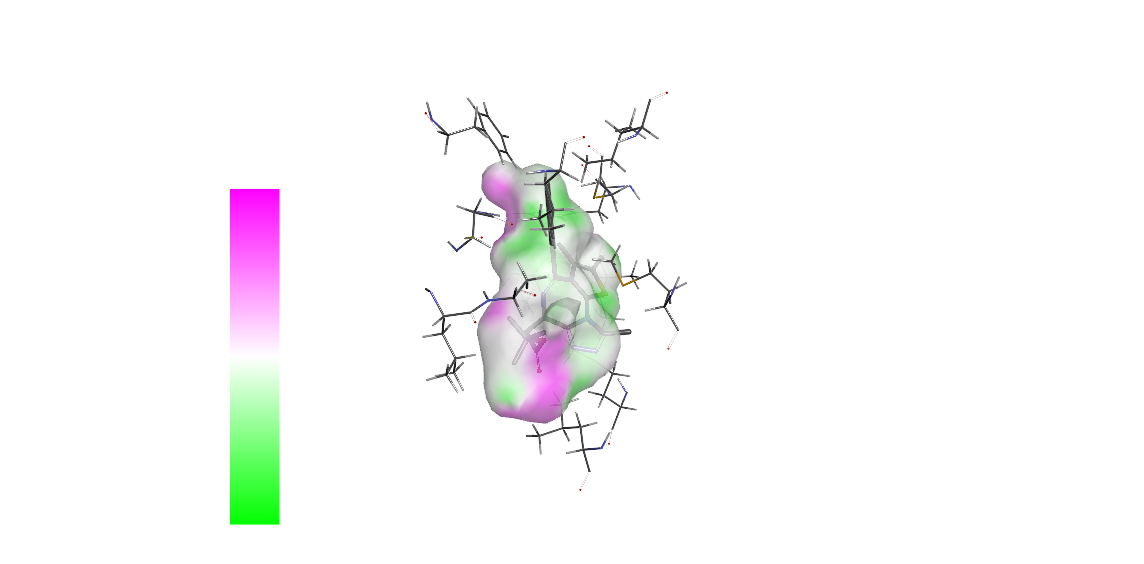 |
| **PGR** | -6.16 | 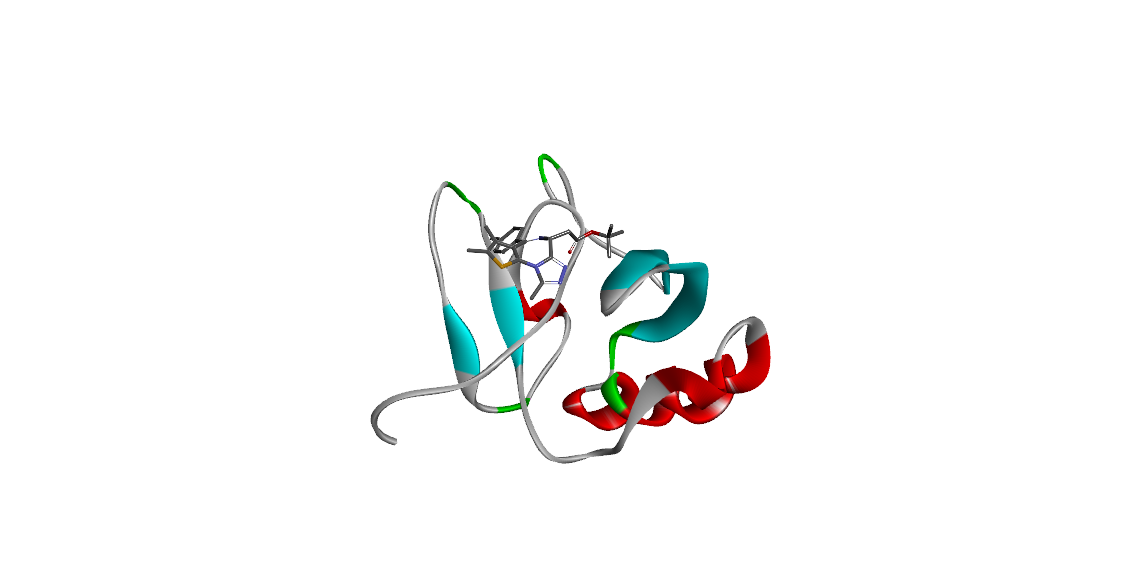 | 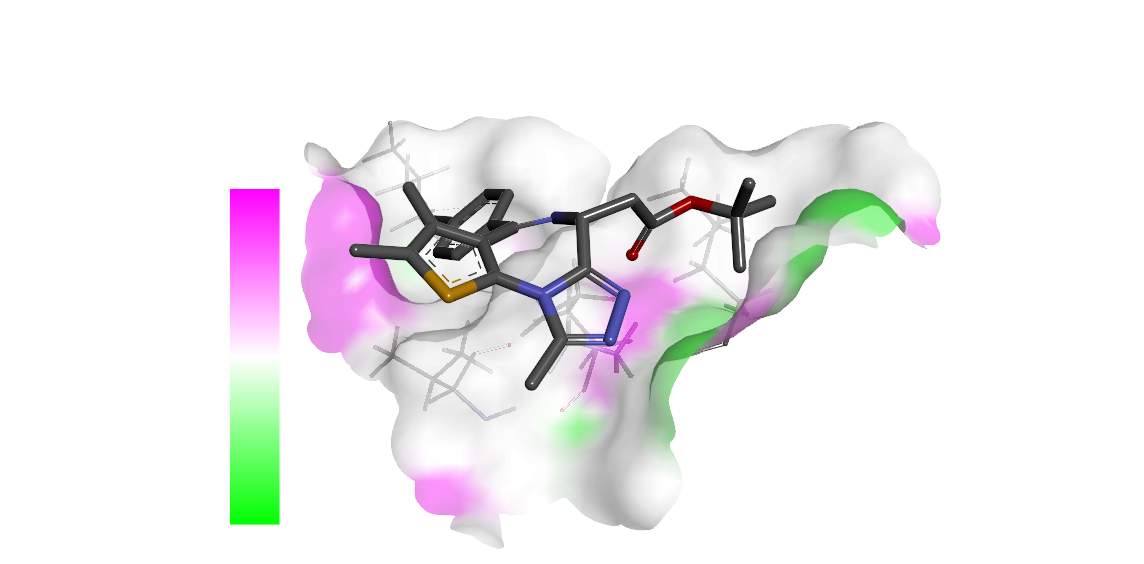 |
| **PIK3CA** | -8.27 | 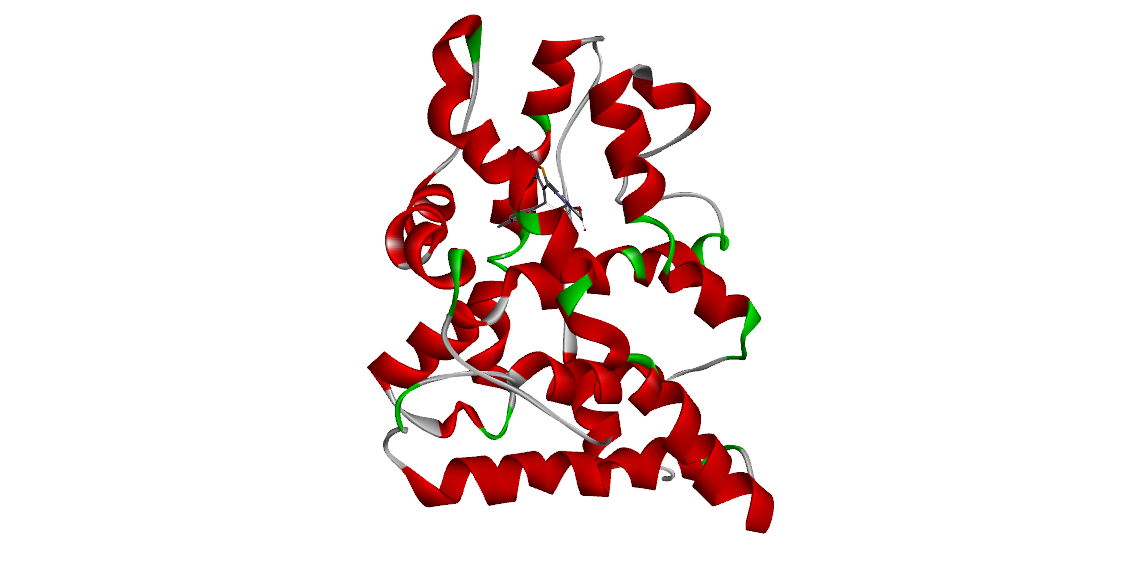 | 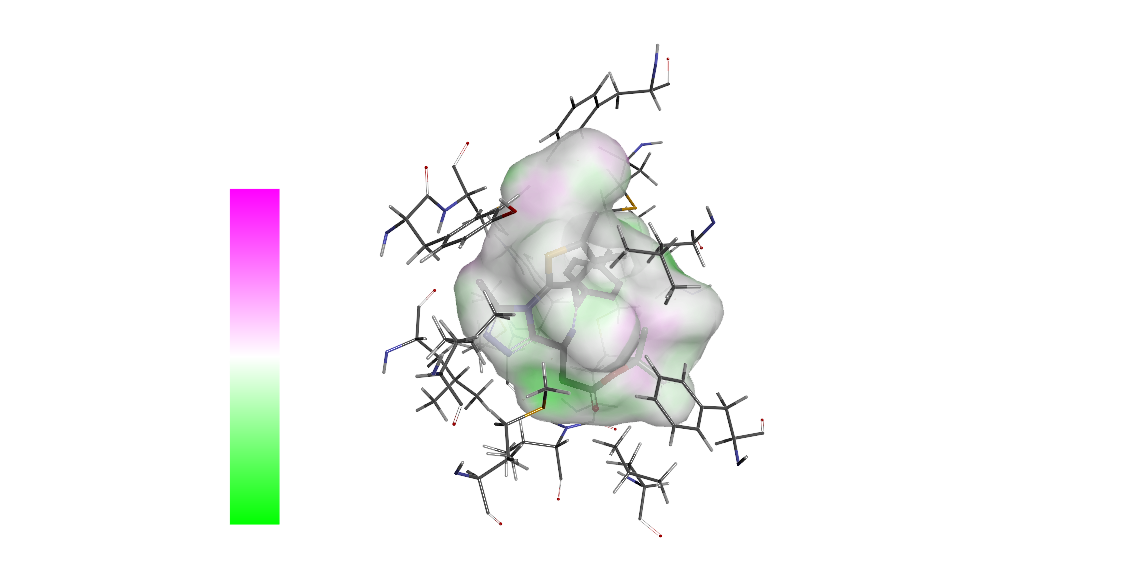 |
| **PTK2** | -4.7 | 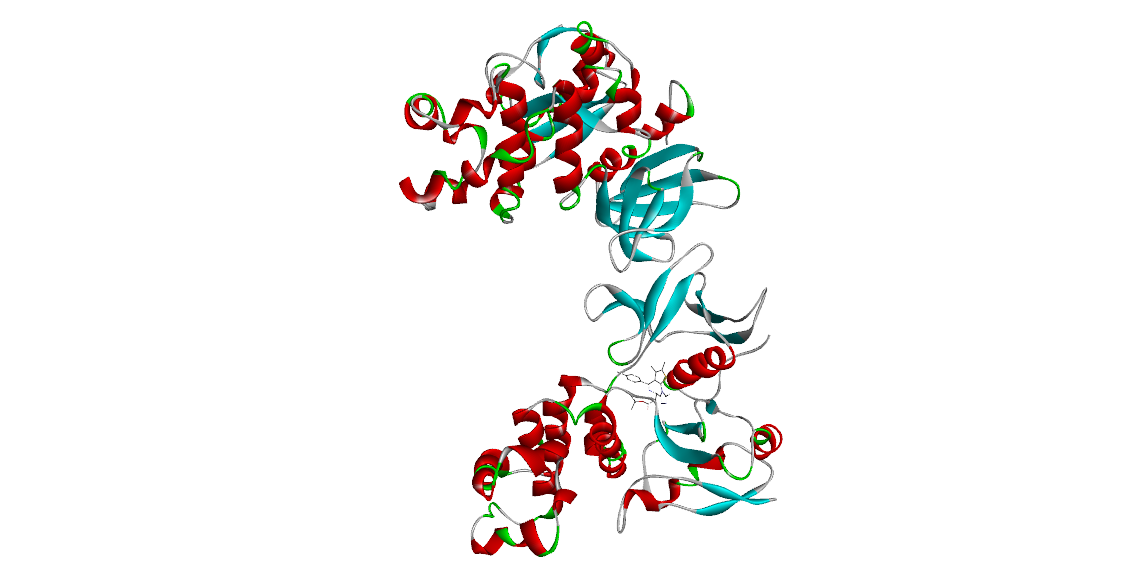 | 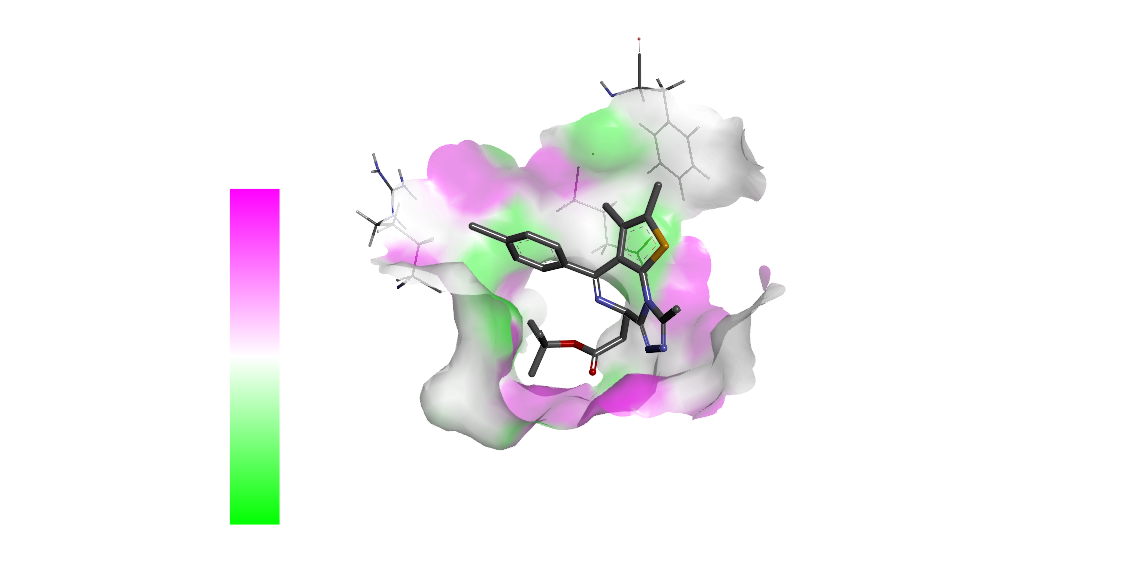 |
| **RAF1** | -6.23 | 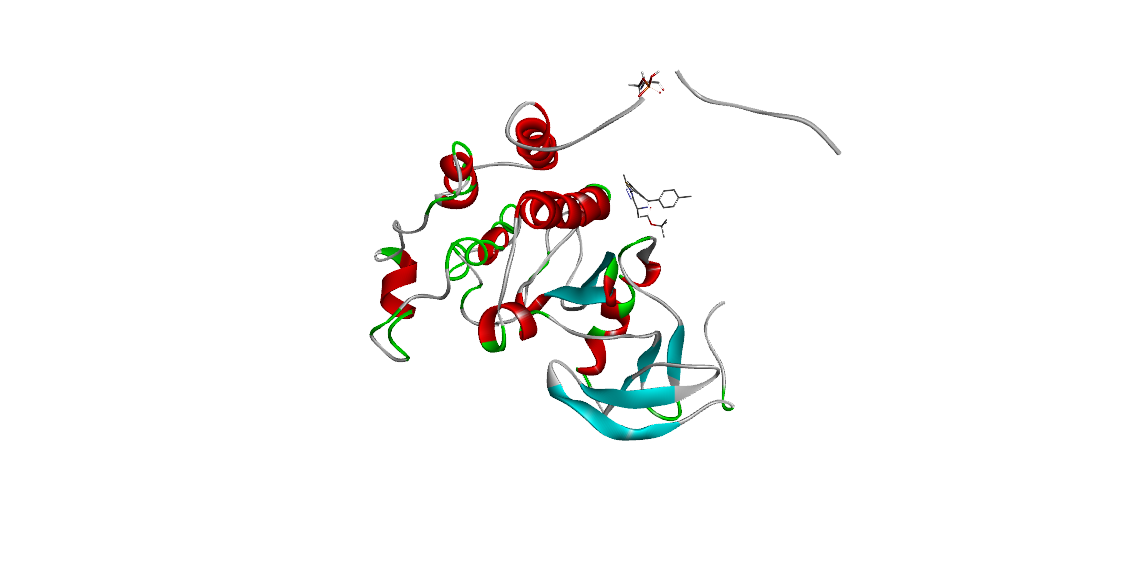 | 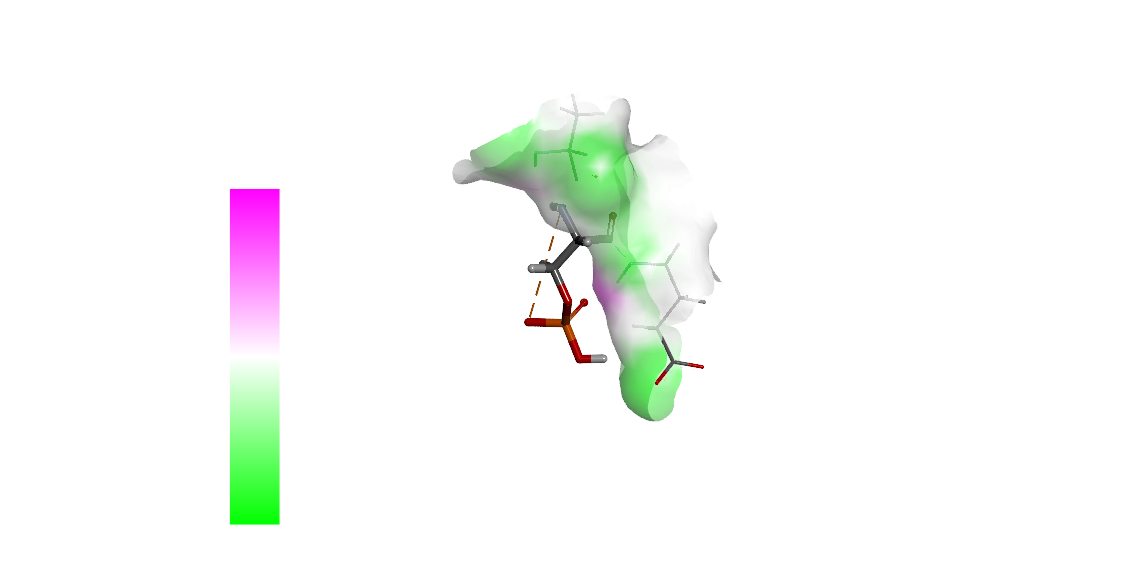 |
| **CTSB** | -3.58 | 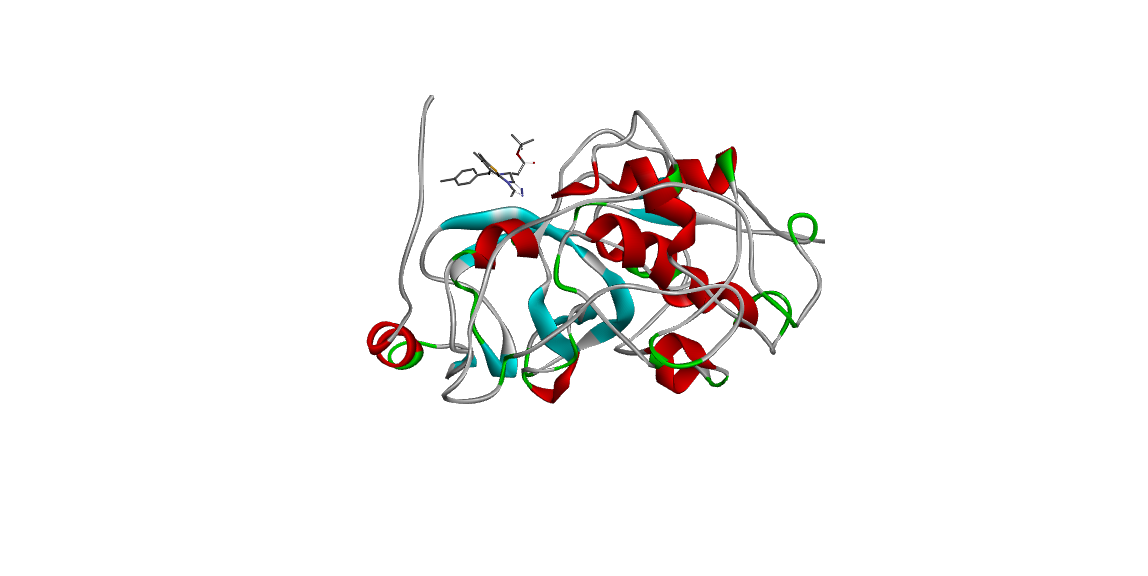 | 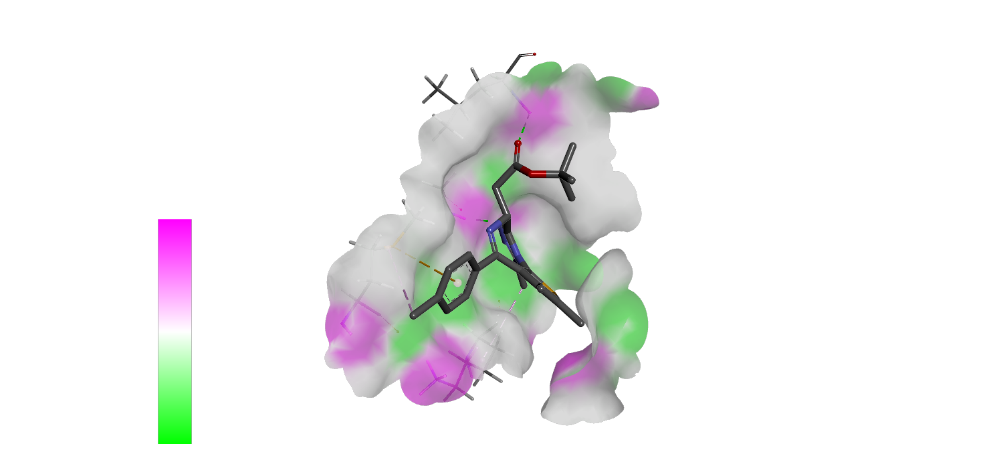 |
| **MAPK14** | -7.53 | 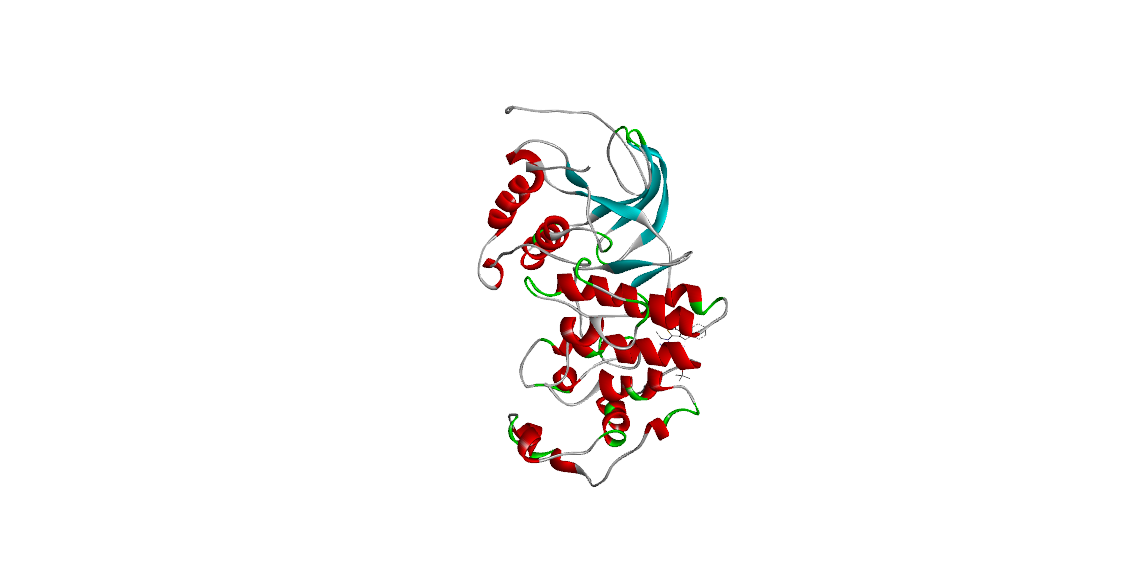 | 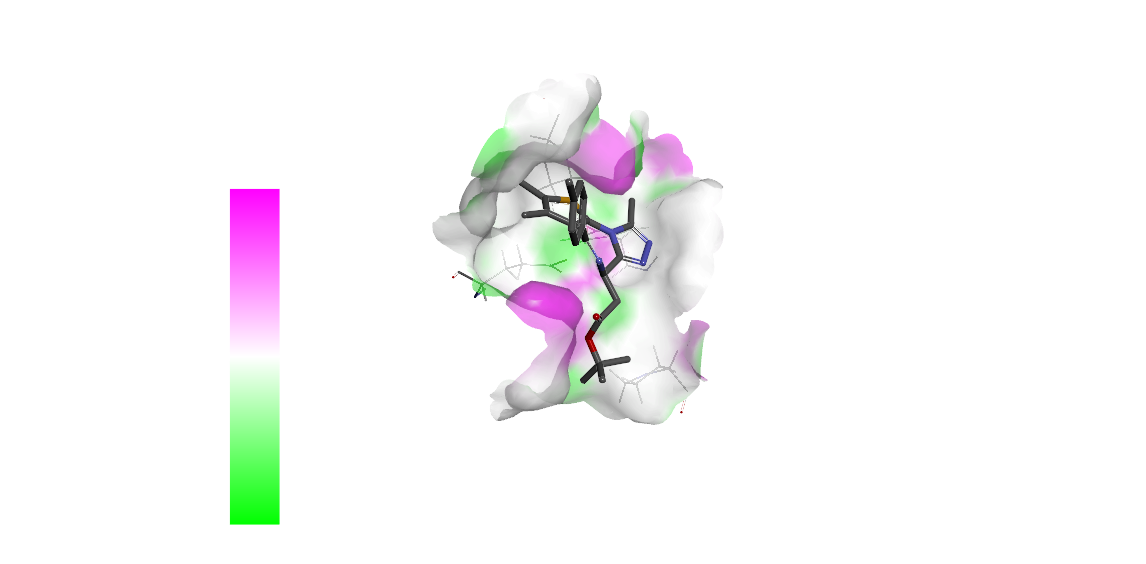 |
| **MET** | -6.44 | 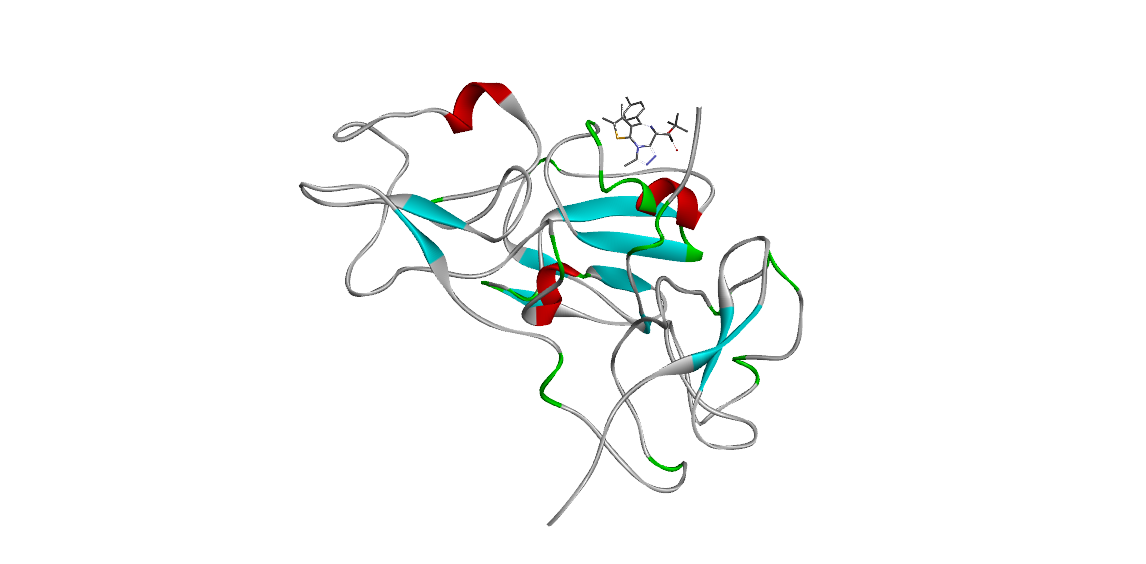 | 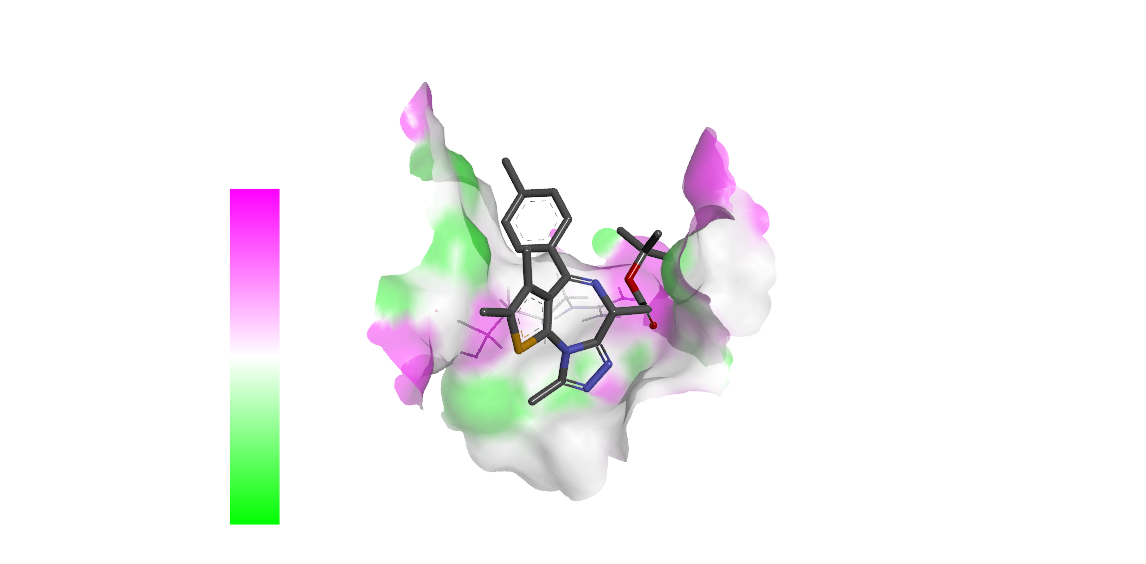 |
| **PSEN2** | -5.42 | 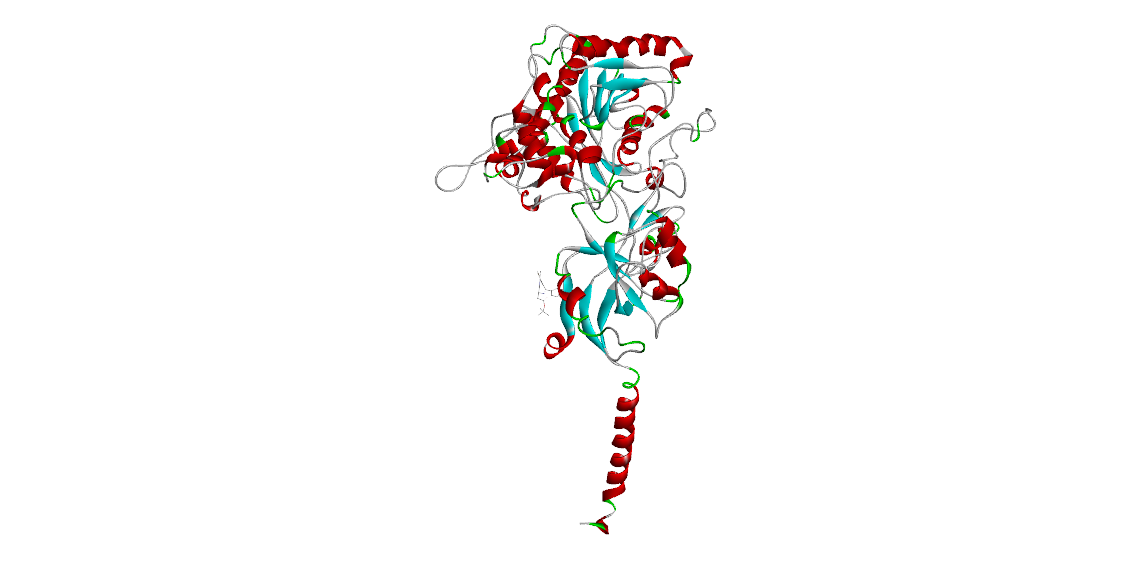 | 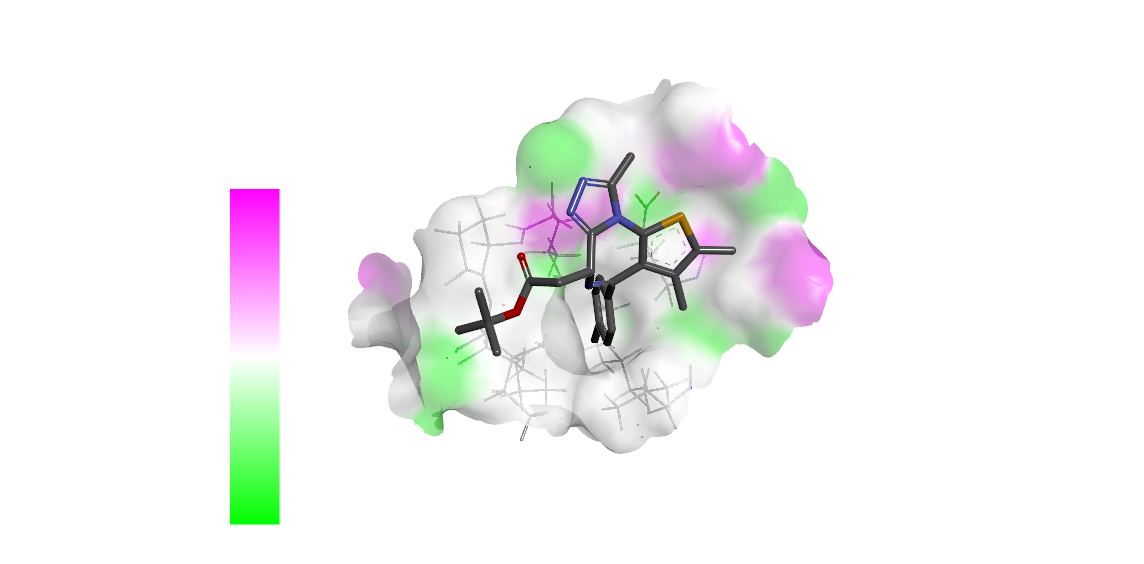 |
| **STAT3** | -4.29 | 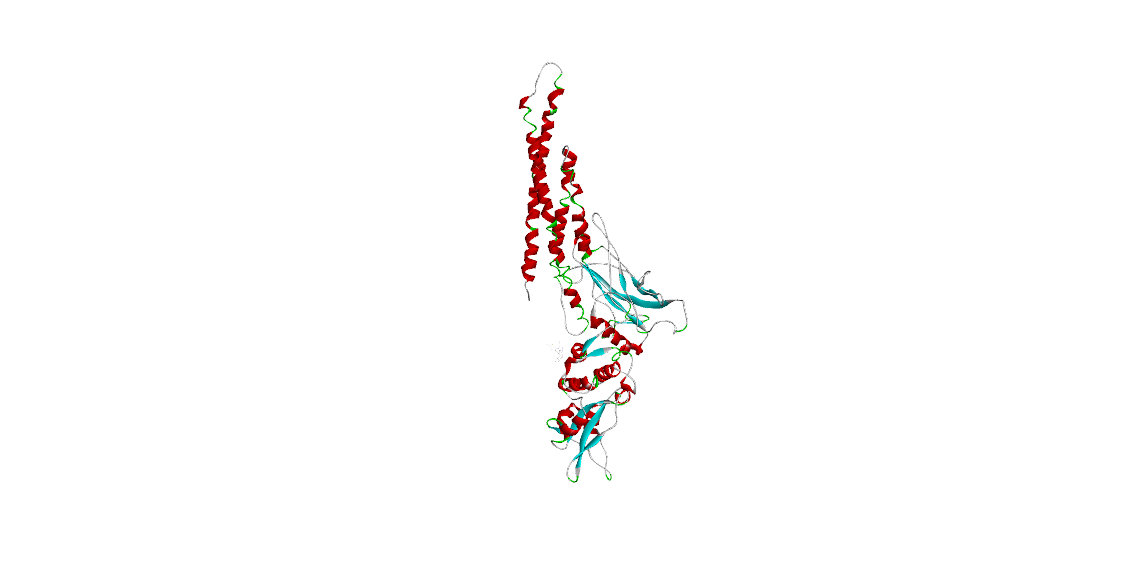 | 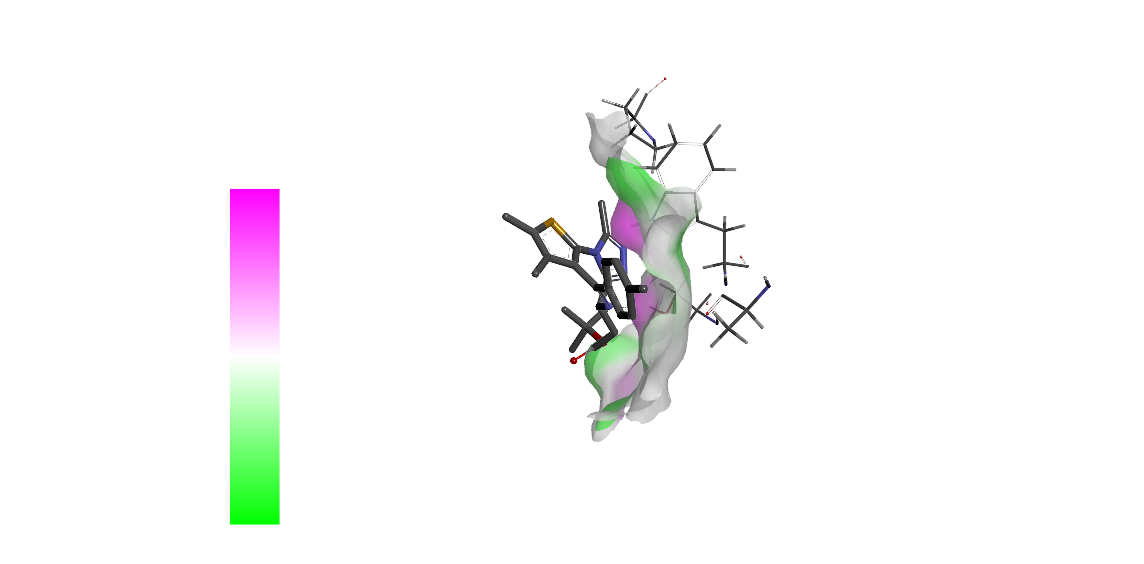 |

**Table S3**. Molecular docking of GSK2801 and GSK2801 against their BC targets. The ligand-protein docking complexes are shown in this table. GSK2801 and GSK2801 are represented as stick models and the target proteins as cartoon models. The ligands are observed to bind in the binding site of target proteins.

| **Protein** | **Docking Score (kcal/mol)** | **Protein-Ligand complex** | **Binding site interactions** |
| --- | --- | --- | --- |
| ABCG2 | -3.48 | 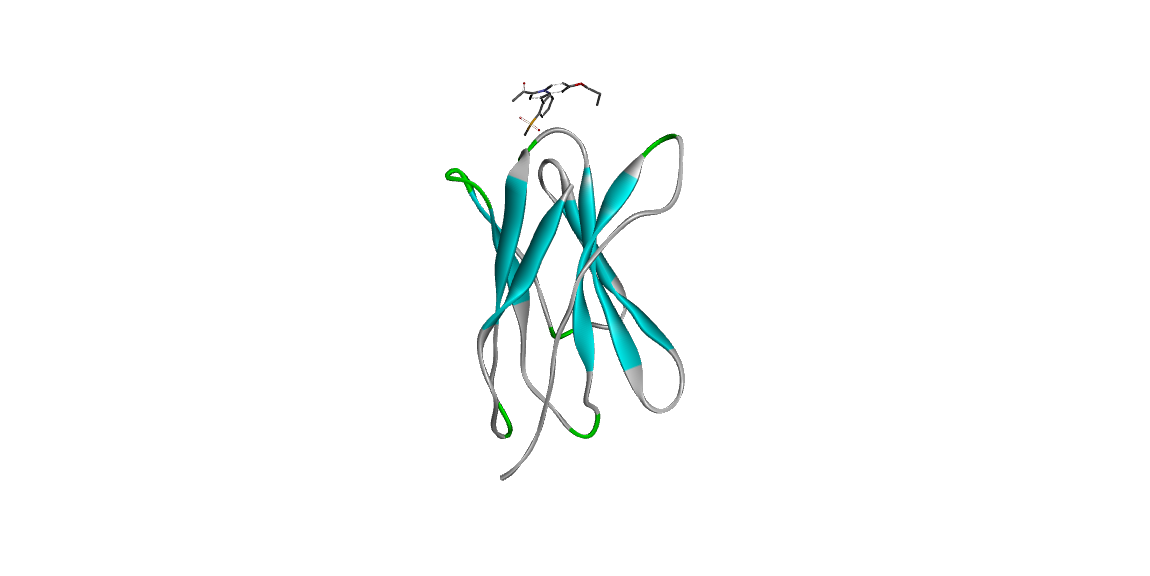 | 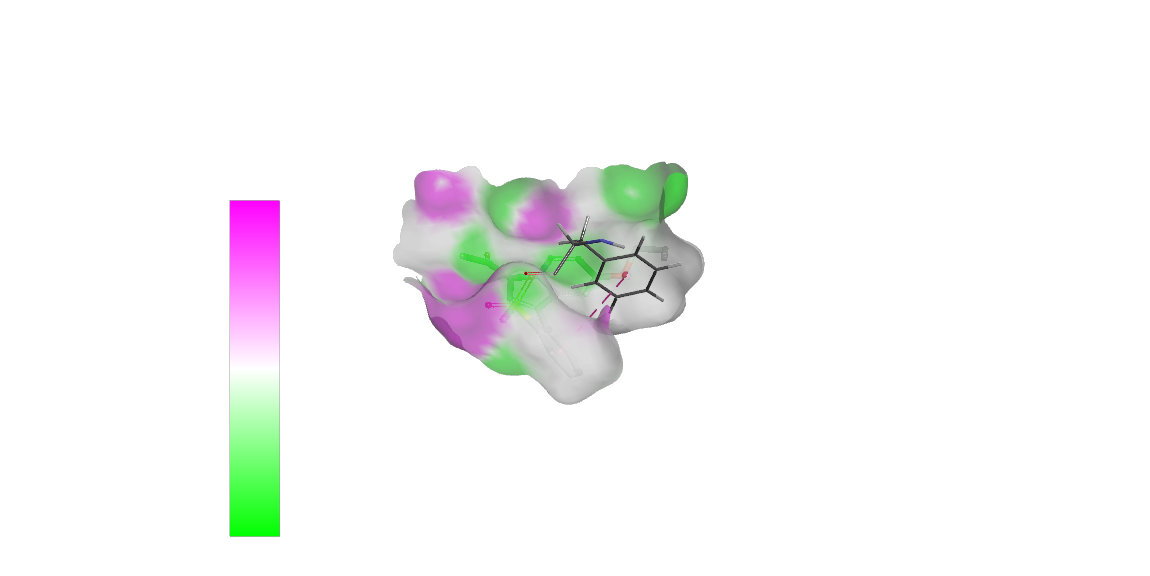 |
| AKT1 | -4.7 | 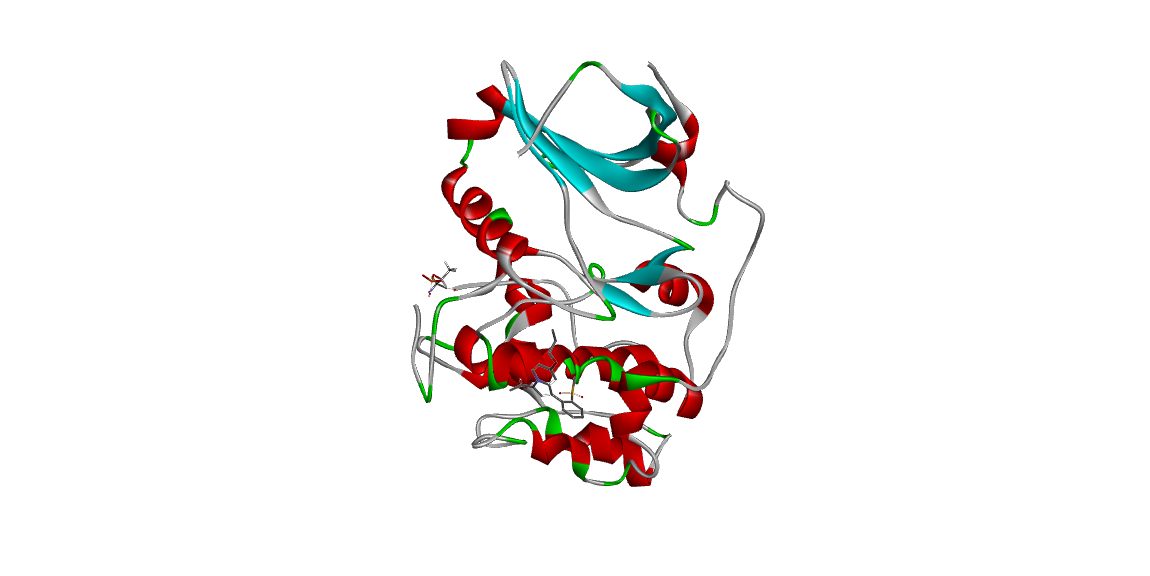 | 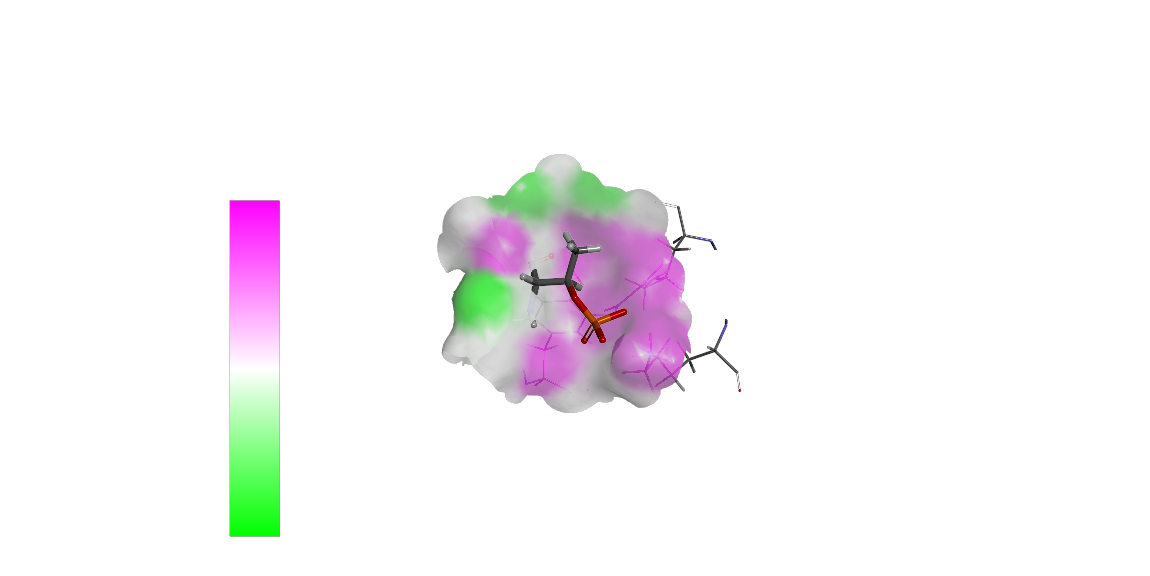 |
| CCND1 | -5.9 | 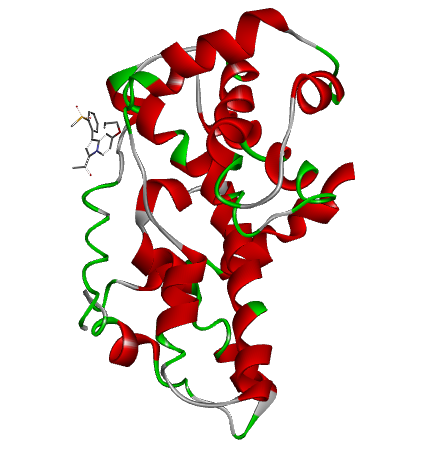 | 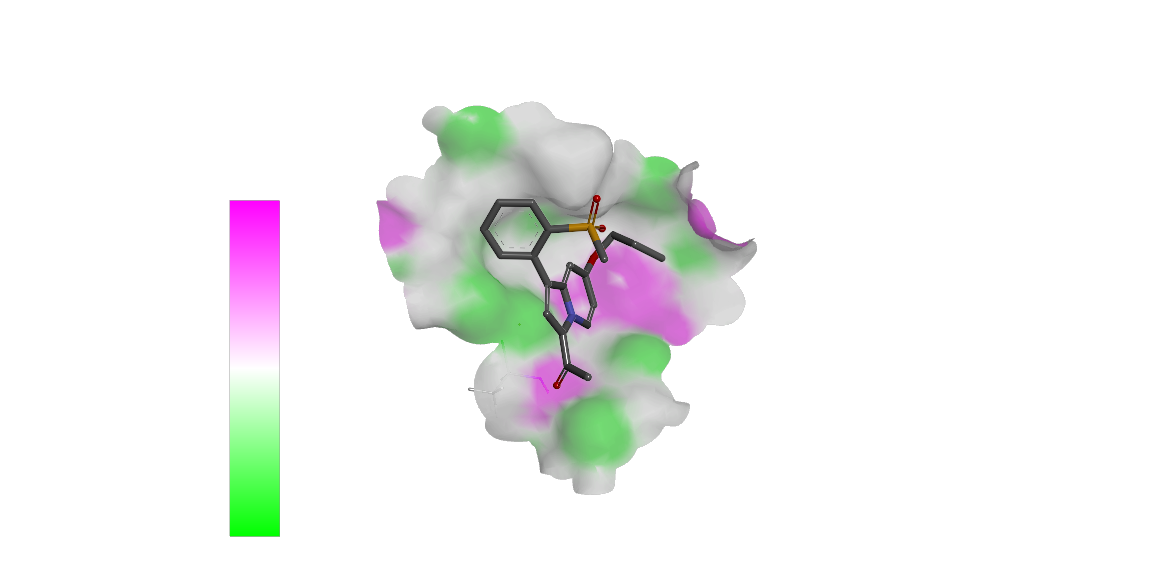 |
| CDK4 | -3.32 | 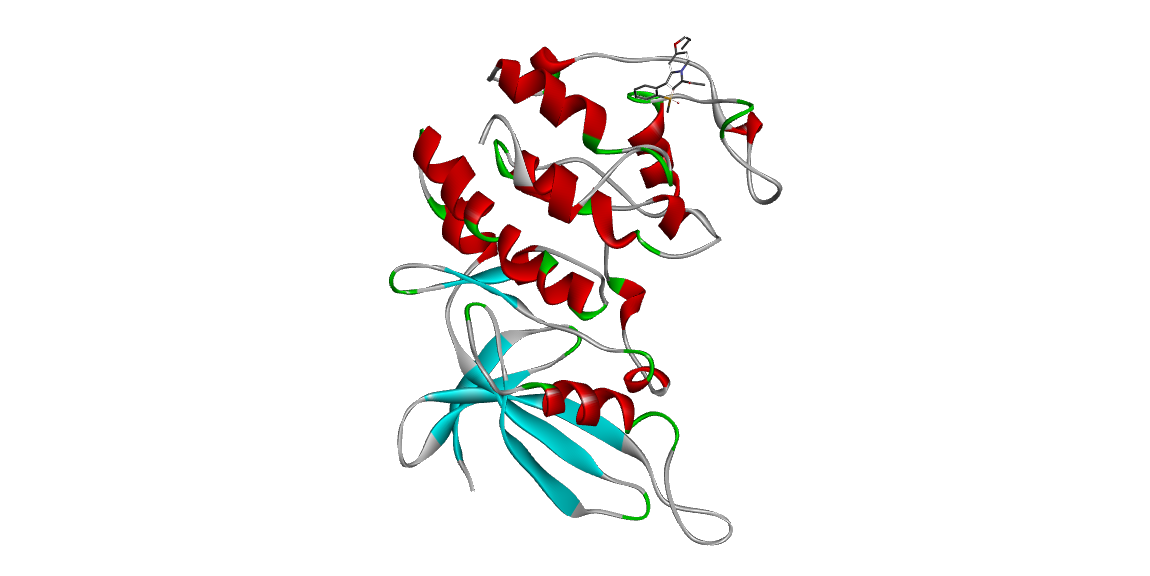 | 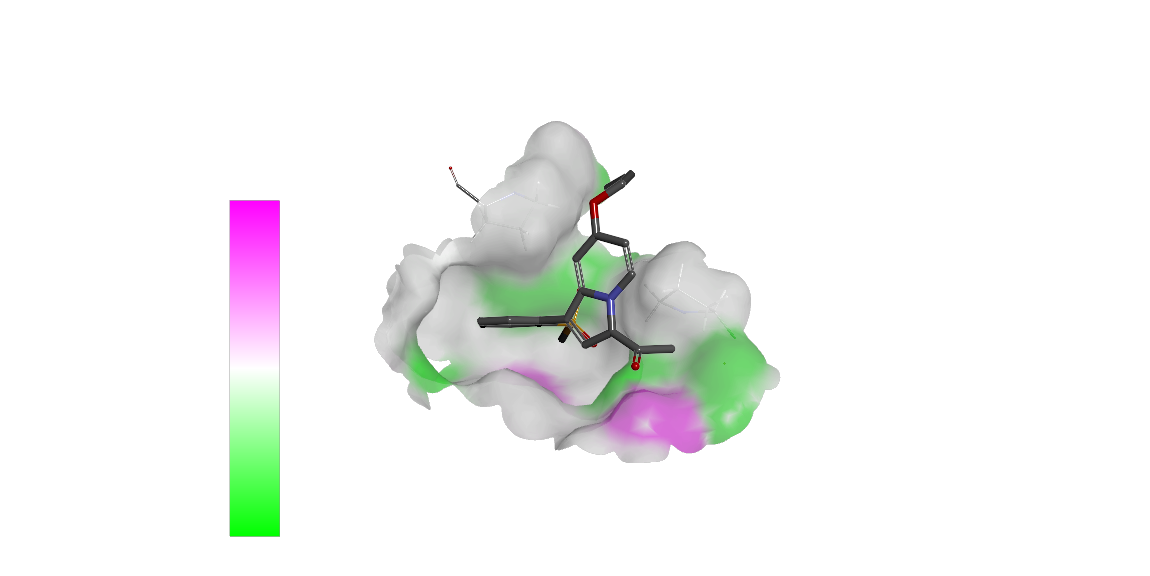 |
| CREBP | -4.56 | 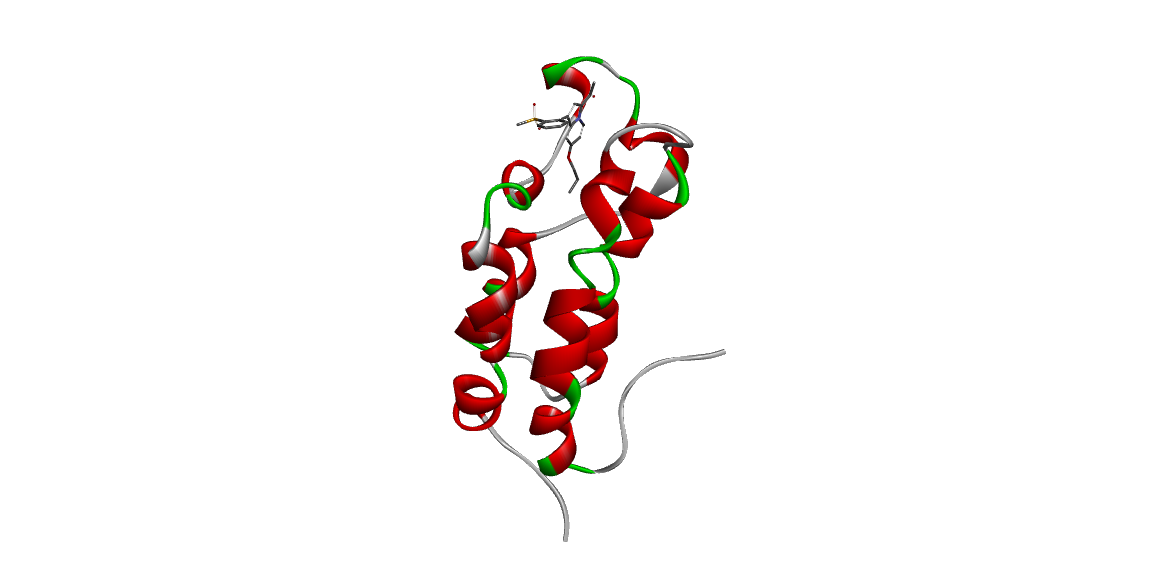 | 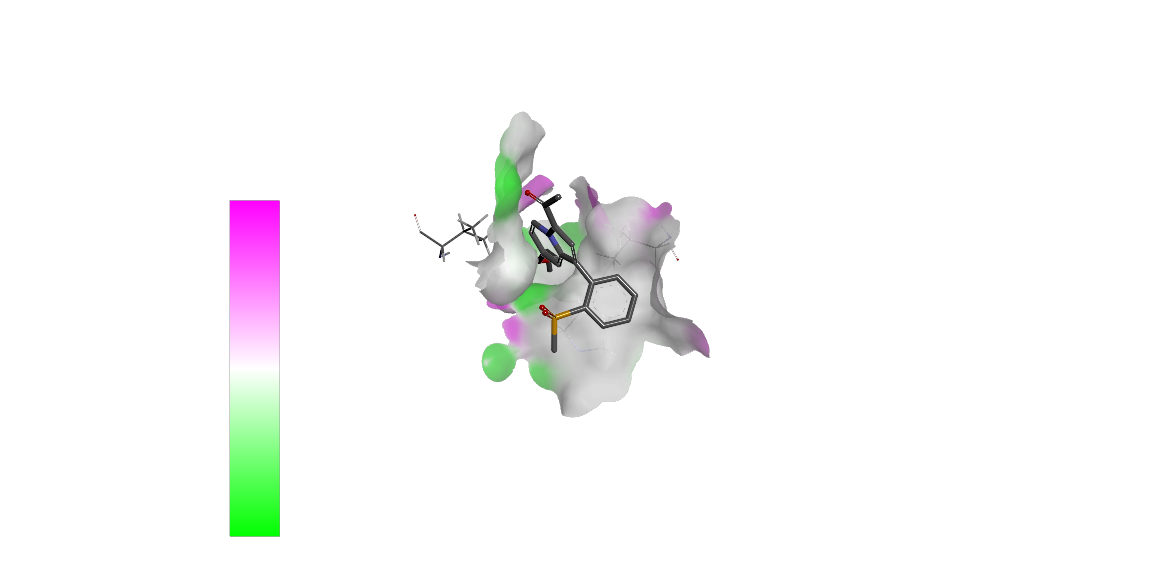 |
| GSK3B | -7.53 | 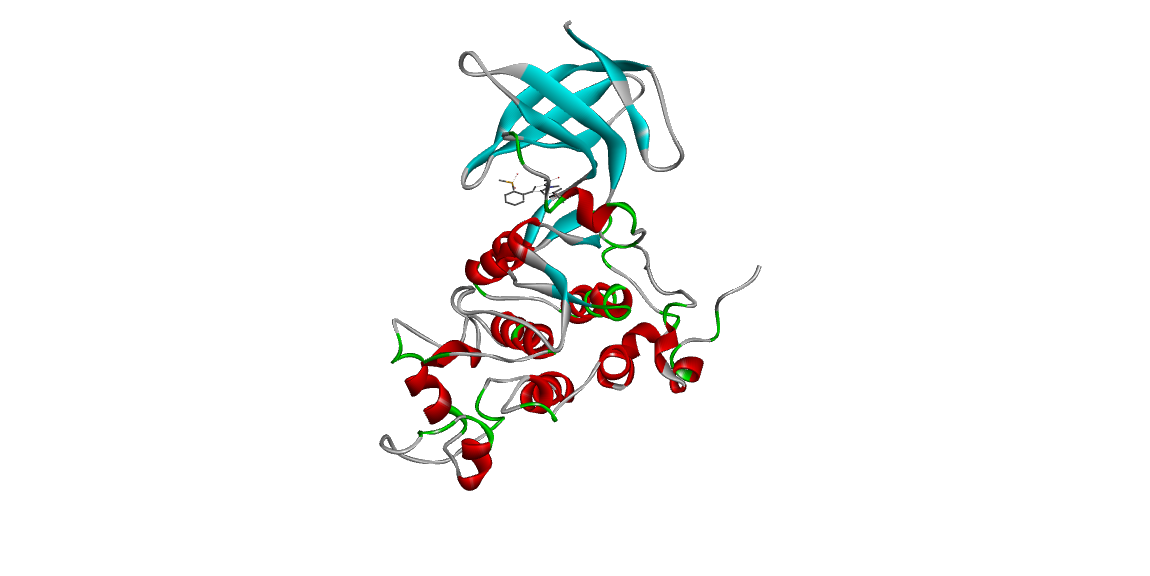 | 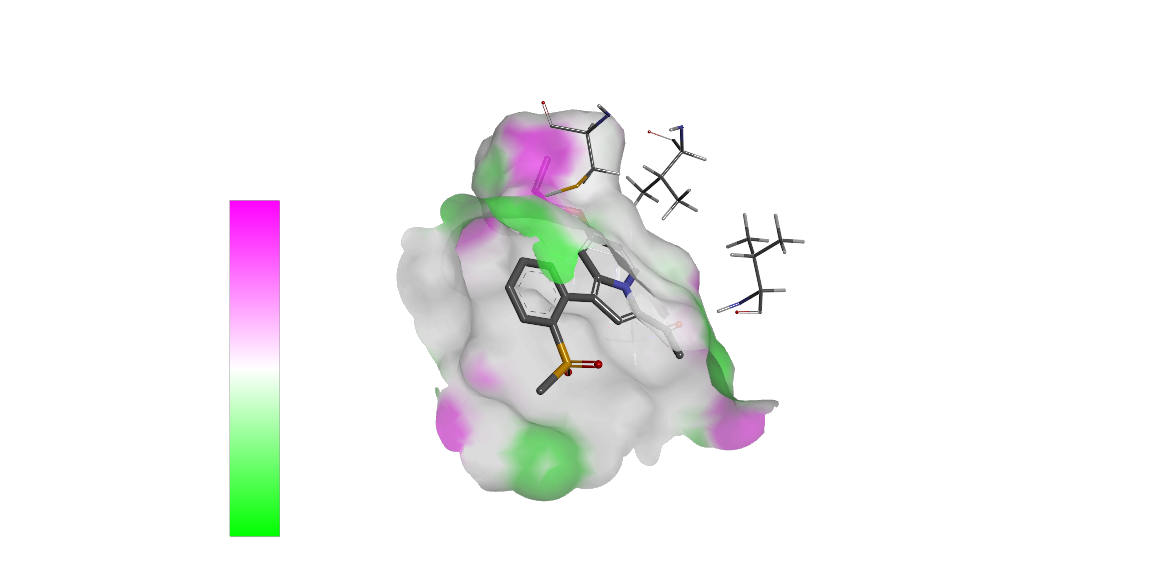 |
| MAP2K1 | -4.44 | 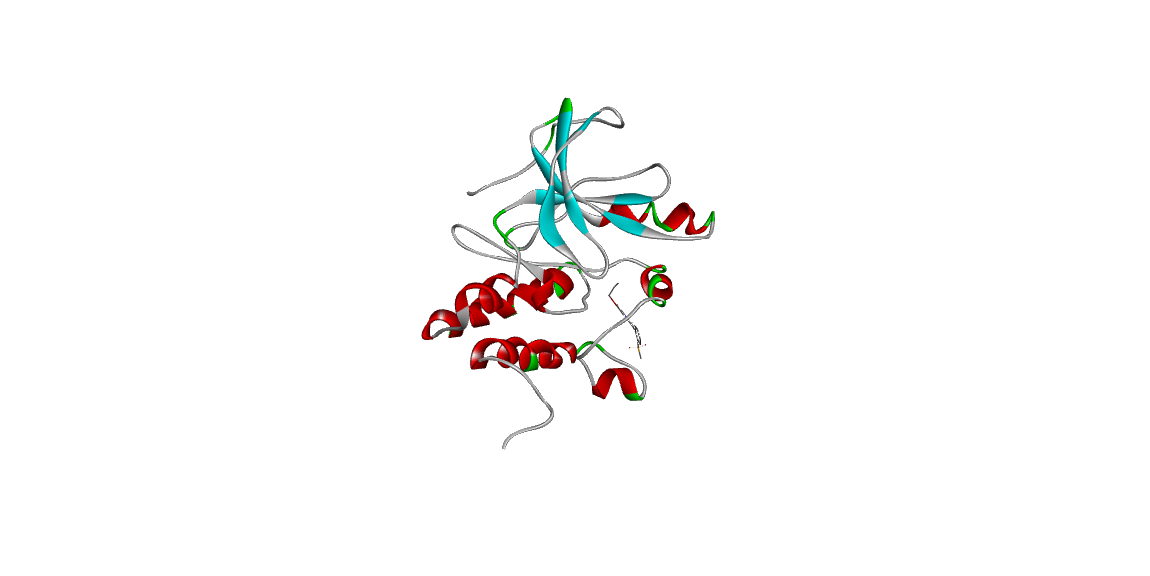 | 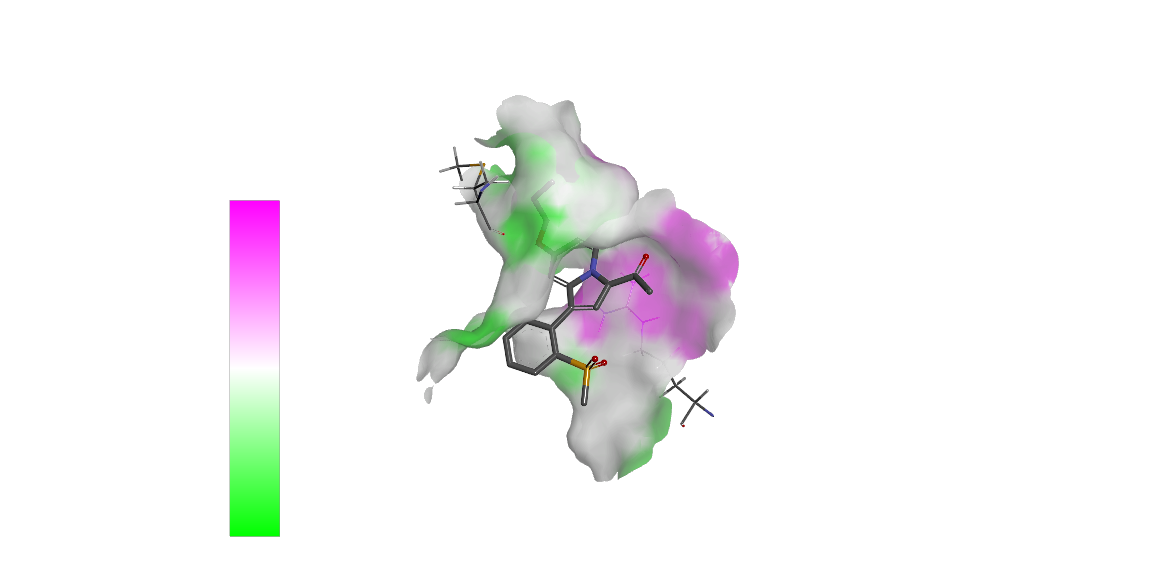 |
| MMP13 | -3.89 | 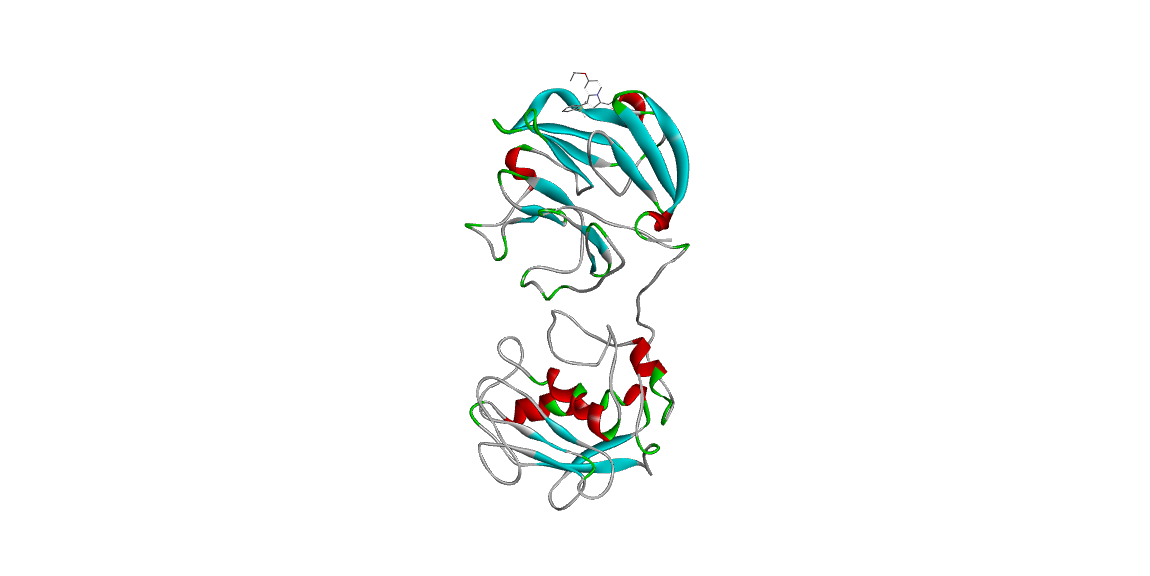 | 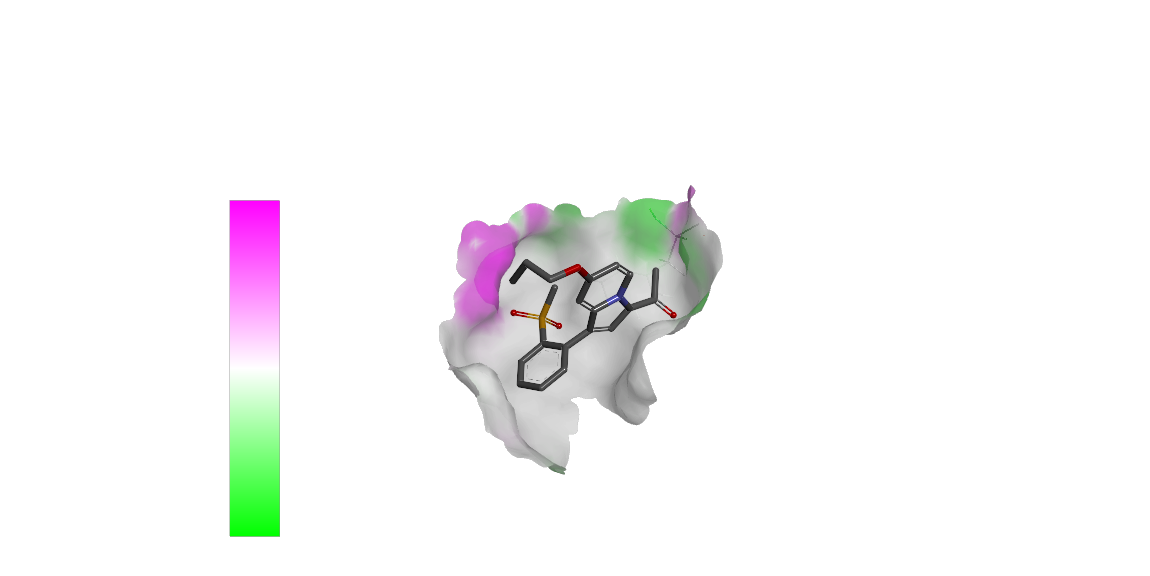 |
| MYLK | -7.62 | 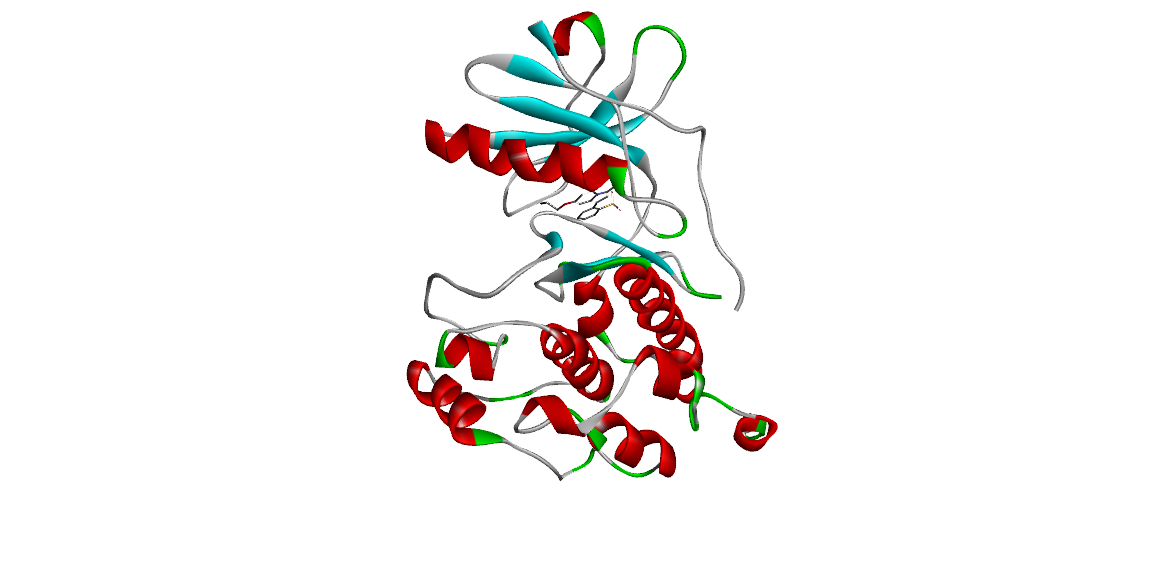 | 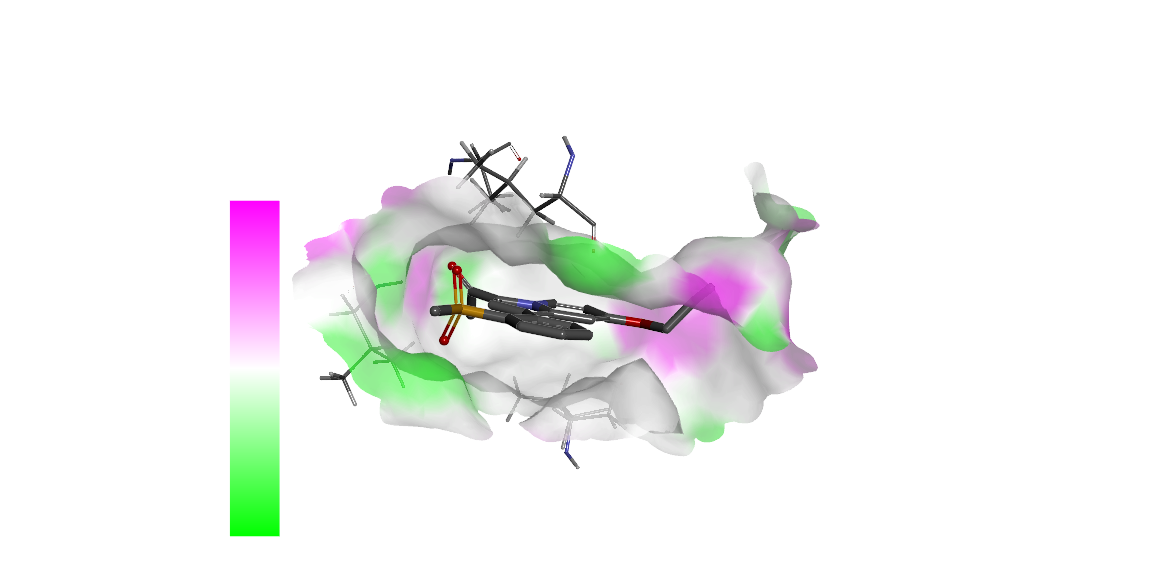 |
| NOS2 | -4.89 | 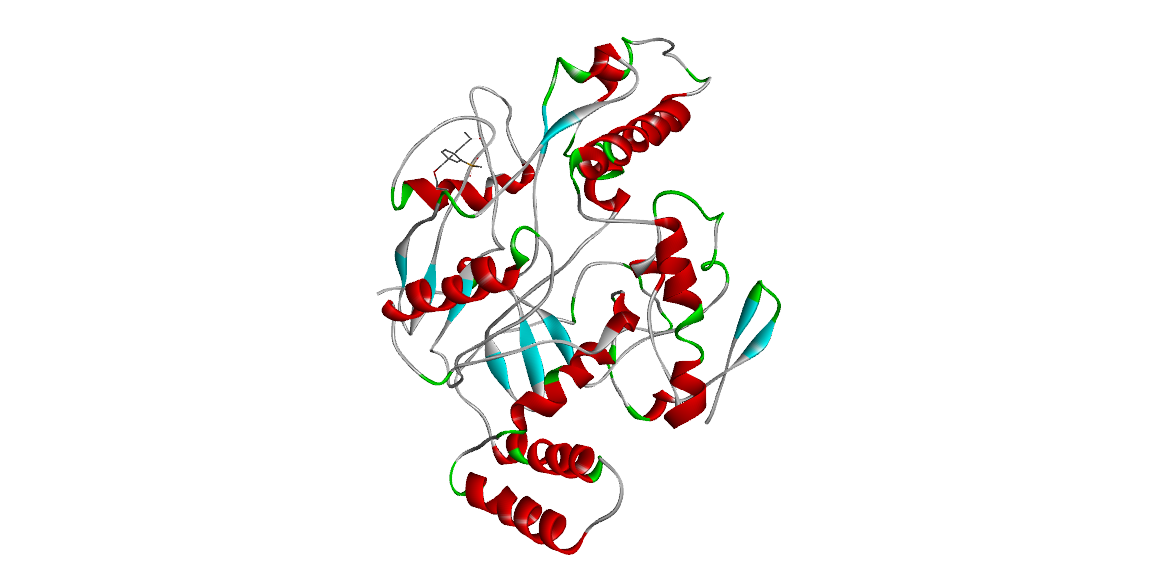 | 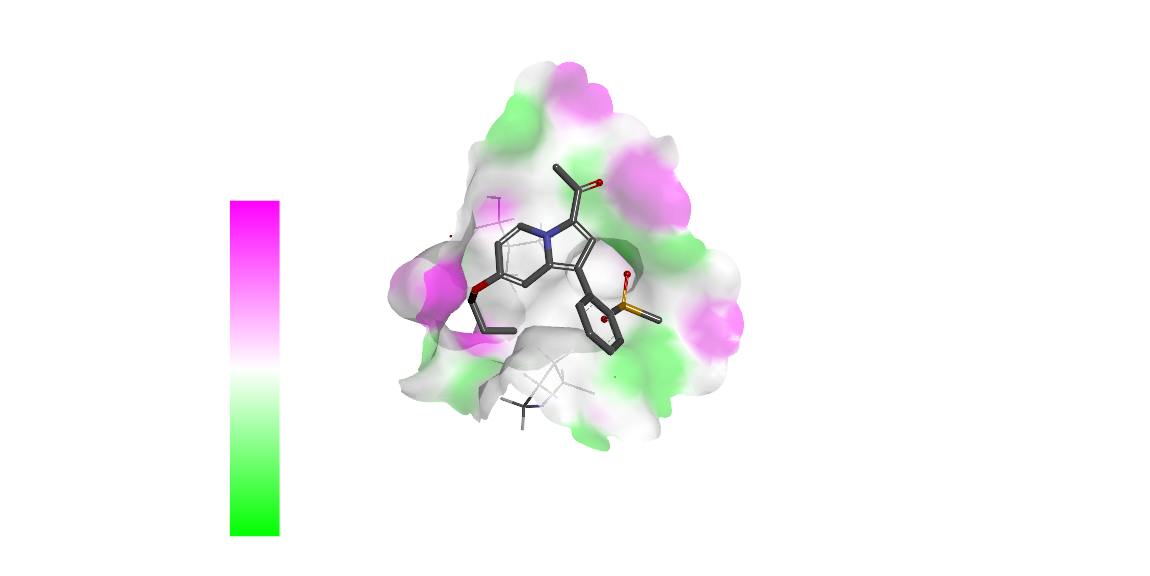 |
| NQO1 | -4.83 | 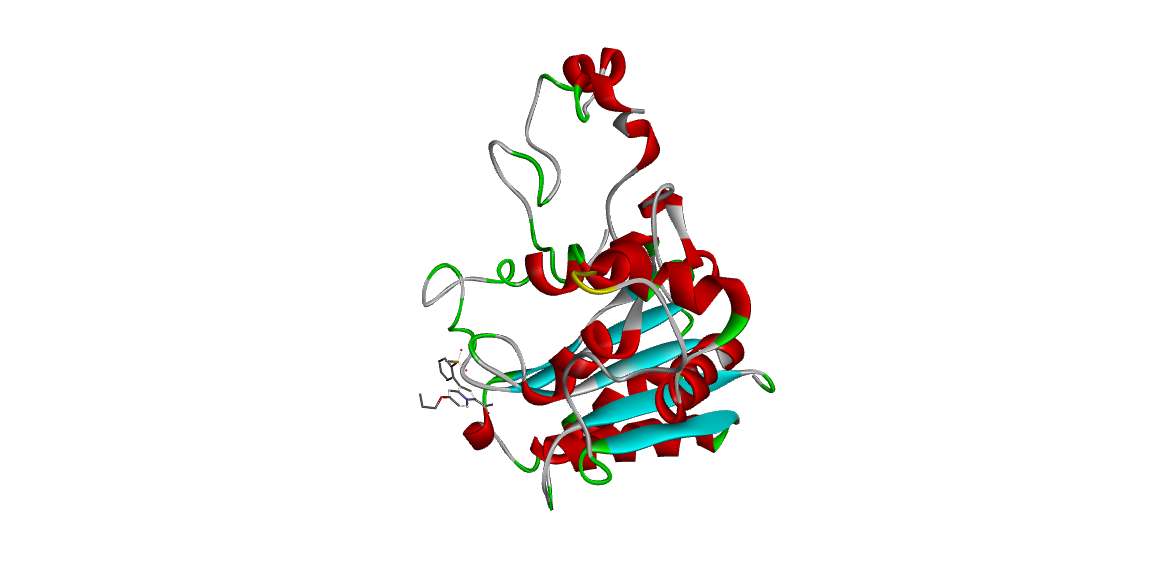 | 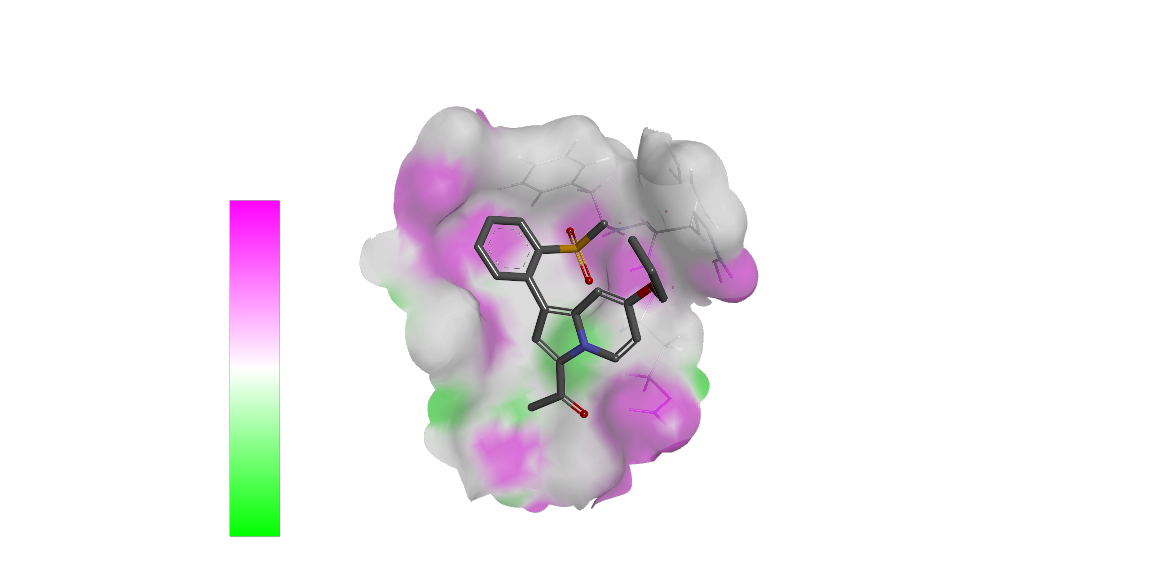 |
| NQO2 | -5.55 | 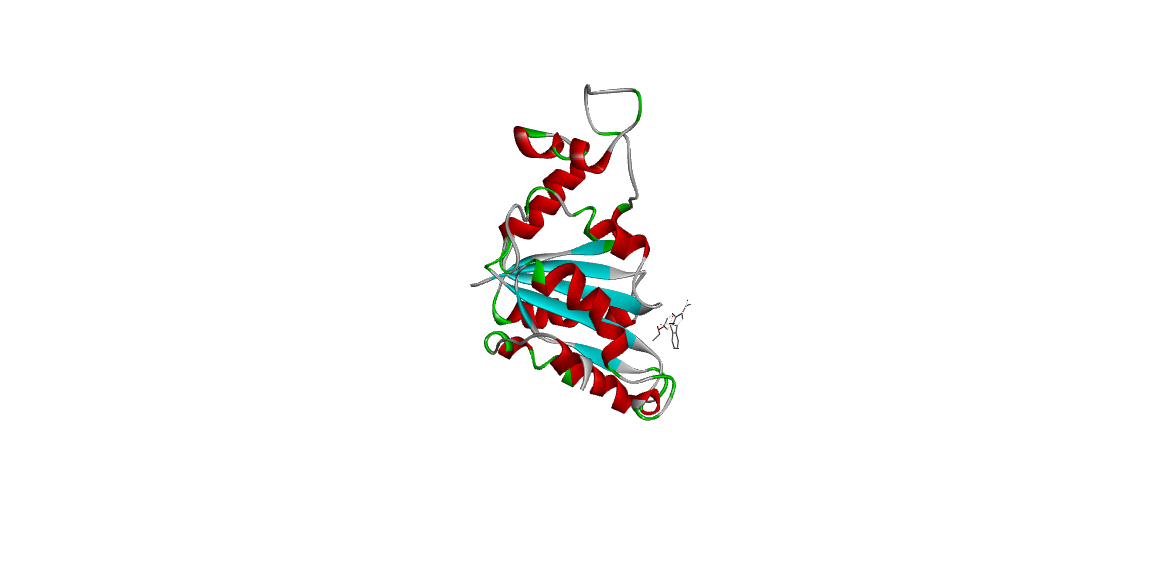 | 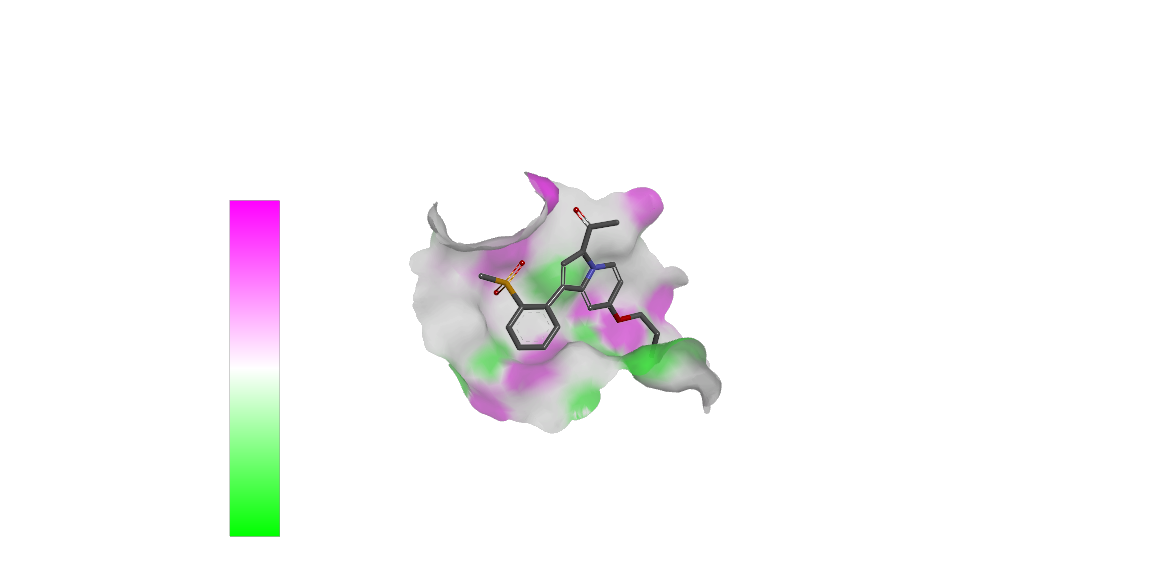 |
| NR1H2 | -6.5 | 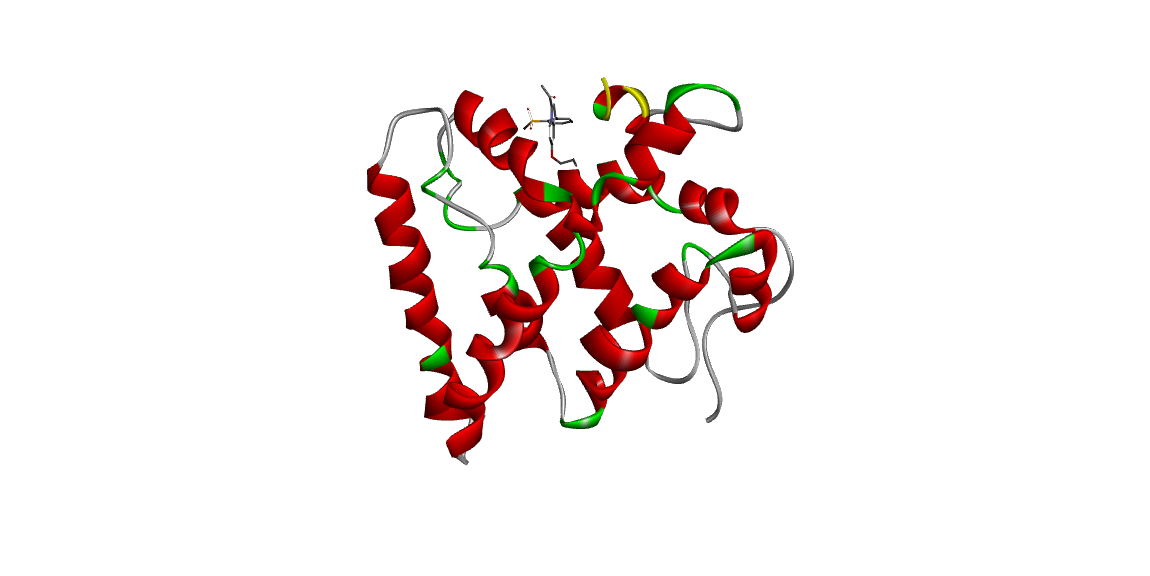 | 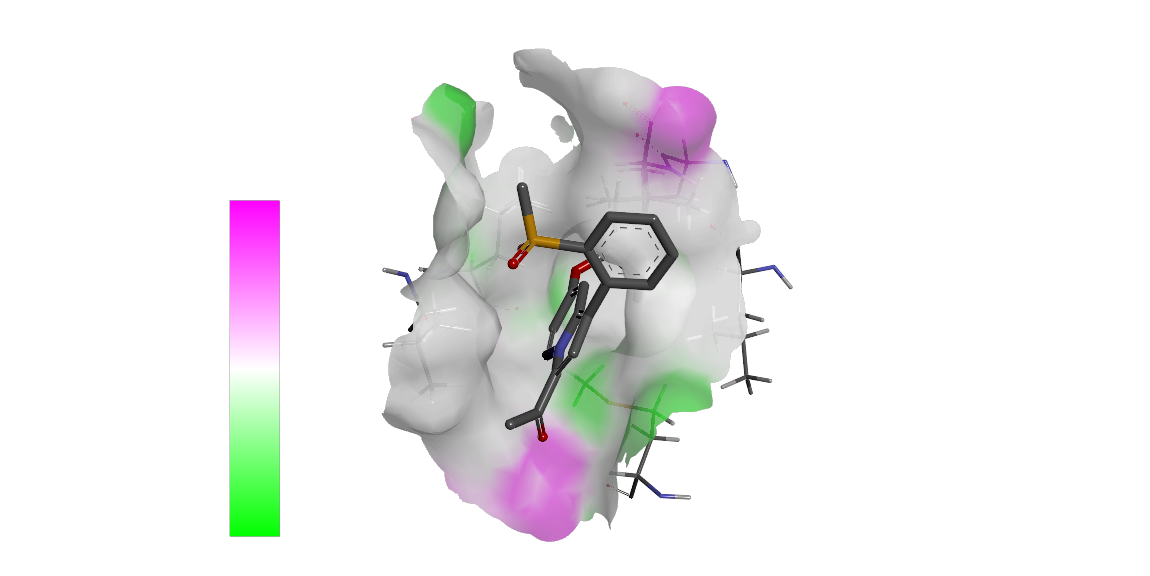 |
| PARP1 | -5.65 | 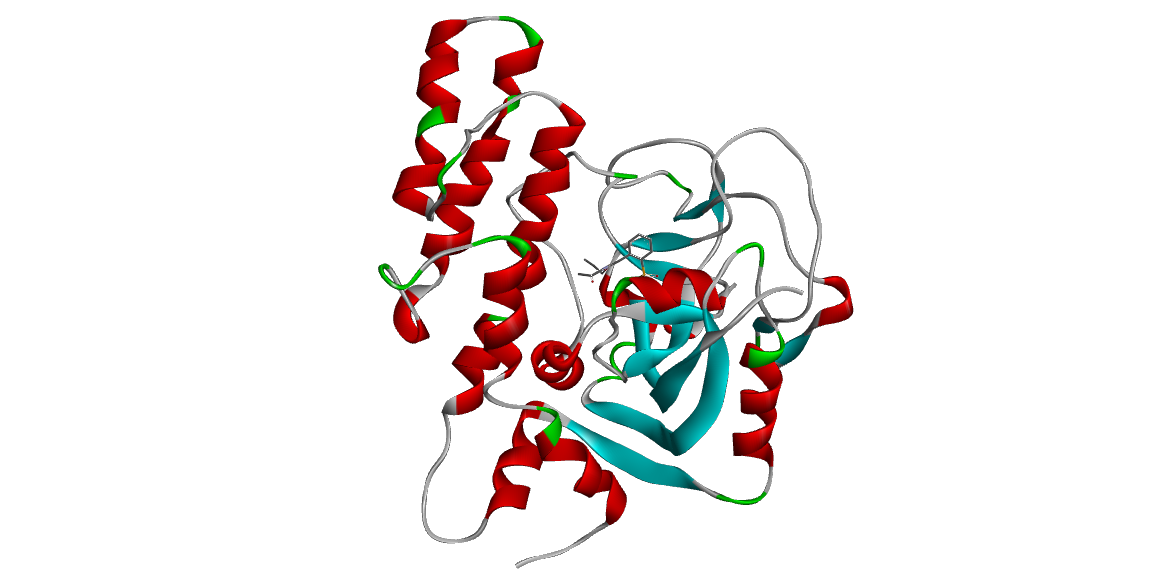 | 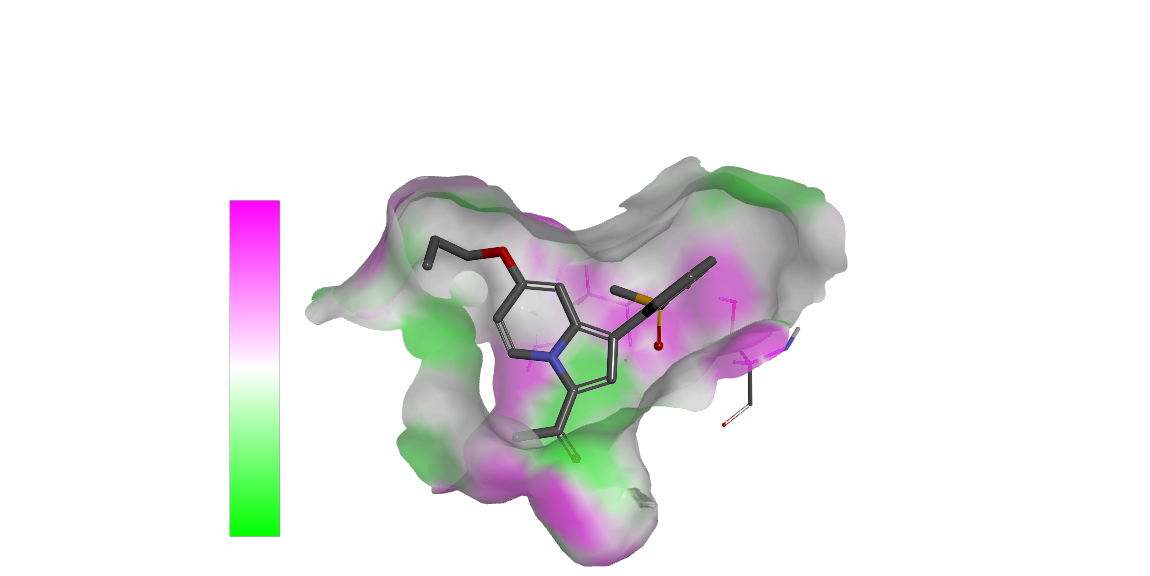 |
| PLAUR | -3.61 | 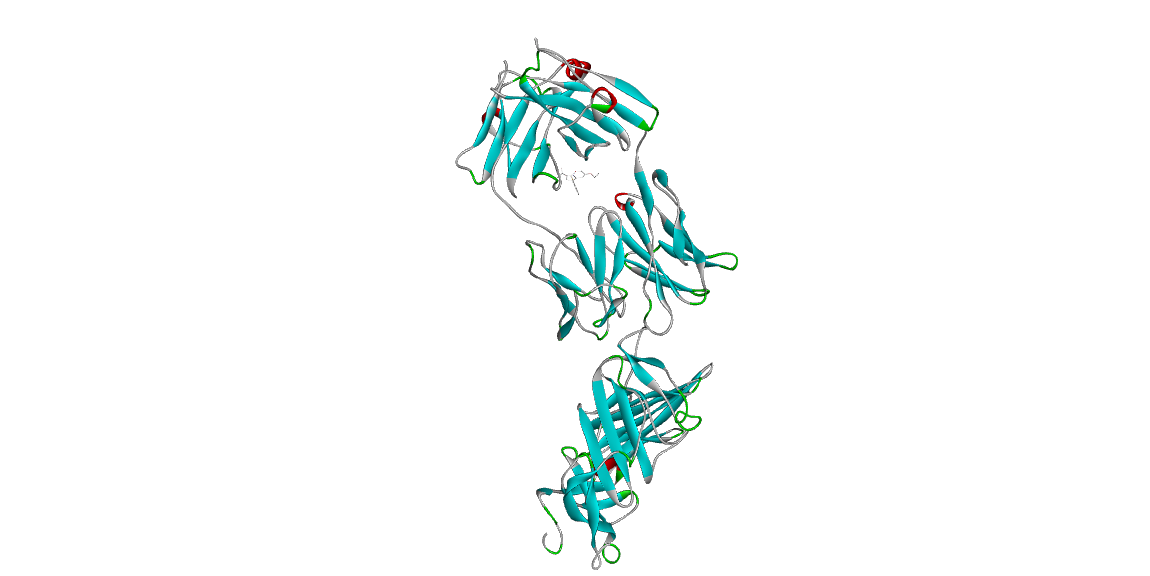 | 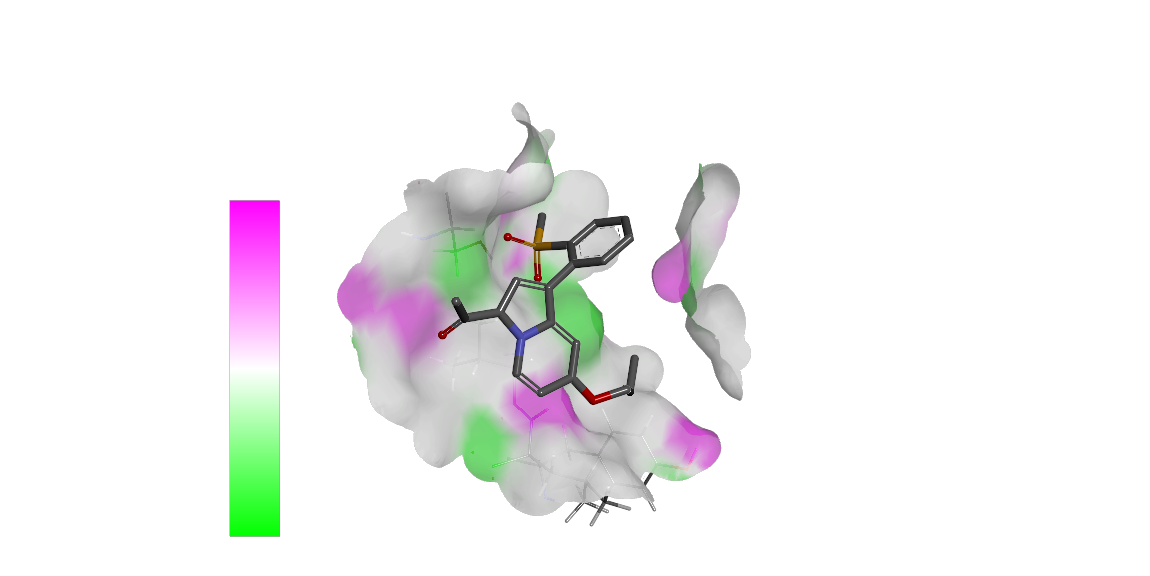 |
| TOP2A | -3.19 | 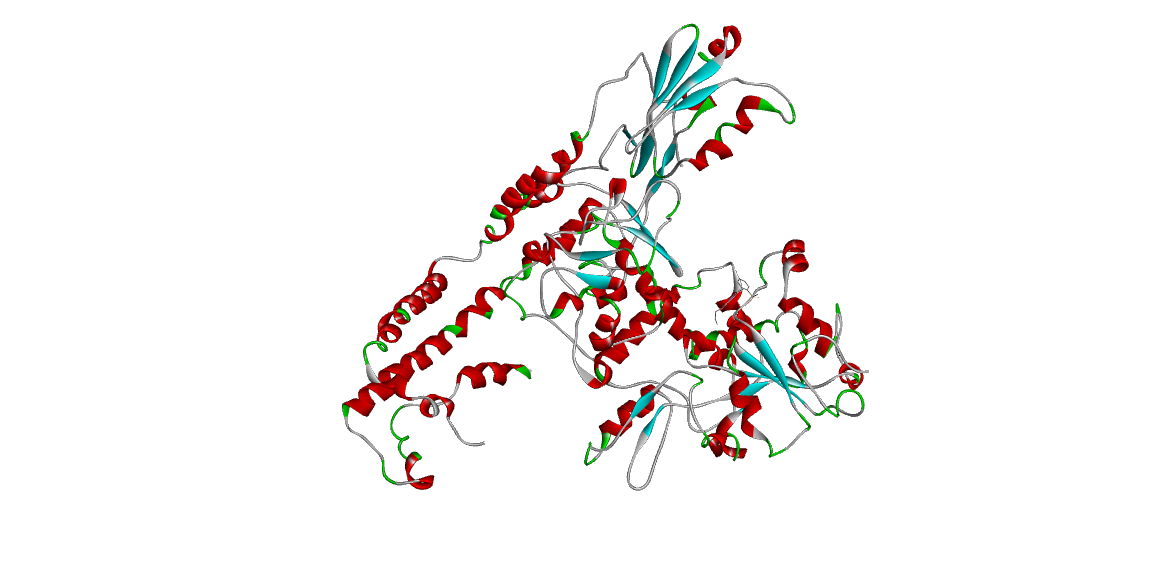 | 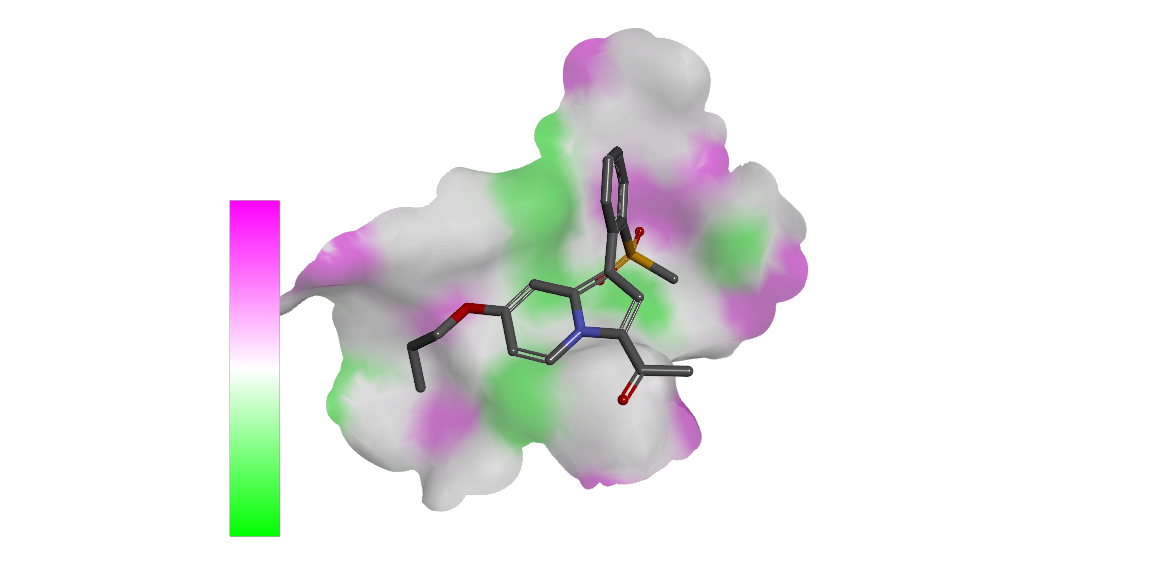 |
| CTSB | -3.47 | 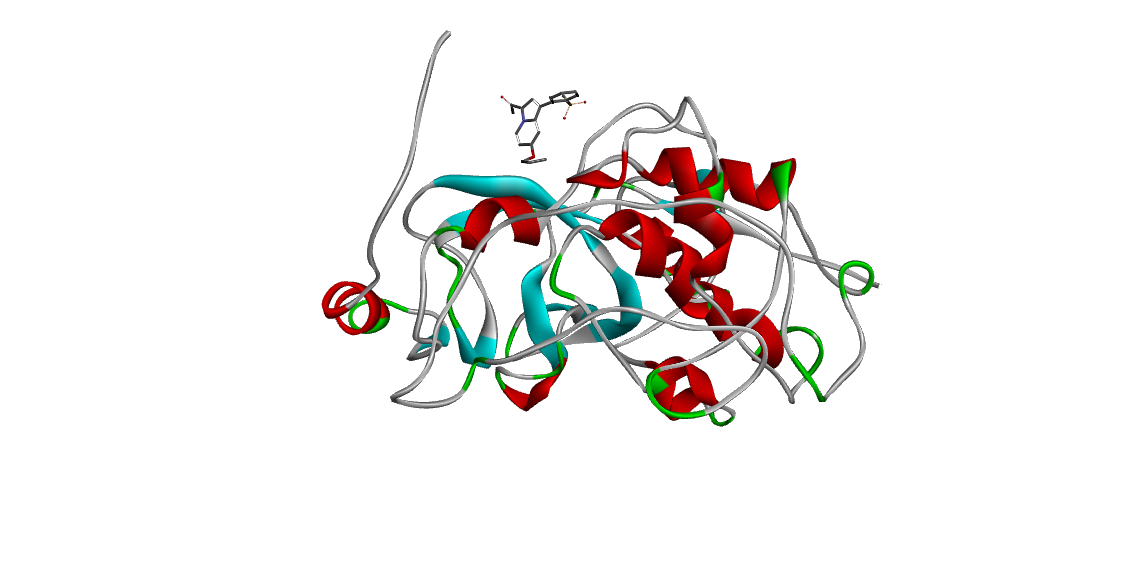 | 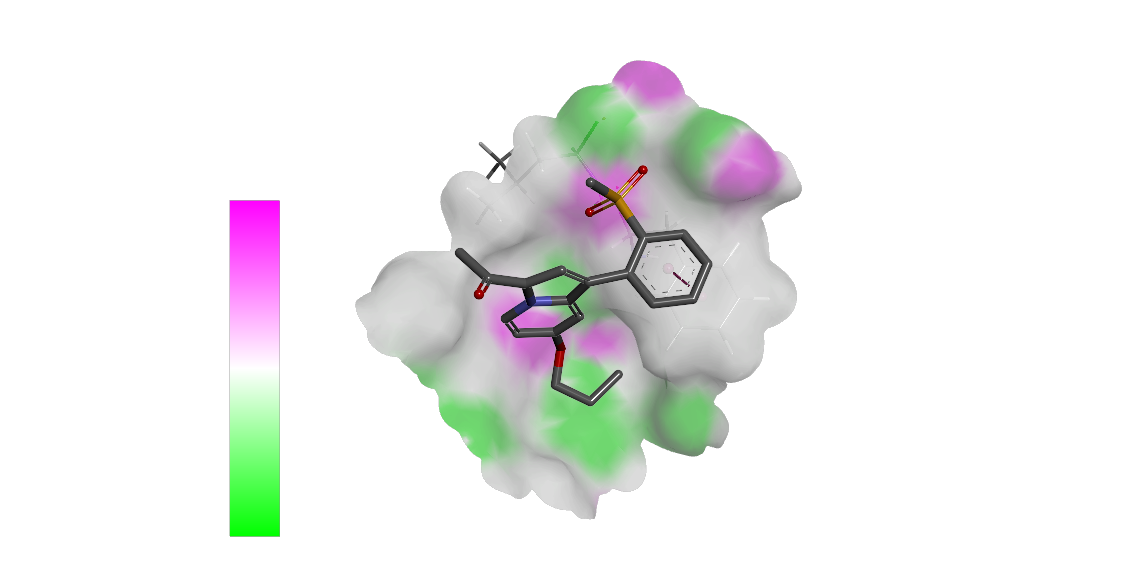 |
| MAPK14 | -3.34 | 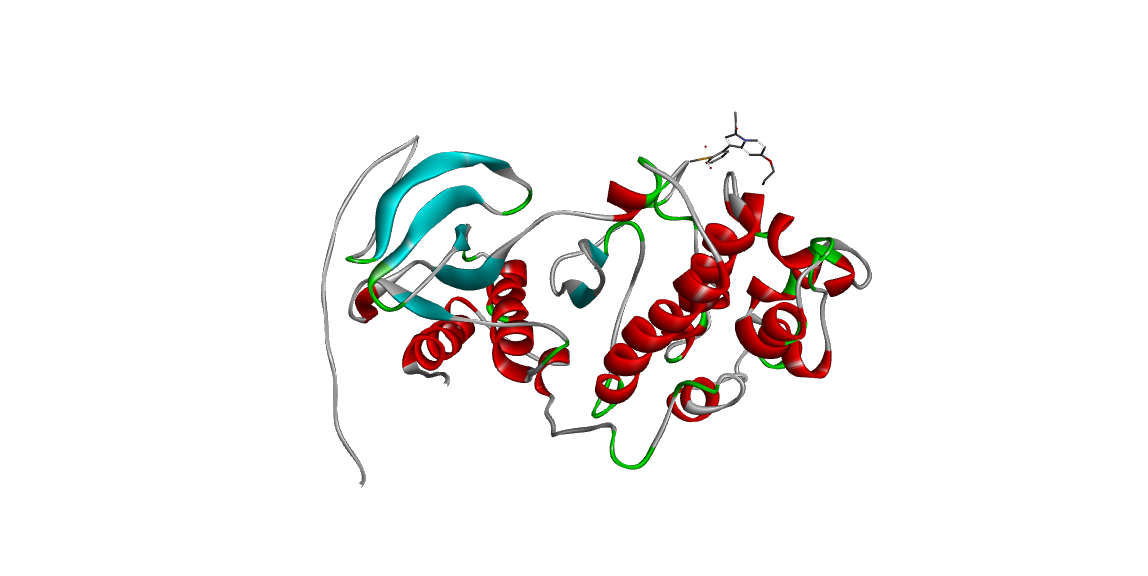 | 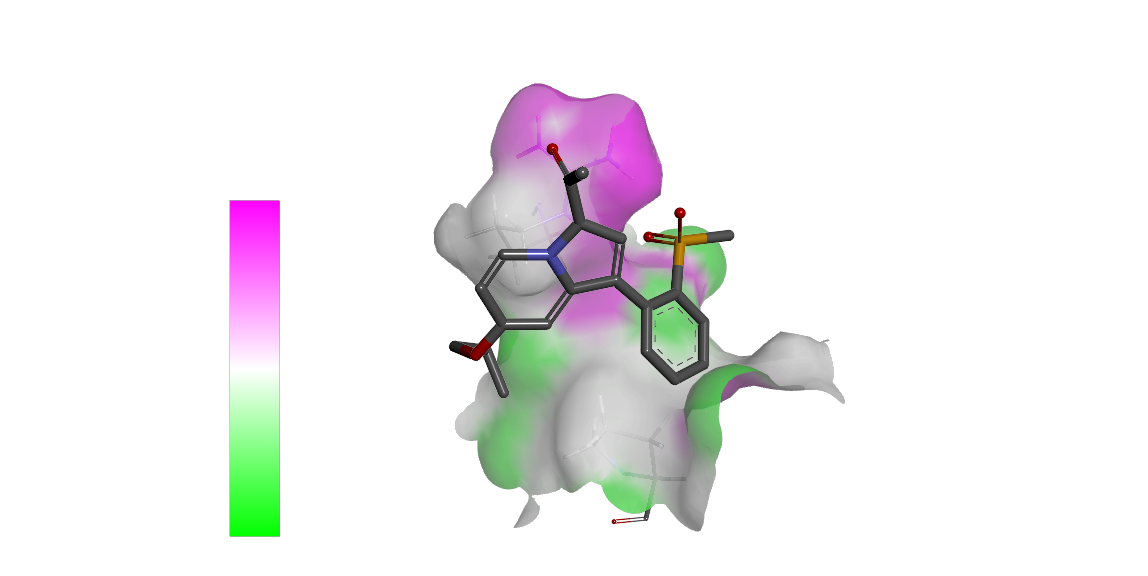 |
| MET | -4.08 |  |  |
| PSEN2 | -3.18 |  |  |
| STAT3 | -3.71 |  |  |
